# Origination, Expansion, Evolutionary Trajectory, and Expression Bias of AP2/ERF Superfamily in *Brassica napus*

**DOI:** 10.3389/fpls.2016.01186

**Published:** 2016-08-12

**Authors:** Xiaoming Song, Jinpeng Wang, Xiao Ma, Yuxian Li, Tianyu Lei, Li Wang, Weina Ge, Di Guo, Zhenyi Wang, Chunjin Li, Jianjun Zhao, Xiyin Wang

**Affiliations:** ^1^Department of Life Sciences, North China University of Science and TechnologyTangshan, China; ^2^Library, North China University of Science and TechnologyTangshan, China; ^3^Key Laboratory of Vegetable Germplasm and Utilization of Hebei, Collaborative Innovation Center of Vegetable Industry in Hebei, College of Horticulture, Agricultural University of HebeiBaoding, China

**Keywords:** AP2/ERF superfamily, polyploid, positive selection, stress tolerance, RNA-seq, *B. napus*

## Abstract

The AP2/ERF superfamily, one of the most important transcription factor families, plays crucial roles in response to biotic and abiotic stresses. So far, a comprehensive evolutionary inference of its origination and expansion has not been available. Here, we identified 515 *AP2/ERF* genes in *B. napus*, a neo-tetraploid forming ~7500 years ago, and found that 82.14% of them were duplicated in the tetraploidization. A prominent subgenome bias was revealed in gene expression, tissue-specific, and gene conversion. Moreover, a large-scale analysis across plants and alga suggested that this superfamily could have been originated from AP2 family, expanding to form other families (ERF, and RAV). This process was accompanied by duplicating and/or alternative deleting AP2 domain, intragenic domain sequence conversion, and/or by acquiring other domains, resulting in copy number variations, alternatively contributing to functional innovation. We found that significant positive selection occurred at certain critical nodes during the evolution of land plants, possibly responding to changing environment. In conclusion, the present research revealed origination, functional innovation, and evolutionary trajectory of the AP2/ERF superfamily, contributing to understanding their roles in plant stress tolerance.

## Introduction

The AP2/ERF superfamily is one of the largest groups of transcription factors in plants, and plays important roles in resistance of abiotic and biotic stress (Licausi et al., 2013). Based on the AP2 domain number, AP2/ERF superfamily can be divided into ERF, AP2, RAV, and Soloist families. The *ERF* genes encode proteins with a single AP2 domain, while *AP2* genes encode protein with two AP2 domains (Nakano et al., 2006; Licausi et al., 2010). According to DNA binding domain sequences, the ERF family can be further divided into two subfamilies, ERF and DREB subfamilies. Besides a single AP2 domain, the RAV family have an additional B3 domain (Hu and Liu, 2011). In addition, there are genes divergent from the ERF and RAV families, though containing the AP2 domains, and here they are named as Soloist according to the previous report (Sakuma et al., 2002).

In that its pivotal importance to plant tolerance, the AP2/ERF superfamily has been identified and investigated in many plants, including *Arabidopsis thaliana, Oryza sativa* (Nakano et al., 2006), *Brassica rapa* (Song et al., 2013), *Brassica oleracea* (Thamilarasan et al., 2014), *Populus trichocarpa* (Zhuang et al., 2008), *Vitis vinifera* (Licausi et al., 2010), *Cucumis sativus* (Hu and Liu, 2011), *Triticum aestivum* (Zhuang et al., 2011a), *Glycine max* (Zhang et al., 2008), and *Hordeum vulgare* (Gil-Humanes et al., 2009), *Hevea brasiliensis* (Duan et al., 2013), *Arabidopsis lyrata, Capsella rubella, Eutrema salsugineum*, and *Carica papaya* (Zeng et al., 2016).

The DREB subfamily activates dehydration/cold-regulated genes by interacting with DRE/CRT elements, and therefore enhances tolerance to multiple abiotic stresses (Lata and Prasad, 2011). For example, *DREB2* genes are involved in dehydration- and high-salinity-responsive gene expression in transgenic *Arabidopsis* (Nakashima et al., 2000). The *EsDREB2B* gene cloned from *Eremosparton songoricum* is shown to be able to enhance tolerance to multiple abiotic stresses in yeast and transgenic tobacco (Li et al., 2014). The *Suaeda salsa SsDREB* gene enhances abiotic stress tolerance in transgenic tobacco (Zhang et al., 2015). ERF subfamily is involved in signal pathways of stress, pathogen, and disease-related stimuli (Cheng et al., 2013; Schmidt et al., 2013; Shoji et al., 2013; Zhu et al., 2014). In transgenic plants, the over-expression of *ERF* genes has been reported in *O. sativa* (Zhang et al., 2010), *A. thaliana* (Wang et al., 2015a), *Solanum lycopersicum*, and *Nicotiana tabacum* (Zhang and Huang, 2010), leading to salt and drought tolerance. Most ERF family genes improve abiotic tolerance without causing undesirable growth phenotypes (Xu et al., 2011). Besides, *CRL5*, an *AP2* gene, promotes crown root initiation in rice (Kitomi et al., 2011). *CRL5* can also affect sepal abscission, leaf shape, and plant height in *B. napus*, maize, and water lily (Jiang et al., 2012; Luo et al., 2012; Yan et al., 2012). *CaRAV1* cloned from *Capsicum annuum* increases tolerance to osmotic stress and high salinity in *Arabidopsis*, and *RAV-1-HY15* gene in *B. napus* can be induced by cold, PEG, and NaCl treatments (Lee et al., 2010; Zhuang et al., 2011b). Therefore, it is important to identify all *AP2/ERF* genes to reveal mechanisms underlying stress signal transmission, and finally manipulate AP2/ERF protein regulation to improve plant stress resistance.

As an important oilseed crop grown worldwide, the genome of *B. napus* was recently sequenced and assembled (Chalhoub et al., 2014). *B. napus* (AACC genome), an allopolyploid, is originated by hybridization between *B. rapa* (AA genome), and *B. oleracea* (CC genome) only ~7500 years ago (Chalhoub et al., 2014). The availability of these *Brassica* genomes, together with those of endicot relatives, *A. thaliana, P. trichocarpa*, and *V. vinifera* etc (Tuskan et al., 2006; Jaillon et al., 2007; Wang et al., 2011; Lamesch et al., 2012; Cheng et al., 2014; Liu et al., 2014; Parkin et al., 2014), provides us an opportunity to understand the formation and evolution of AP2/ERF superfamily and may help clarify molecular mechanisms responsible for abiotic and biotic stress responses.

## Materials and methods

### Retrieval of genome sequences

The genome sequences of *B. napus* were downloaded from the Genoscope genome database (ftp://brassicadb.org/Brassica_napus/; Chalhoub et al., 2014), *B. rapa* sequences from BRAD (http://brassicadb.org/brad/; Cheng et al., 2011), *B. oleracea* sequences from EMBL (http://www.ebi.ac.uk/), *Arabidopsis* sequences from TAIR (http://www.arabidopsis.org/), rice sequences from RGAP (http://rice.plantbiology.msu.edu/; Kawahara et al., 2013), and *A. trichopoda* sequences from Amborella Genome Database (http://amborella.huck.psu.edu/; Albert et al., 2013). The sequences of the other 9 species were downloaded from JGI (http://www.phytozome.net/; Goodstein et al., 2012). These selected plants can represent certain major branches of land plants.

### Identification and characterization of AP2/ERF superfamily genes

Pfam database was used to identify genes from AP2/ERF superfamily (Finn et al., 2014), and AP2 domain has Pfam accession number PF00847.16. Genes containing AP2 were defined as AP2/ERF superfamily, and further verified using SMART (Letunic et al., 2012). Gene structures were checked by GSDS (http://gsds.cbi.pku.edu.cn/; Hu et al., 2015). Chromosomal distribution of *B. napus* genes was displayed using an in-house-developed Perl script. The number of exon and intron were showed by Circos (http://circos.ca/; Krzywinski et al., 2009). The AP2 domains of the protein sequences were used to construct phylogenetic trees. Phylogenetic analyses were conducted using MEGA 6.0 (Tamura et al., 2013). Neighbour-joining (NJ) trees were constructed with a bootstrap value of 1000 replications to assess the reliability of the resulting trees. In addition, the maximum-likelihood phylogenetic trees (ML) were constructed using JTT model with the bootstrap value of 1000 by PhyML program (Guindon et al., 2010).

### Identification of orthologs and paralogs

Orthologous and paralogous AP2/ERF superfamily genes were identified using OrthoMCl (http://orthomcl.org/orthomcl/; Li et al., 2003); relationship between them were shown using Circos (Krzywinski et al., 2009), interaction networks were constructed using Cytoscape (Cline et al., 2007). Clustering analyses was performed using MCL (−I > 1.5) and the clustering results were shown by Venn diagrams.

### Identification of collinear blocks and gene conversion

Firstly, whole-genome protein sequences from all species were searched against themselves using BLASTP with an *E* < 1 × 10^−5^ (Song et al., 2014a). MCScanX was then used to detect collinear blocks with default parameter setting according to a previous report (Wang et al., 2012). Using output results of MCScanX, we extracted the *AP2/ERF* genes located in the collinear blocks. We checked gene conversion by referring to results of a previous analysis at the whole-genome scale (Chalhoub et al., 2014).

### Selective pressure and divergence time estimation

To estimate the divergence time between collinear *AP2/ERF* gene pairs, alignment of the protein sequences were performed using ClustalW2, and then were translated into CDS alignment. These softwares were implemented by BioPerl. We estimated the synonymous (Ks) and non-synonymous (Ka) nucleotide substitutions rate between the collinear genes using the method developed by Nei and Gojobori, implemented in KaKs_calculator (Wang et al., 2010). Divergence time was therefore inferred using the formula T = Ks/2R, where R is 1.5 × 10^−8^ synonymous substitutions per site per year (Koch et al., 2000). We applied likelihood ratio (LR) tests of positive selection based on the ML methods and codon substitution models. Based on previously reported methods (Mondragon-Palomino et al., 2002; Mondragon-Palomino and Gaut, 2005), we implemented Codeml from PAML package and analyzed groups I, VII, and AP2 family to infer ω, the ratio of the non-synonymous to synonymous distances (Yang, 1997; Yang et al., 2000). We employed complete deletion method when analysing alignments with gaps, and eliminated sequences that contained 40% of their length or more as InDels. We detected variation in ω among sites by employing a likelihood ratio test between M0 and M1, and M7 and M8 models.

### Expression pattern analysis

To analyze gene expression patterns, we used the Illumina RNA-seq data reported previously (Chalhoub et al., 2014), containing two tissues (root, leaf) of *B. napus*, and three replicates. The cluster was analyzed using Cluster3.0, and Reads Per Kilobase per Million mapped reads (RPKM) log2-transformed values. Heat maps were then constructed using TreeView (http://jtreeview.sourceforge.net/) for visualization of the clustering results. The boxplots of the gene expression were drawn by using R program, and the χ^2^-test was performed to detect significant differences between subgenomes or different tissues. The cases with expression change larger than two-fold, and the corresponding *P* < 0.05 were used to identify differentially expressed genes (DEGs).

## Results

### Identification of *AP2/ERF* genes in *B. napus*

**Figure 1:**
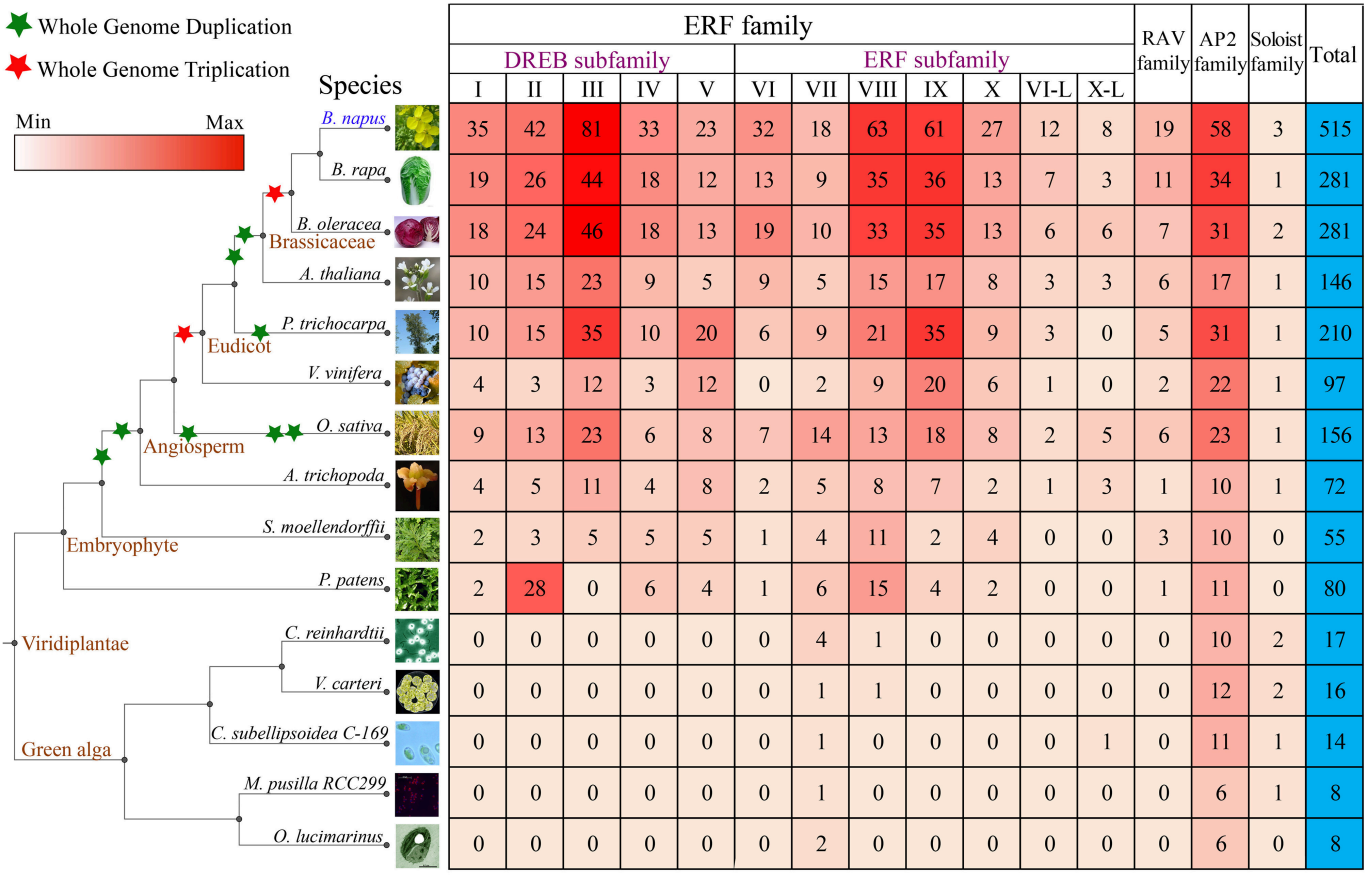
**The number and classification of *AP2/ERF* genes in *B. napus* and other 14 species used in this study**. Information regarding genome duplication or triplication was obtained from the Plant Genome Duplication Database (PGDD). The phenotypic picture for each species was obtained from the Phytozome.

In all considered plants, a total of 1956 *AP2/ERF* genes were identified (Figure 1). They were renamed according to plant names, chromosomal locations, and the family types, facilitating easy identification of their sources, distribution, and comparison (Table S1). In *B. napus*, 515 distinct *AP2/ERF* genes were identified, with 256 genes (BnaA###) from AA-subgenome and 258 (BnaC###) from CC-subgenome. Gene *BnaUnng01150D* failed to be assigned to any subgenomes, and was renamed as *BnaSERF*-*001*. According to conserved domain similarity to *Arabidopsis* genes, we classified *B. napus* AP2/ERF superfamily into four families, including ERF, AP2, RAV, and Soloist (Table S2). The ERF family contains DREB subfamily and ERF subfamily, and both of which can be further divided into several groups I-X, with groups I to V belonging to DERB subfamily, and VI to X belonging to ERF subfamily (Figure 1). There are 435 genes in ERF family, featuring a single AP2/ERF domain, and 58 genes in AP2 family, featuring tandem repetitive AP2/ERF motif (32) and high similarity to the *Arabidopsis* AP2 family. A single AP2 domain was reported in *Arabidopsis*, e.g., *AT2G39250, AT2G41710*, and *AT3G54990*, from AP2 family (Nakano et al., 2006). There were 19 genes in RAV family, featuring a single AP2/ERF DNA binding domain and a B3 domain. Three genes (*BnaCSoloist-001, BnaCSoloist-002*, and *BnaASoloist-001*) were identified to be Soloist ones, for sharing high similarity to *Arabidopsis* Soloist (*AT4G13040*) and divergent from the ERF family.

*B. napus* has the most *AP2/ERF* genes among the plants under consideration, about fewer than twice of those in *B. rapa* (281) or *B. oleracea* (281), showing likely about 9% gene losses in each subgenome after hybridization, over twice of that in *P. trichocarpa* (202), and over three times of those detected in *O. sativa* (156), *A. thaliana* (146), and other plants considered. The gene number of each ERF, AP2, RAV, and Soloist family in *B. napus* also exceeds those in other plants. There are much fewer *AP2/ERF* genes (< 20) in Green alga than higher plants. For example, *Ostreococcus lucimarinus* and *Micromonas pusilla RCC299* each have 8 *AP2/ERF* genes, indicating a vast expansion of the superfamily during the evolution of higher plants. Four gene families exist in nearly all the higher plants examined, excepting *S. moellendorffii* and *P. patens*, where the Soloist family was absent. No RAV or DREB gene was detected in the five Green alga species. Notably, we found that there were more *AP2* genes than other families in Green alga, different from that in higher plants.

### Chromosome distribution

Among 515 *AP2/ERF* genes detected in *B. napus*, 449 genes (87.18%) were anchored onto its 19 chromosomes (Figure S1). Further examination showed that 29 (43.94%) of the non-anchored 66 genes were derived from the AA-subgenome, and 36 (54.55%) genes were from the CC-subgenome of *B. napus*. *BnaSERF*-*001* (*BnaUnng01150D*) did not assign to neither AA- or CC-subgenomes. Chromosome BnaA03 has the highest number of *AP2/ERF* genes (33 or 7.35%), and BnaA04 has the lowest (11 or 2.45%). Interestingly, we found that most *AP2/ERF* genes were distributed on the ends of chromosomes. For example, the distance of *BnaAERF-175* from the chromosome ends (BnaA10) was only 22.31 Kb, and the distance of *BnaCERF-166* from the chromosome ends (BnaC09) was only 12.05 Kb. Nearly no gene was detected in the middle of chromosomes BnaA01, BnaA04, BnaA06, BnaA09, BnaA10, BnaC01, BnaC04, and BnaC09.

Many genes form merely small gene clusters on chromosomes. As to the criteria that two or more genes were located on the chromosome within 200 Kb (Figure S1), we found 42 and 32 clusters in the AA and CC-subgenomes, respectively. Most clusters contained only 2 or 3 genes, and the largest cluster contained 5 genes. In AA-subgenome, 26 clusters have 2 genes, involving 61.9% of genes in total; and 14 clusters have 3 genes, involving 33.3% of genes. In CC-subgenome, the percentage of 2 gene clusters was over 93.7% (30), and 3 gene clusters was only 6.3% (2), which was significantly different with the AA-subgenome. Interestingly, we identified 2 clusters, which contained 4, and 5 genes in AA-subgenome, while there was no cluster contained more than 3 genes in CC-subgenome. Furthermore, there were more genes located in clusters in AA-subgenome (103 genes) than in CC-subgenome (66 genes; Figure S2A, Table S3).

### Gene structure and conservative motif

Members of the same functional group mostly have similar intron/exon structure (Figure S3). Among 515 *AP2/ERF* genes, 309 (60.0%) of them have one exon and 445 (86.4%) genes had 1~3 exons, (Table S4), similar to GRAS and AP2/ERF superfamily in *B. rapa* (Song et al., 2013, 2014b). For instance, most genes from groups III, VI, VIII, and IX have only one exon, and the length of them are very similar. Overall, genes from the AP2 family have more exons and introns than those from families ERF and RAV (Figure S4). Some genes contained abnormally long introns, such as *BnaAERF*-*170* in group II, *BnaCERF*-*168* in group V, *BnaAERF*-*089* in group VII, and *BnaCAP2*-*015*, and *BnaCAP2*-*023* in group AP2. Some exceptional genes, such as *BnaARAV*-*006, BnaCERF*-*001*, and *BnaAERF*-*085*, contain 8 or more exons. In addition, all 58 *AP2* genes had 5~11 exons, which was significant different from ERF and RAV families (Figure S5). For example, the *AP2* genes *BnaAAP2*-*013, BnaAAP2*-*027*, and *BnaCAP2*-*012* had 11 exons and 10 introns. Furthermore, we found evidence that some newly duplicated genes in the superfamily have no introns, though their highly similar homologs have introns, evidencing their formation through retrotransposon activity.

### Subgenome-biased gene conversion

Using the gene conversion dataset previous reported (Chalhoub et al., 2014), we conducted the gene conversion analyses between AA- and CC-subgenome for *B. napus AP2/ERF* genes. A total of 68 gene pairs showed likely gene conversion (Figure S2A, Table S5). Among these gene pairs, 356 conversion sites were identified to occur with CC-subgenome as donor, while 267 conversion sites with AA-subgenome as donor, showing a significant bias between subgenomes (*P* = 3.6e-04).

To reveal the relations of gene conversion and physical clusters, we conducted comparative analyses of these two gene sets. Fifty-two (20.6%) *AP2/ERF* genes were shared between these two gene sets in the *B. napus* genome (Figure S2B). Among these genes, 29 (20.4%) genes located in the AA-subgenome, and 23 (20.7%) genes located CC-subgenome (Figures S2C,D).

### Homologous genes

**Figure 2:**
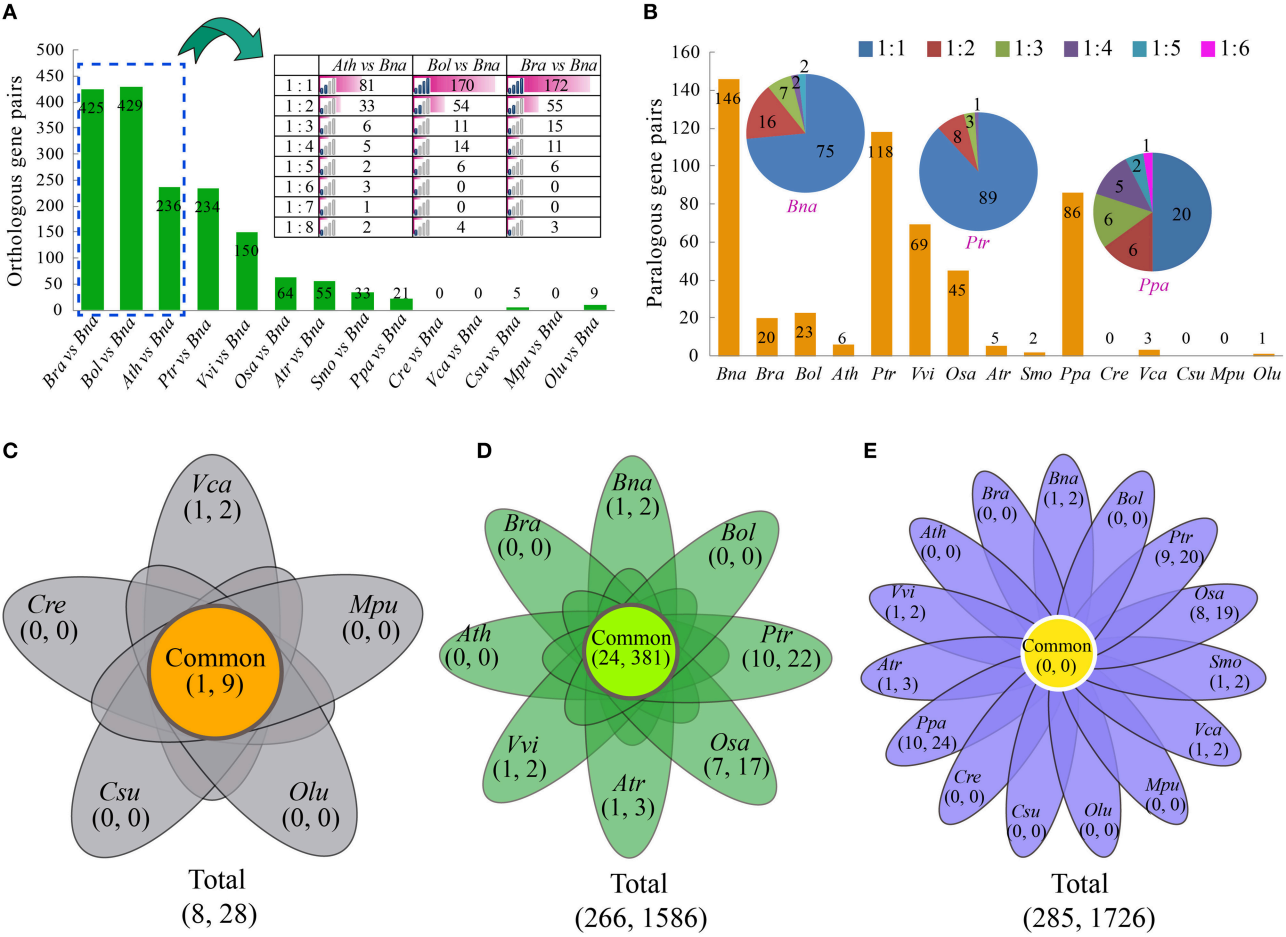
**The orthologous, paralogous, and cluster analyses of the *AP2/ERF* genes. (A)** The number of orthologous genes identified between *B. napus* and other 14 species. The ratio indicated that the one gene in species (*A. thaliana, B. oleracea*, or *B. rapa*) has the one or more orthologous genes with *B. napus*. **(B)** The number of paralogous genes identified in *B. napus* and other 14 species. The ratio indicated that the one gene has the one or more orthologous genes in each species. **(C)** The Venn diagram shows the number of common and specific clusters and *AP2/ERF* genes in five Green alga species. The first number in the brackets represents the number of cluster, and the second number represents the number of genes. **(D)** The Venn diagram shows the number of common and specific clusters and *AP2/ERF* genes in eight angiosperms species. **(E)** The Venn diagram shows the number of common and specific clusters and *AP2/ERF* genes in *B. napus* and other 14 species used in this study. The abbreviations represent the species as follows: *Bna, B. napus; Bra, B. rapa; Bol, B. oleracea; Ath, A. thaliana; Ptr, P. trichocarpa; Vvi, V. vinifera; Osa, O. sativa; Atr, A. trichopoda; Smo, S. moellendorffii; Ppa, P. patens; Cre, C. reinhardtii; Vca, V. carteri; Csu, C. subellipsoidea C-169; Mpu, M. pusilla RCC299; Olu, O. lucimarinus*.

Not surprisingly, among all 14 species, *B. oleracea* and *B. rapa* have the most orthologous gene pairs with *B. napus* than other species (Figure 2A, Table S6). The number of orthologous gene pairs between *B. oleracea* and *B. napus* was 429, very close to that between *B. rapa* and *B. napus* (425), about nearly twice of that between *A. thaliana* and *B. napus* (236). There are only 5 and 9 orthologous *AP2/ERF* gene pairs detected between two green algas, *Coccomyxa subellipsoidea C-169* and *O. lucimarinus*, and *B. napus*, respectively, (Figure 2A). We found that each *Arabidopsis AP2/ERF* gene had one to eight *B. napus* orthologous genes, demonstrating that some *AP2/ERF* genes in *B. rapa* and *B. oleracea* were subjected to copy number increase likely due to the recursive polyploidizations.

To check how different duplication events have contributed to the expansion of the superfamily, we identified collinear paralogous genes (Figure 2B, Table S7). More paralogous gene pairs were observed in *B. napus* (146)*, P. trichocarpa* (118), and *P. patens* (86) than in the other species. However, there were only 20, 23, and 6 paralogous *AP2/ERF* gene pairs in *B. rapa, B. oleracea*, and *A. thaliana*, respectively, (Figure S6). This phenomenon indicated that several *AP2/ERF* genes might be lost or subjected to fast sequence divergence after polyploidization. In green alga, there were much fewer paralogous gene pairs than in the higher plants. It might be due to the fact that the genomes of green alga did not undergo whole-genome duplication as most of the higher plants did.

Furthermore, we identified *AP2/ERF* genes clusters using MCL algorism according to the previous report (Xu et al., 2013; Song et al., 2015). Firstly, we checked gene clusters among the 5 green alga (Figure 2C, Table S8). A total of 8 clusters were detected, containing 28 *AP2/ERF* genes. Secondly, we checked gene clusters among 8 angiosperms (Figure 2D, Table S9). Totally, 266 clusters were detected, which contained 1586 *AP2/ERF* genes. A total of 24 clusters contained 381 genes, which were shared by all the 8 angiosperms. Last but not the least, we checked gene clusters among *B. napus* and other 14 species (Figure 2E, Table S10). A total of 285 clusters were detected, containing 1726 *AP2/ERF* genes. However, there was no cluster shared by all these species.

### Causes of gene expansion

**Figure 3:**
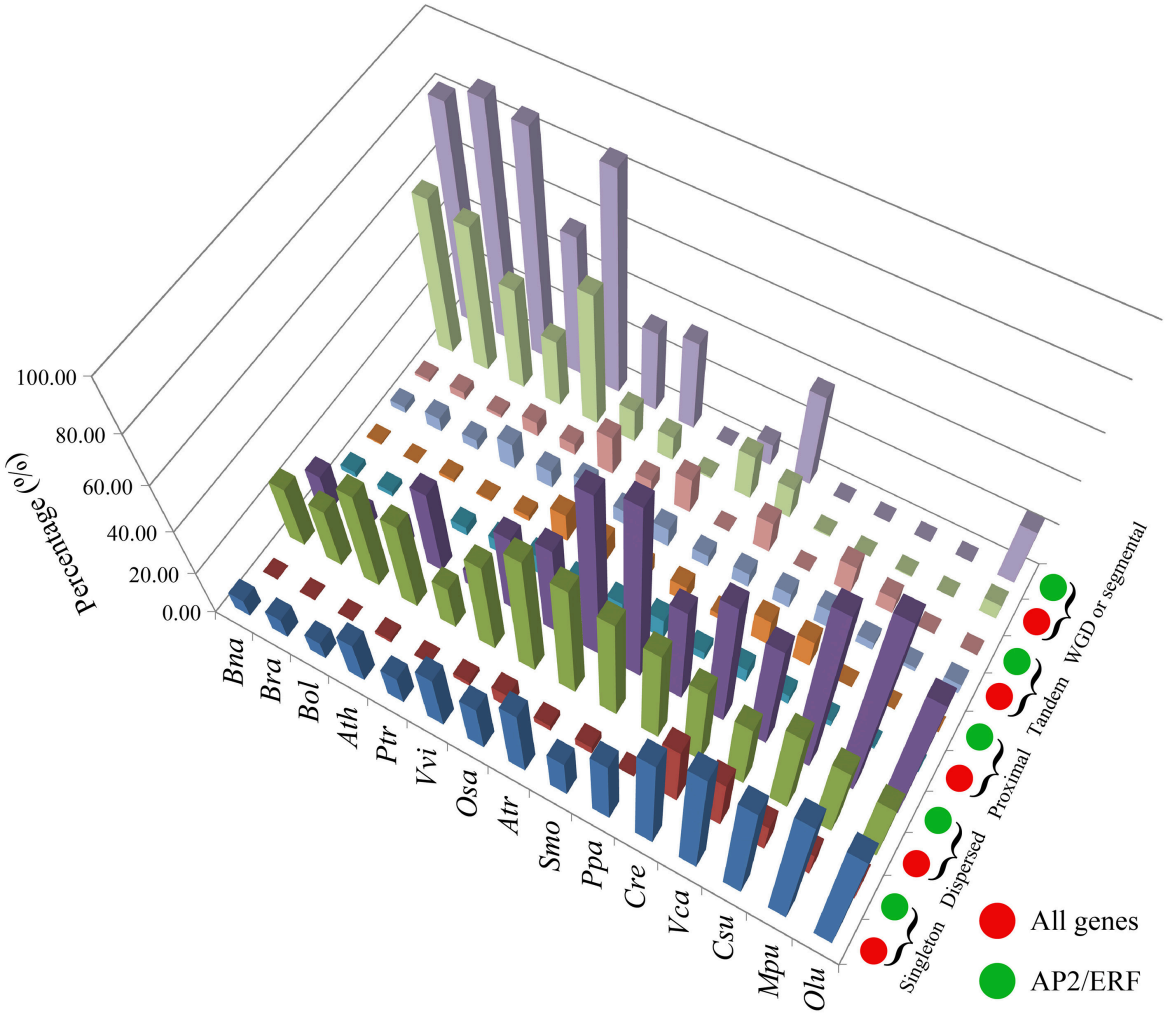
**The three-dimensional histogram of the duplicated type number for *AP2/ERF* genes and all genes in the whole genome of *B. napus* and other 14 species**.

Genomic duplication may have contributed to the expansion of the superfamily. We examined 5 types of gene duplications: singleton, dispersed, proximal, tandem, and whole-genome duplication/triplication (WGD/T) or segmental duplication (Figure 3, Table 1). For the *AP2/ERF* genes, WGD/T or segmental duplication contributed the most to the expansion of this superfamily in *B. napus* (423, 82.14%), *B. rapa* (249, 88.61%), *B. oleracea* (242, 86.12%), *A. thaliana* (81, 55.48%), and *P. trichocarpa* (179, 85.24%; Table S11). The percentage of *AP2/ERF* genes that had undergone WGD/T or segmental duplication was greater than the average percentage of the whole-genome level (Table S11).

**Table 1:** **The number of *AP2/ERF* genes for each duplicated type in *B. napus* and the other 14 species**.

**Species**	**Singleton**		**Dispersed**		**Proximal**		**Tandem**		**WGD or segmental**		**Total**	
	**Genome**	**AP2/ERF**	**Genome**	**AP2/ERF**	**Genome**	**AP2/ERF**	**Genome**	**AP2/ERF**	**Genome**	**AP2/ERF**	**Genome**	**AP2/ERF**
*Bna*	7768	0	26,907	83	2428	3	2708	6	61,229	423	10,1040	515
*Bra*	3666	1	10,622	22	873	0	2369	9	23,489	249	41,019	281
*Bol*	4807	0	25,232	31	2515	4	2523	4	24,148	242	59,225	281
*Ath*	5155	2	10,670	53	1046	1	3026	9	7519	81	27,416	146
*Ptr*	5427	1	8152	15	2538	5	3159	10	22,059	179	41,335	210
*Vvi*	6613	2	10,819	35	2267	12	3032	16	3615	32	26,346	97
*Osa*	12,368	11	30,411	64	3761	15	3533	9	5728	57	55,801	156
*Atr*	8608	2	13,985	54	1849	5	2195	11	209	0	26,846	72
*Smo*	4164	2	10,621	44	2094	4	1143	0	4263	5	22,285	55
*Ppa*	10,546	0	14,636	36	1238	2	2083	11	4423	31	32,926	80
*Cre*	8478	5	6793	10	1139	2	1218	0	113	0	17,741	17
*Vca*	8467	4	4868	8	673	2	1277	2	0	0	15,285	16
*Csu*	5027	2	3943	11	263	0	352	1	44	0	9629	14
*Mpu*	6178	1	3639	7	105	0	181	0	0	0	10,103	8
*Olu*	4536	1	2317	5	67	0	354	0	522	2	7796	8

*The abbreviations represent the species as follows: Bna, B. napus; Bra, B. rapa; Bol, B. oleracea; Ath, A. thaliana; Ptr, P. trichocarpa; Vvi, V. vinifera; Osa, O. sativa; Atr, A. trichopoda; Smo, S. moellendorffii; Ppa, P. patens; Cre, C. reinhardtii; Vca, V. carteri; Csu, C. subellipsoidea C-169; Mpu, M. pusilla RCC299; Olu, O. lucimarinus*.

**Figure 4:**
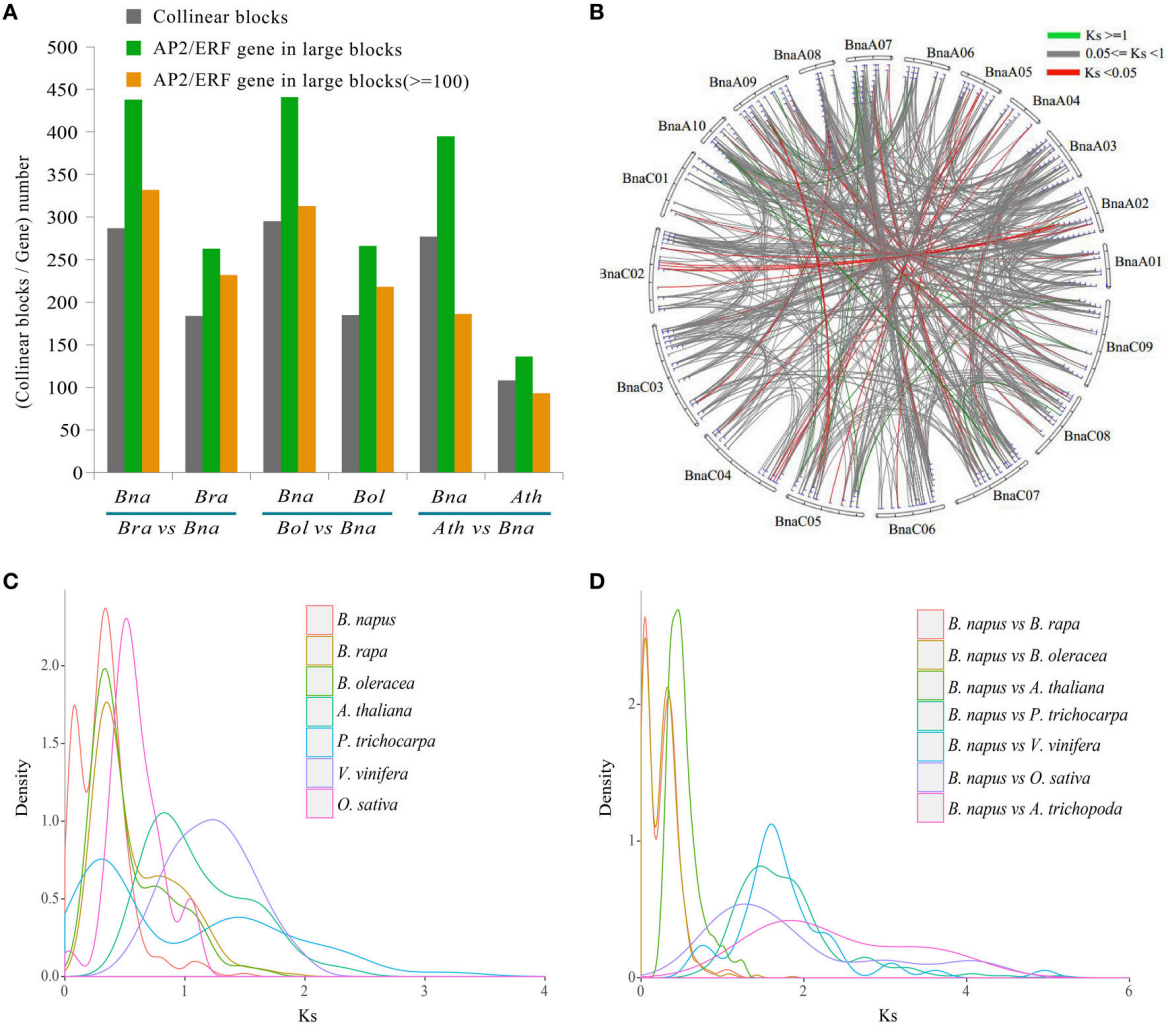
**The identification of collinearity block and Ks analyses between *B. napus* and other species. (A)** The summary of collinearity blocks and *AP2/ERF* genes number between *B. napus* and other related species. **(B)** The circle plot of Ks values for all syntenic superfamily *AP2/ERF* genes in *B. napus*. **(C)** The density of Ks values for syntenic *AP2/ERF* gene pairs of each species. **(D)** The density of Ks values for syntenic *AP2/ERF* gene pairs between *B. napus* and other related species.

By checking gene collinearity within a genome, we found that more than 60% *AP2/ERF* genes in four *Brassicaceae* species were located in large collinear blocks with >100 collinear genes (Figure 4A, Table 2, Figures S7–S9), showing their duplication during polyploidization. In *B. napus*, we identified 586 collinear *AP2/ERF* gene pairs, (Table 3, Table S12), two times more than those in *B. rapa* (273) and *B. oleracea* (252). Among these collinear gene pairs, 415 *AP2/ERF* genes were detected in *B. napus*, followed by *B. rapa* (243), *B. oleracea* (238), and *P. trichocarpa* (212). In many other species, there were fewer than 100 genes in collinearity. Especially, in *A. trichopoda* and 5 green alga, no collinear *AP2/ERF* gene pair was found. Though non-*Brassicaceae* plants have much shorter intragenomic collinear blocks, we found that the percentage of *AP2/ERF* genes located in the collinear blocks was larger than the genome-wide average, showing that polyploidization contributed to the expansion of the superfamily.

**Table 2:** **The collinearity blocks identified in the whole genome and the *AP2/ERF* genes between *B. napus* and the other species**.

**Species**	**Total collinear blocks**	**Gene number in collinear blocks**	**Total gene**	**Percentage (%)**	**Collinear blocks contained AP2/ERF**	**AP2/ERF gene in collinear blocks**	**Total AP2/ERF**	**Percentage (%)**	**AP2/ERF gene number (≥100 collinear blocks)**
*Bna*	2468	60,293	10,1040	59.67	287	438	515	85.05	332
*Bra*		35,036	41,019	85.41	184	263	281	93.59	232
*Bna*	2881	64,337	10,1040	63.67	295	441	515	85.63	313
*Bol*		39,477	59,225	66.66	185	266	281	94.66	218
*Bna*	1749	49,779	10,1040	49.27	277	395	515	76.70	186
*Ath*		19,774	27,416	72.13	108	136	145	93.79	93
*Bna*	2441	17,050	10,1040	16.87	125	133	515	25.83	0
*Ptr*		11,406	41,335	27.59	85	87	210	41.43	0
*Bna*	1233	12,086	10,1040	11.96	59	62	515	12.04	0
*Vvi*		5933	26,346	22.52	25	25	97	25.77	0
*Bna*	55	355	10,1040	0.35	5	6	515	1.17	0
*Osa*		304	55,801	0.54	6	8	156	5.13	0
*Bna*	119	829	10,1040	0.82	6	6	515	1.17	0
*Atr*		579	26,846	2.16	4	4	72	5.56	0
*Bna*	4	39	10,1040	0.04	0	0	515	0.00	0
*Smo*		36	22,285	0.16	0	0	55	0.00	0

**Table 3:** **The statistics of Ka/Ks values for *AP2/ERF* genes belonged to the syntenic gene pairs in each species**.

**Species**	**Syntenic gene pairs**	***AP2/ERF* gene**	**Syntenic gene pairs**		
			**Ka/Ks > 1**	**0.1 ≤ Ka/Ks ≤ 1**	**Ka/Ks < 0.1**
*Bna*	586	415	6	566	14
*Bra*	273	243	1	270	2
*Bol*	252	238	8	242	1
*Ath*	52	78	0	51	1
*Ptr*	155	212	1	152	2
*Vvi*	20	30	0	20	0
*Osa*	31	56	4	27	0
*Atr*	0	0	0	0	0
*Smo*	2	4	0	2	0
*Ppa*	7	12	0	4	3
*Cre*	0	0	0	0	0
*Vca*	0	0	0	0	0
*Csu*	0	0	0	0	0
*Mpu*	0	0	0	0	0
*Olu*	0	0	0	0	0

We identified collinear gene pairs between *B. napus* and other plants (Table S13). The collinear *AP2/ERF* gene pairs were only detected between *B. napus* and 7 angiosperms. A total of 837 collinear *AP2/ERF* gene pairs were detected between *B. napus* and *B. rapa*, followed by *B. oleracea* (808), *A. thaliana* (442), and *P. trichocarpa* (177).

Sequence divergence analysis supported the polyploidizations have contributed to expansion of the superfamily. Among 586 collinear *AP2/ERF* gene pairs of *B. napus*, Ks values were from 0.0057 to 1.5340, and the corresponding divergence time from 0.19 to 51.13 MYA (Figure 4B, Table S12). To more directly demonstrate the divergence of collinear *AP2/ERF* genes, we plotted Ks distribution for each species and between each two of them (Figures 4C,D), and found two obviously peaks in *B. napus*, and one peak in each of the other species (Figure 4C, Figure S10). For *B. napus*, the first peak might be formed due to the hybridization between *B. rapa* and *B. oleracea*, and the second peak, shared by *B. rapa* and *B. oleracea*, might be formed due to the *Brassica* triplication events 5~9 MYA.

### Phylogenetic and evolutionary analysis

**Figure 5:**
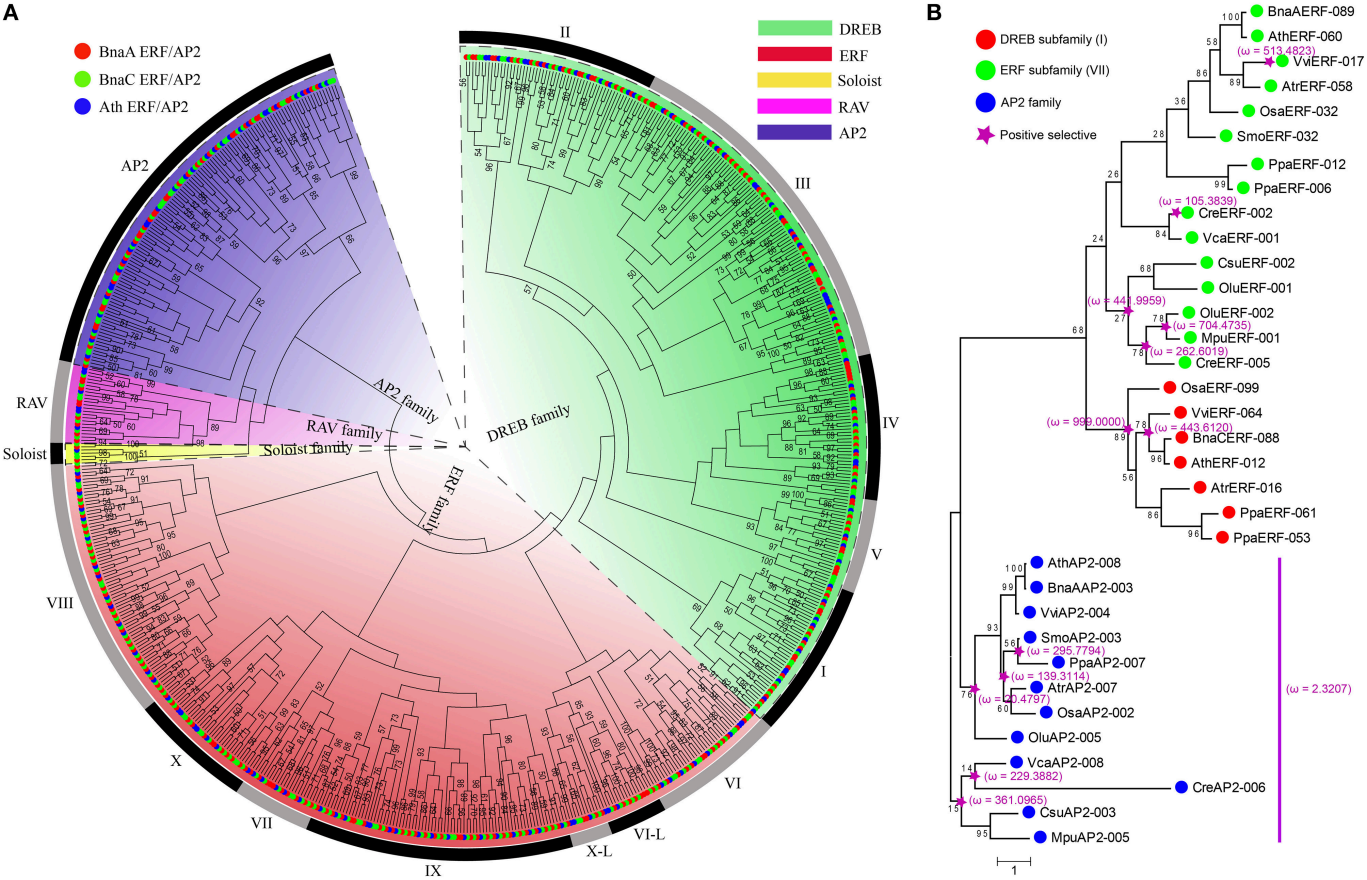
**The phylogenetic relationship and positive selection analyses of AP2/ERF superfamily. (A)** Phylogenetic tree constructed using neighbor-joining method by MEGA6, using the AP2 domain sequences of *AP2/ERF* genes in *B. napus* and *Arabidopsis*. The numbers are bootstrap values based on 1000 iterations. Only bootstrap values with >50% supports are indicated. **(B)** The positive selection analyses among DREB subfamily (I), ERF subfamily (VII), and AP2 families in representative species. The ω on the clades is dn/ds value under M8 of codeml, which represent the results of positive selection analyses.

As to the constructed phylogenetic tree of AP2/ERF superfamily, genes can be divided into 4 distinct clades (Figure 5A), corresponding to the ERF (ERF subfamily and DREB subfamily), AP2, RAV, and Soloist families, respectively, congruent with previous studies (Sakuma et al., 2002; Nakano et al., 2006). The DREB and ERF family can be further divided into 10 groups (groups I to V for DREB, and VI to X for ERF family).

We explored the origination and evolution of this superfamily in higher and lower plants. Several groups were not detected in the lower plants. For example, no gene was found in DREB groups I to V, and RAV family in five Green alga species (Figure 1, Figure S11). This supports a hypothesis that DREB family genes in land plants acquired these groups from other genes during the very early stage of their origination. To evaluate this hypothesis, we managed to reconstruct the phylogenetic tree. As to ERF subfamily, genes from VII and VIII were found in both green alga and land plants, while genes from VI, IX, X, and VI-L groups found only in land plants but not in alga. Notably, the group VII genes existed in five green alga species. These facts support that the ERF subfamily may have been firstly originated and expanded from the group VII. The DREB subfamily and RAV family were found in *P. patens* and other land plants, but not in green alga. The phylogeny analysis implied that the expansion of DREB subfamily was the most likely from its group I, after acquiring an AP2 domain (Figure 5A, Figure S11). The RAV family might be originated from the AP2 family after loss one AP2 domain and acquired a new B3 domain from an unknown source. Therefore, we speculate that the AP2 domain in higher plants for groups I to VI, IX or RAV family was acquired from other groups through gene-gene merge, which is a normal avenue to produce new genes.

### Frequent drive of positive selection

Strong positive selection was observed on the major nodes leading to origination and divergence of higher plants. To uncover whether and when natural selection had acted on the evolution of AP2/ERF superfamily, we performed selection pressure analyses in group I (DREB subfamily), group VII (ERF subfamily), and AP2 family, respectively, (Figure 5B). A whole-scale analysis of all genes and families was not done in that too many genes were computationally infeasible for software PAML. In group I, significantly more non-synonymous than synonymous substitutions was detected, showing strong positive selection before the divergence of higher plants. In addition, significantly strong positive selection (ω = 443.6120) was detected before the divergence of the examined eudicot plants. In group VII, interestingly, more positive selected nodes were found during the divergence of five green alga than the higher plants, in which, actually, only 1 examined gene (*VviERF-017*) was inferred to be positively selected (ω = 513.4823). This seemingly suggested that group VII genes have been subjected to much selective pressure during the evolution of alga. As to the AP2 family, which was inferred as to be the likely ancestor node of the superfamily, was also subjected to positive selection during its further divergence with higher plants. Besides, positive selection (ω = 2.3207) was also observed on the branch before the split of the higher plants and green alga. For the higher plants, the positive selection was detected (ω = 139.3114) on the branches leading to the divergence of *SmoAP2-003, PpaAP2-007, AtrAP2-007*, and *OsaAP2-002*, but not on the branches leading to *AthAP2-008, BnaAAP2-003*, and *VviAP2-004*.

### AP2 family evolution mechanism in plants

**Figure 6:**
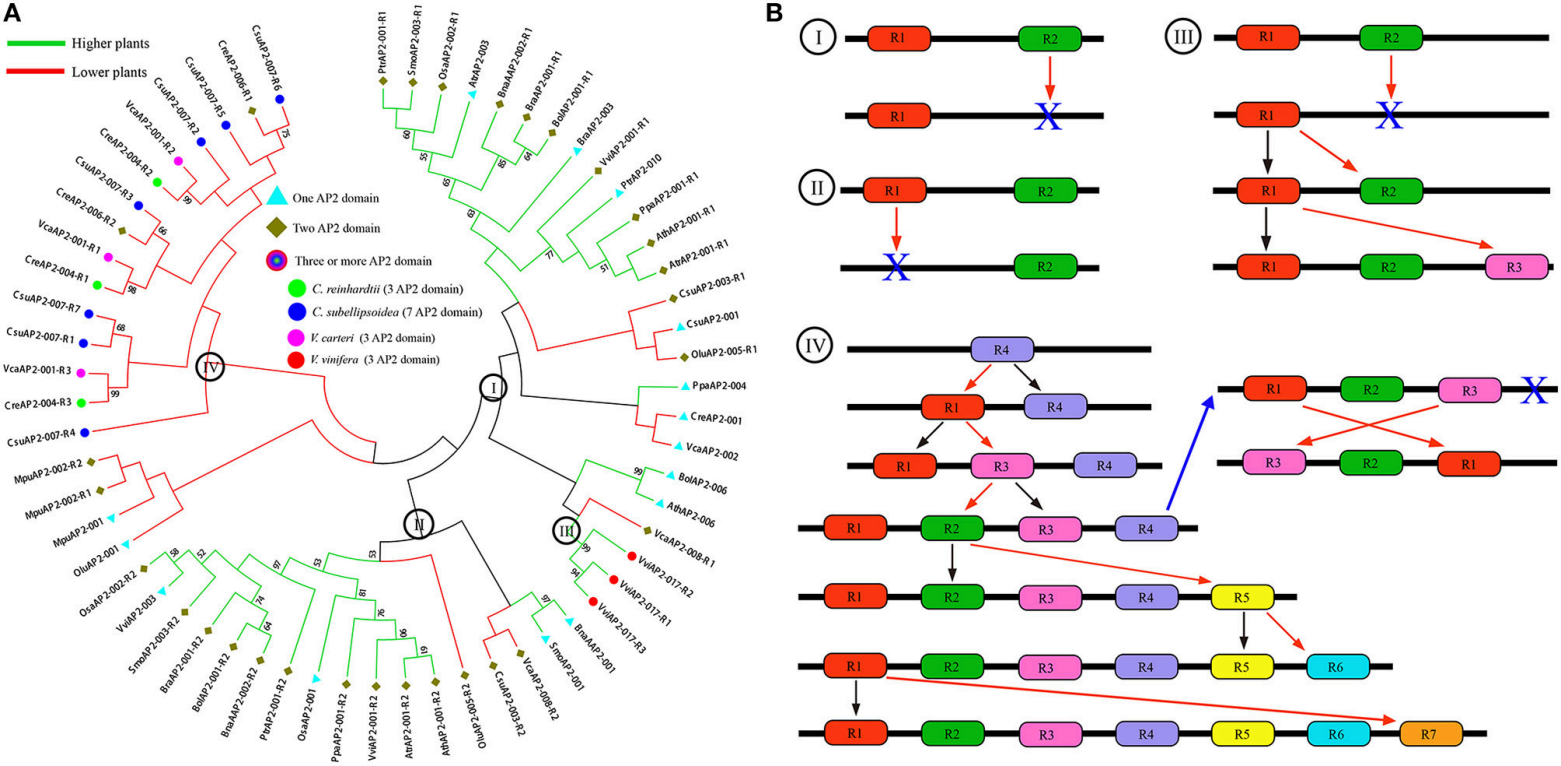
**The phylogenetic relationship and evolutionary trajectories of AP2 family in plants. (A)** Phylogenetic tree constructed using neighbor-joining method by MEGA6, using AP2 domain sequences of AP2 family genes in higher (Green branches) and lower (Red branches) plants. **(B)** The major evolutionary trajectories of AP2 family. The R1 to R7 represent the AP2 domains from 5′ to 3′ of the AP2 family genes. The blue X indicated the AP2 domain was lost in the genes.

Among the AP2/ERF superfamily, most family genes had one AP2 domain except of the AP2 family genes, which had one or more, and mostly two, AP2 domains. For example, three genes (*VviAP2-017, VcaAP2-001*, and *CreAP2-004*) had three AP2 domains. Even more, seven AP2 domains were detected in the gene *CsuAP2-007* of *C. subellipsoidea C-169*. To uncover the evolution mechanism of AP2 domain loss or duplication in plants, we constructed the phylogenetic tree using the AP2 domain of the representative AP2 family in each species examined (Figure 6A). We found, for genes that have two domains, that the two domains (denoted as R1 and R2 in order on genes), could often be divided into two branches in the higher plants, and most R1 domains form a group, and R2 domains form another. At the meanwhile, domains from single-domain genes appeared in an intervening manner with the R1 or R2 domains. For example, three *Brassica* two-domain genes *BnaAAP2-002, BraAP2-001*, and *BolAP2-001* are grouped with a single-domain gene *BraAP2-003*.

Here, as to copy number variation of AP2 domains, we found two major evolutionary trajectories of AP2 family (Figure 6B). (i) Alternative domain loss; this occurred in both lower and higher plants, with some lost R1 and the others R2 (Figure 6B-model I and II). (ii) Duplication, loss and likely conversion; this occurred to the grape gene *VviAP2-017* and alga gene *MpuAP2-002*. The grape gene has three domains closely grouped together with the first domain (R1) from a two-domain gene. This can be explained by recursive duplication of the R1 domain with R2 domain lost at some stage, or alternatively explained by conversion of R2 domain to R1 (Figure 6B-III). The alga gene *CsuAP2-007* has 7 domains, each of which is often shared with other alga species. Based on the phylogenetic trees, we inferred these alga species had at least 4 domains in their common ancestor, and likely R4 in *CsuAP2-007* was the most ancient one. A series of domain duplication, and likely conversion between domains, may have contributed to the gene's evolution (Figure 6B-IV). Interestingly, we found that R1 and R3 domains of *C. reinhardtii* and *V. carteri* genes likely exchanged their locations, as compared to *C. subellipsoidea C-169*. Besides, they all lost the R4 domain, and these changes might have occurred before their split.

### Gene expression profiling

**Figure 7:**
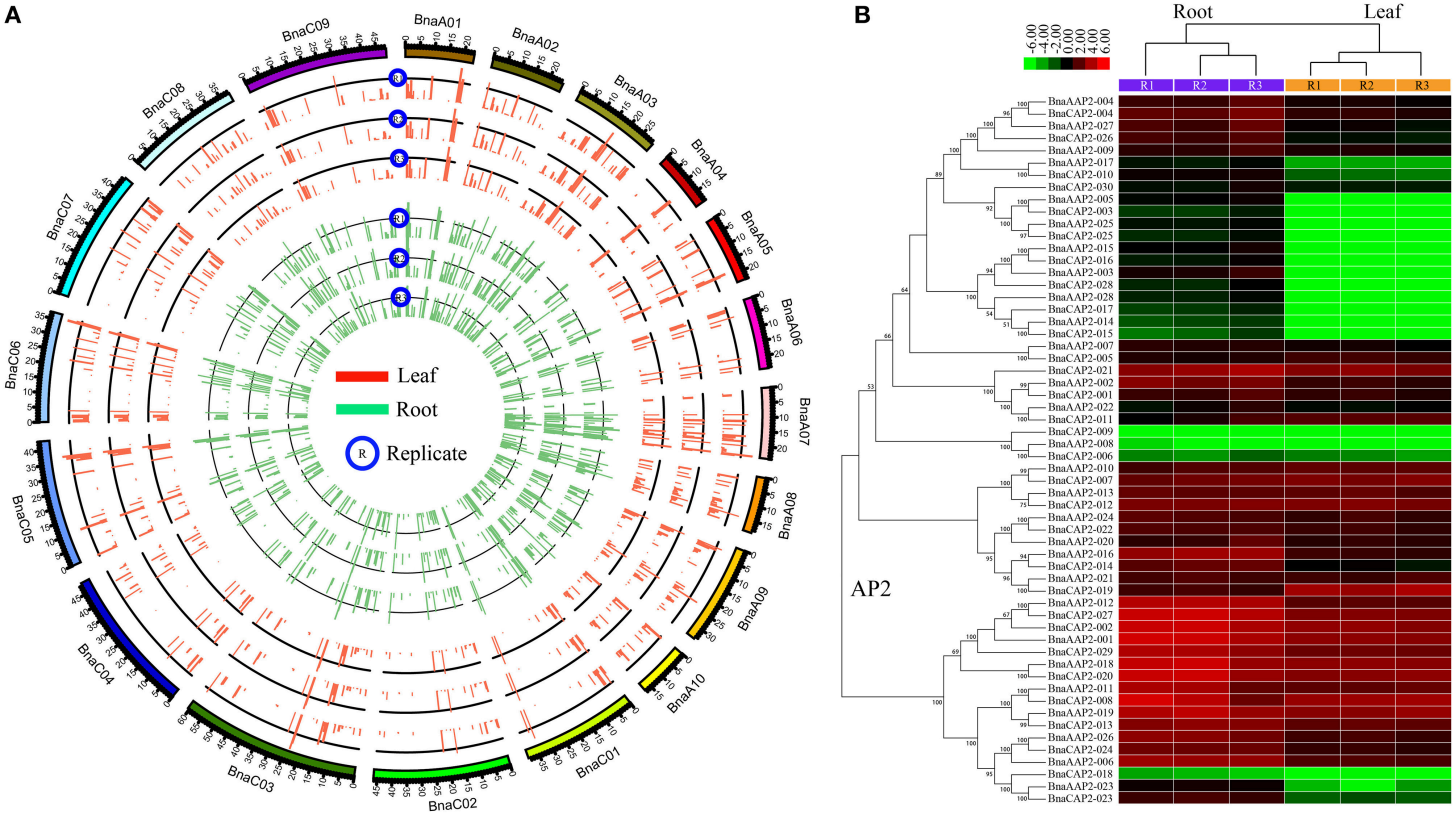
**The expression analyses for *AP2/ERF* genes in *B. napus***. The expression values were calculated by RPKM (Reads Per Kilobase per Million mapped reads). **(A)** The circle plot of the expression values for all *AP2/ERF* genes in *B. napus*. The red and green lines indicated the *AP2/ERF* gene expression for three replicates in leaf and root, respectively. **(B)** The Heat map representation and hierarchical clustering of *AP2* genes in root and leaf. The expression values were log2 transformed.

We analyzed expression level of *AP2/ERF* genes in leaf and root of *B. napus*. This dataset contained three replicates for leaf and root, and the expression level was calculated and normalized to RPKM (Table S14). Our analyses showed a good correlation among three replicates (Figure 7A). Totally, 504 *AP2/ERF* genes were expressed in at least one tissue, and 11 genes were not expressed in both. In root, 325 genes were expressed with the RPKM values larger than 1.0. The RPKM value of three genes (*BnaAERF-141, BnaCERF-210, BnaAERF-054*) was more than 100. In leaf, 206 genes were expressed with the RPKM values larger than 1.0, and only 1 gene (*BnaCERF-049*) with the RPKM larger than 50. A total of 185 genes with RPKM >1 were detected in both tissues. Interestingly, the RPKM value of *BnaCERF-049* were achieved 95.007 in root, and 50.099 in leaf, demonstrating that it may be important in *B. napus*.

We compared the expression patterns of genes from the same family or group (Figure S12). The results showed that the expression pattern of several genes was different with other genes in the same group. For example, we found several genes were down-regulated in leaf, while others were up-regulated in leaf for AP2 family (Figure 7B). Although the expression pattern was divergent in the whole gene family, the expression pattern of genes from the same clade in phylogenetic tree was similar, showing relative consistence between evolutionary closeness and functional similarity.

**Figure 8:**
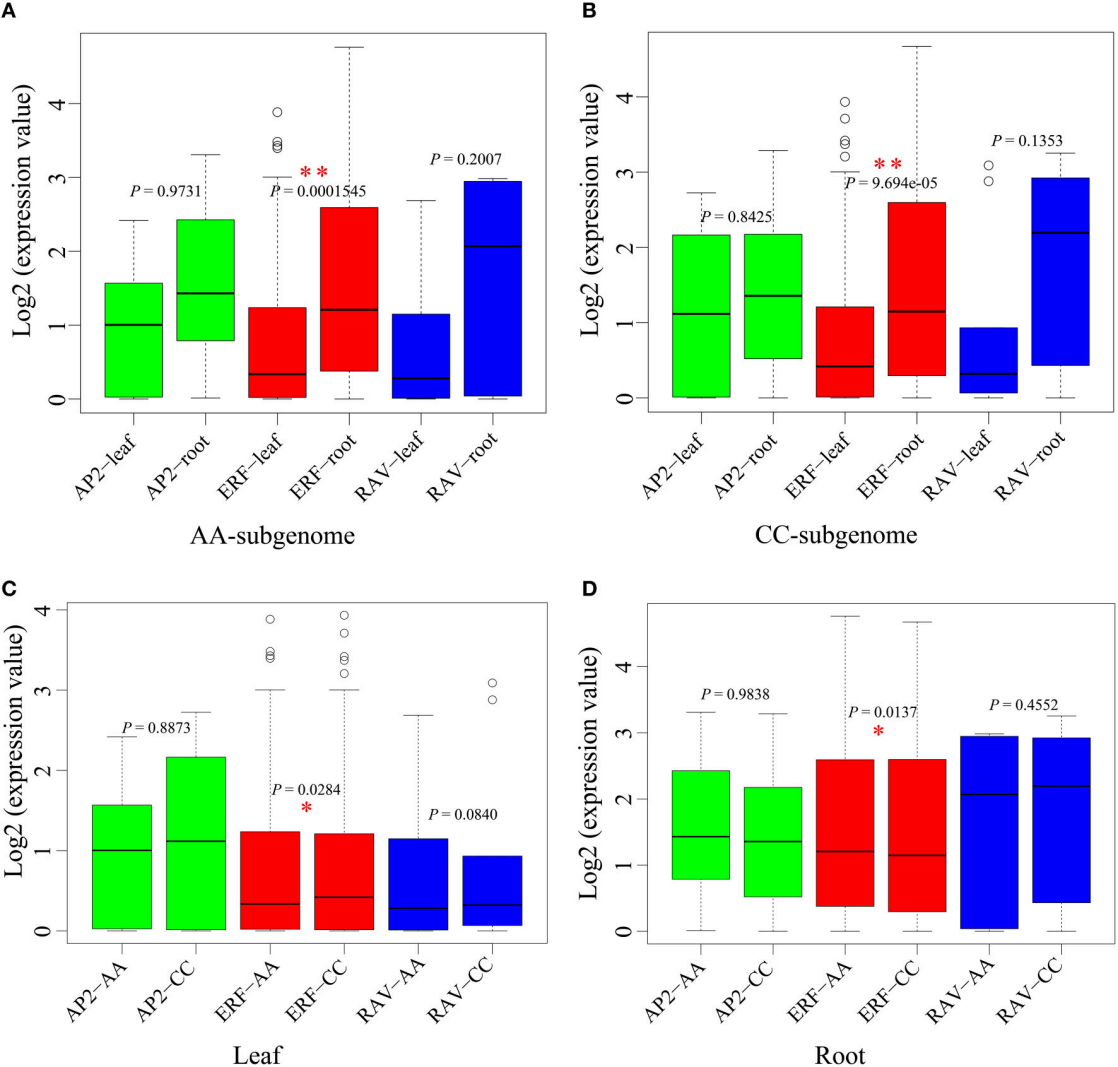
**The expression comparative analyses for *AP2/ERF* genes in *B. napus*. (A)** The comparative of *AP2/ERF* gene expression between leaf and root in AA-subgenome. **(B)** The comparative of *AP2/ERF* gene expression between leaf and root in CC-subgenome. **(C)** The comparative of *AP2/ERF* gene expression between AA-subgenome and CC-subgenome in leaf. **(D)** The comparative of *AP2/ERF* gene expression between AA-subgenome and CC-subgenome in root. ^*^P < 0.05; ^**^P < 0.01.

We analyzed the difference of the each *AP2/ERF* genes expression between tissues (Figures 8A,B). Grossly, we found that the average gene expression in root was higher than that in leaf (χ^2^-test, *P* = 8.525e-06), showing obvious tissue specificity. For ERF family, the average gene expression was significant different between leaf and root in the whole-genome scale, and in each of AA-subgenome (χ^2^-test, *P* = 1.545e-04) and CC-subgenome (χ^2^-test, *P* = 9.694e-05). For AP2 and RAV family, no significant difference was found between root and leaf.

In addition, we checked whether there is any subgenome bias with genes expression (Figures 8C,D). In leaf, the average expression level of genes from ERF family in CC-subgenome was significantly higher than those in AA-subgenome (χ^2^-test, *P* = 0.0284), while no significant difference was found between subgenomes for AP2 and RAV families. In root, the average expression level of genes from ERF family in the CC-subgenome was significantly lower than those in AA-subgenome (χ^2^-test, *P* = 0.0137), while no significant difference was observed between subgenomes for AP2 and RAV families.

## Discussion

### Identification of the AP2/ERF superfamily

*AP2/ERF* genes were viable candidates to improve abiotic and biotic stress in plants, responding to variable stresses, such as heat (*AtDREB1A, AtDREB2C, ZmDREB2A;* Qin et al., 2007; Hong et al., 2009; Chen et al., 2012), low-temperature (*AtCBF1, TaCBF*; Jaglo-Ottosen et al., 1998; Soltesz et al., 2013), drought (*OsDREB1, ERF6*; Ito et al., 2006; Dubois et al., 2013), osmotic tolerance (*FaDREB2, MaERF*-*B2*; Li et al., 2011; Shang et al., 2014), and cell differentiation (*WIND1, EBE, MFS1*; Iwase et al., 2011; Mehrnia et al., 2013; Ren et al., 2013).

Here, we identified and characterized the genomic structural, compositional, and expressional features of *AP2/ERF* genes in *B. napus*. Moreover, by performing evolutionary and phylogenetic analysis, we inferred their origination, formation, and evolution during the origination and evolution of land plants. These efforts can serve as a first step in comprehensive functional characterization of *AP2/ERF* genes by reverse genetic approaches and molecular genetics research.

### Exploring *AP2/ERF* genes function in *B. napus*

The functions of most *AP2/ERF* genes have been well characterized in *Arabidopsis*. A comparison of sequence homologs between *B. napus* and *Arabidopsis*, might aid in understanding the function of these *AP2/ERF* genes in *B. napus*. We checking genes within the same taxonomic group on the phylogenetic tree, which could have similar functions. We identified 4 *AP2/ERF* genes (*BnaCERF-139, BnaAERF-209, BnaCERF-033, BnaAERF-047*) in *B. napus*, which clustered together with the *AtCBF1*-*3* (Figure S13A), functionally relating to cold tolerance (Gilmour et al., 1998; Lata and Prasad, 2011; Table S15). For *AtCBF4* gene, functionally relating to drought and ABA response (Haake et al., 2002), two homologous genes were identified in *B. napus* (Figure S13A; Table S15). Similarly, 4, 5, and 2 homologous genes in *B. napus* were clustered, respectively, together with *AtDREB2A*-*2C* (Figure S13B), functionally relating to drought, salt, heat, or cold tolerance (Table S15; Sakuma et al., 2006; Lim et al., 2007; Djafi et al., 2013).

### Subgenome bias in *B. napus*

As a very young neo-tetraploid, *B. napus* may have formed for only ~7500 years (Chalhoub et al., 2014), by hybridizing the genomes of *B. rapa* and *B. oleracea*. This provided a precious opportunity to understand how genes or gene families were affected in young polyploids. Here, we found different gene groups from the AP2/ERF superfamily, were much preserved after the formation of the tetraploid, showing that the genome structure has been much stable, i.e., a very low rate of gene loss, which was often proposed to be wide-spread during the early stage of a neo-polyploid, contributing to fast diversification of new plants (Jiao et al., 2011; Paterson et al., 2012; Liu et al., 2014; Woodhouse et al., 2014). Actually, in some artificial/synthetic tetraploids, chromosomal DNA and gene loss rates can be 15% during the first generations (Ozkan et al., 2001, 2002). Therefore, the finding here shows that an appreciable span of genome stability should occur for polyploids. Some may have very instable genomes, but others may have very stable ones. The *B. napus* genome should be like the latter.

A very stable genome in whole-scale may permit considerable subgenome interaction. It has been reported that illegititmate recombination may occur between subgenomes, leading to crossing-over or gene conversion (Wang et al., 2009b; Wang and Paterson, 2011; Guo et al., 2014). Gene conversion transfers genetic information in a unidirectional manner between genes. Divergence between subgenomes would decrease illegitimate recombination sharply (Peters et al., 2009). Especially, chromosomal rearrangements may be a critical restricting factor (Wang et al., 2009b). The two subgenomes of *B. napus* are much similar in chromosome numbers and compositions, permitting appreciable illegitimate recombination to occur. Homoeologous exchanges were inferred between the two subgenomes, and conversion could explain 86% differences between subgenomes (Chalhoub et al., 2014). Significant bias was observed between subgenomes that nearly 1.3 times more conversions occurred from the subgenome AA to subgenome CC than the other direction. Here, we found that 68 genes from AP2/ERF superfamily was much affected by gene conversion, and the conversion events occurred biasedly from subgenome AA to CC, showing a similar trend as to the whole-genome finding.

Subgenome bias was also observed for gene expression. In leaves, the ERF family genes from subgenome CC significantly higher expressed than those from subgenome AA, whereas in roots, an opposite finding was revealed. This revealed a tissue-related gene expression bias between two subgenomes. In contrast, other genes from the superfamily did not show this kind of bias. A biased gene conversion and gene profiling showed the relative dominance between two subgenomes. Previously, maize was shown to have two subgenomes (Schnable et al., 2011), which merged together ~26 millions of years ago to produce the ancestral tetraploid (Wang et al., 2015b). However, one subgenome shows dominance over the other one in gene retention and gene expression (Schnable et al., 2011). Here, in a very young neo-tetraploid, *B. napus*, though two subgenomes may have preserved much of their ancestral genes, we observed biased gene expression and gene conversion as to a gene superfamily. This shows that subgenome bias may be popular effect between hybridized genomes in polyploids. As to the cause of subgenome bias or dominance, there have been hypothesis that differential genomic methylation might have played a role (Woodhouse et al., 2010). However, after thousands or millions of years, methylation pattern might have not been able to transfer through so many generations and have changed considerably, therefore there has been little evidence to support such a hypothesis.

### Origination and expansion

As to our phylogenetic analysis, the superfamily genes may have firstly originated from the AP2 family, and through recursive duplications of domain AP2, losses of domain AP2, and acquiring other domains, such as B3, to develop novel genes. This shows interesting evolutionary trajectories of building new genes by using existing domains. Many *AP2* genes have two AP2 domains, which should be initially produced in the alga, which always have multiple AP2 domains. This phenomenon of duplicated domains was frequently observed, producing novel genes, and enriching, enhancing, and expanding gene functions (Wang et al., 2009a), which have recursively contributed to the resistance functions of plants.

The production, expansion, and deletion of tandem duplicated domains should be resulted from unequal DNA crossing-over during meiosis, which is a fundamental DNA recombination mechanism resulting in DNA variations (Keren et al., 2010). However, the mechanisms by which new protein domains arise and diversify are difficult to test experimentally (Black, 2003). It is hypothesized that ancient domains arose by fusion of short peptide ancestors and that they are further diversified by fusion with other domains. Recently, researchers tested how duplicated domains formed through biological experiment (Cahn et al., 2016). They tested the possibility whether the class II ketol-acid reductoisomerase (KARI) have been produced from an ancestral class I KARI by duplication of the C-terminal domain and corresponding loss of obligate dimerization. Eventually, they constructed a novel class II KARI by duplicating the C-terminal domain of a hyperthermostable class I KARI.

## Ethics statement

The study was approved by the North China University of Science and Technology, and Hebei Agricultural University, China. All patients provided written informed consent.

## Author contributions

The study was conceived by XS, XW, JZ. XS, XM, JW, TL, YL, LW, WG, DG, ZW, and CL. contributed to data collection and bioinformatics analysis. XS, XM, XW, and JZ. participated in preparing and writing the manuscript. All authors contributed to revising the manuscript. All authors had read and approved the final manuscript.

### Conflict of interest statement

The authors declare that the research was conducted in the absence of any commercial or financial relationships that could be construed as a potential conflict of interest.

## References

[B1] Albert V. A. Barbazuk W. B. dePamphilis C. W. Der J. P. Leebens-Mack J. Ma H.. (2013). The Amborella genome and the evolution of flowering plants. Science 342:1241089. 10.1126/science.1241089 24357323

[B2] Black D. L. (2003). Mechanisms of alternative pre-messenger RNA splicing. Annu. Rev. Biochem. 72, 291–336. 10.1146/annurev.biochem.72.121801.161720 12626338

[B3] Cahn J. K. Brinkmann-Chen S. Buller A. R. Arnold F. H. (2016). Artificial domain duplication replicates evolutionary history of ketol-acid reductoisomerases. Protein Sci. 25, 1241–1248. 10.1002/pro.2852 26644020 PMC4918412

[B4] Chalhoub B. Denoeud F. Liu S. Parkin I. A. Tang H. Wang X.. (2014). Plant genetics. Early allopolyploid evolution in the post-Neolithic *Brassica napus* oilseed genome. Science 345, 950–953. 10.1126/science.1253435 25146293

[B5] Chen H. Je J. Song C. Hwang J. E. Lim C. O. (2012). A proximal promoter region of Arabidopsis DREB2C confers tissue-specific expression under heat stress. J. Integr. Plant Biol. 54, 640–651. 10.1111/j.1744-7909.2012.01137.x 22716647

[B6] Cheng F. Liu S. Wu J. Fang L. Sun S. Liu B.. (2011). BRAD, the genetics and genomics database for *Brassica* plants. BMC Plant Biol. 11:136. 10.1186/1471-2229-11-136 21995777 PMC3213011

[B7] Cheng F. Wu J. Wang X. (2014). Genome triplication drove the diversification of *Brassica* plants. Hortic. Res. 1, 14024. 10.1038/hortres.2014.24 26504539 PMC4596316

[B8] Cheng M. C. Liao P. M. Kuo W. W. Lin T. P. (2013). The Arabidopsis ETHYLENE RESPONSE FACTOR1 regulates abiotic stress-responsive gene expression by binding to different cis-acting elements in response to different stress signals. Plant Physiol. 162, 1566–1582. 10.1104/pp.113.221911 23719892 PMC3707555

[B9] Cline M. S. Smoot M. Cerami E. Kuchinsky A. Landys N. Workman C.. (2007). Integration of biological networks and gene expression data using Cytoscape. Nat. Protoc. 2, 2366–2382. 10.1038/nprot.2007.324 17947979 PMC3685583

[B10] Djafi N. Vergnolle C. Cantrel C. Wietrzynski W. Delage E. Cochet F.. (2013). The Arabidopsis DREB2 genetic pathway is constitutively repressed by basal phosphoinositide-dependent phospholipase C coupled to diacylglycerol kinase. Front. Plant Sci. 4:307. 10.3389/fpls.2013.00307 23964284 PMC3737466

[B11] Duan C. Argout X. Gebelin V. Summo M. Dufayard J. F. Leclercq J.. (2013). Identification of the *Hevea brasiliensis* AP2/ERF superfamily by RNA sequencing. BMC Genomics 14:30. 10.1186/1471-2164-14-30 23324139 PMC3644242

[B12] Dubois M. Skirycz A. Claeys H. Maleux K. Dhondt S. De Bodt S.. (2013). Ethylene Response Factor6 acts as a central regulator of leaf growth under water-limiting conditions in Arabidopsis. Plant Physiol. 162, 319–332. 10.1104/pp.113.216341 23553636 PMC3641212

[B13] Finn R. D. Bateman A. Clements J. Coggill P. Eberhardt R. Y. Eddy S. R.. (2014). Pfam: the protein families database. Nucleic Acids Res. 42, D222–D230. 10.1093/nar/gkt1223 24288371 PMC3965110

[B14] Gil-Humanes J. Piston F. Martin A. Barro F. (2009). Comparative genomic analysis and expression of the APETALA2-like genes from barley, wheat, and barley-wheat amphiploids. BMC Plant Biol. 9:66. 10.1186/1471-2229-9-66 19480686 PMC2700811

[B15] Gilmour S. J. Zarka D. G. Stockinger E. J. Salazar M. P. Houghton J. M. Thomashow M. F. (1998). Low temperature regulation of the Arabidopsis CBF family of AP2 transcriptional activators as an early step in cold-induced COR gene expression. Plant J. 16, 433–442. 10.1046/j.1365-313x.1998.00310.x 9881163

[B16] Goodstein D. M. Shu S. Howson R. Neupane R. Hayes R. D. Fazo J.. (2012). Phytozome: a comparative platform for green plant genomics. Nucleic Acids Res. 40, D1178–D1186. 10.1093/nar/gkr944 22110026 PMC3245001

[B17] Guindon S. Dufayard J. F. Lefort V. Anisimova M. Hordijk W. Gascuel O. (2010). New algorithms and methods to estimate maximum-likelihood phylogenies: assessing the performance of PhyML 3.0. Syst. Biol. 59, 307–321. 10.1093/sysbio/syq010 20525638

[B18] Guo H. Wang X. Gundlach H. Mayer K. F. Peterson D. G. Scheffler B. E.. (2014). Extensive and biased intergenomic nonreciprocal DNA exchanges shaped a nascent polyploid genome, *Gossypium* (cotton). Genetics 197, 1153–1163. 10.1534/genetics.114.166124 24907262 PMC4125390

[B19] Haake V. Cook D. Riechmann J. L. Pineda O. Thomashow M. F. Zhang J. Z. (2002). Transcription factor CBF4 is a regulator of drought adaptation in Arabidopsis. Plant Physiol. 130, 639–648. 10.1104/pp.006478 12376631 PMC166593

[B20] Hong B. Ma C. Yang Y. Wang T. Yamaguchi-Shinozaki K. Gao J. (2009). Over-expression of AtDREB1A in chrysanthemum enhances tolerance to heat stress. Plant Mol. Biol. 70, 231–240. 10.1007/s11103-009-9468-z 19234675

[B21] Hu B. Jin J. Guo A. Y. Zhang H. Luo J. Gao G. (2015). GSDS 2.0: an upgraded gene feature visualization server. Bioinformatics 31, 1296–1297. 10.1093/bioinformatics/btu817 25504850 PMC4393523

[B22] Hu L. Liu S. (2011). Genome-wide identification and phylogenetic analysis of the ERF gene family in cucumbers. Genet. Mol. Biol. 34, 624–633. 10.1590/S1415-47572011005000054 22215967 PMC3229118

[B23] Ito Y. Katsura K. Maruyama K. Taji T. Kobayashi M. Seki M.. (2006). Functional analysis of rice DREB1/CBF-type transcription factors involved in cold-responsive gene expression in transgenic rice. Plant Cell Physiol. 47, 141–153. 10.1093/pcp/pci230 16284406

[B24] Iwase A. Mitsuda N. Koyama T. Hiratsu K. Kojima M. Arai T.. (2011). The AP2/ERF transcription factor WIND1 controls cell dedifferentiation in Arabidopsis. Curr. Biol. 21, 508–514. 10.1016/j.cub.2011.02.020 21396822

[B25] Jaglo-Ottosen K. R. Gilmour S. J. Zarka D. G. Schabenberger O. Thomashow M. F. (1998). Arabidopsis CBF1 overexpression induces COR genes and enhances freezing tolerance. Science 280, 104–106. 10.1126/science.280.5360.104 9525853

[B26] Jaillon O. Aury J. M. Noel B. Policriti A. Clepet C. Casagrande A.. (2007). The grapevine genome sequence suggests ancestral hexaploidization in major angiosperm phyla. Nature 449, 463–467. 10.1038/nature06148 17721507

[B27] Jiang F. Guo M. Yang F. Duncan K. Jackson D. Rafalski A.. (2012). Mutations in an AP2 transcription factor-like gene affect internode length and leaf shape in maize. PLoS ONE 7:e37040. 10.1371/journal.pone.0037040 22649507 PMC3359370

[B28] Jiao Y. Wickett N. J. Ayyampalayam S. Chanderbali A. S. Landherr L. Ralph P. E.. (2011). Ancestral polyploidy in seed plants and angiosperms. Nature 473, 97–100. 10.1038/nature09916 21478875

[B29] Kawahara Y. de la Bastide M. Hamilton J. P. Kanamori H. McCombie W. R. Ouyang S.. (2013). Improvement of the *Oryza sativa* Nipponbare reference genome using next generation sequence and optical map data. Rice 6:4. 10.1186/1939-8433-6-4 24280374 PMC5395016

[B30] Keren H. Lev-Maor G. Ast G. (2010). Alternative splicing and evolution: diversification, exon definition and function. Nat. Rev. Genet. 11, 345–355. 10.1038/nrg2776 20376054

[B31] Kitomi Y. Ito H. Hobo T. Aya K. Kitano H. Inukai Y. (2011). The auxin responsive AP2/ERF transcription factor CROWN ROOTLESS5 is involved in crown root initiation in rice through the induction of OsRR1, a type-A response regulator of cytokinin signaling. Plant J. 67, 472–484. 10.1111/j.1365-313X.2011.04610.x 21481033

[B32] Koch M. A. Haubold B. Mitchell-Olds T. (2000). Comparative evolutionary analysis of chalcone synthase and alcohol dehydrogenase loci in *Arabidopsis, Arabis*, and related genera (*Brassicaceae*). Mol. Biol. Evol. 17, 1483–1498. 10.1093/oxfordjournals.molbev.a026248 11018155

[B33] Krzywinski M. Schein J. Birol I. Connors J. Gascoyne R. Horsman D.. (2009). Circos: an information aesthetic for comparative genomics. Genome Res. 19, 1639–1645. 10.1101/gr.092759.109 19541911 PMC2752132

[B34] Lamesch P. Berardini T. Z. Li D. Swarbreck D. Wilks C. Sasidharan R.. (2012). The Arabidopsis Information Resource (TAIR): improved gene annotation and new tools. Nucleic Acids Res. 40, D1202–D1210. 10.1093/nar/gkr1090 22140109 PMC3245047

[B35] Lata C. Prasad M. (2011). Role of DREBs in regulation of abiotic stress responses in plants. J. Exp. Bot. 62, 4731–4748. 10.1093/jxb/err210 21737415

[B36] Lee S. C. Choi D. S. Hwang I. S. Hwang B. K. (2010). The pepper oxidoreductase CaOXR1 interacts with the transcription factor CaRAV1 and is required for salt and osmotic stress tolerance. Plant Mol. Biol. 73, 409–424. 10.1007/s11103-010-9629-0 20333442

[B37] Letunic I. Doerks T. Bork P. (2012). SMART 7: recent updates to the protein domain annotation resource. Nucleic Acids Res. 40, D302–D305. 10.1093/nar/gkr931 22053084 PMC3245027

[B38] Li L. Stoeckert C. J. Jr. Roos D. S. (2003). OrthoMCL: identification of ortholog groups for eukaryotic genomes. Genome Res. 13, 2178–2189. 10.1101/gr.1224503 12952885 PMC403725

[B39] Li M. R. Li Y. Li H. Q. Wu G. J. (2011). Ectopic expression of FaDREB2 enhances osmotic tolerance in paper mulberry. J. Integr. Plant Biol. 53, 951–960. 10.1111/j.1744-7909.2011.01087.x 22067051

[B40] Li X. Zhang D. Li H. Wang Y. Zhang Y. Wood A. J. (2014). EsDREB2B, a novel truncated DREB2-type transcription factor in the desert legume *Eremosparton songoricum*, enhances tolerance to multiple abiotic stresses in yeast and transgenic tobacco. BMC Plant Biol. 14:44. 10.1186/1471-2229-14-44 24506952 PMC3940028

[B41] Licausi F. Giorgi F. M. Zenoni S. Osti F. Pezzotti M. Perata P. (2010). Genomic and transcriptomic analysis of the AP2/ERF superfamily in *Vitis vinifera*. BMC Genomics 11:719. 10.1186/1471-2164-11-719 21171999 PMC3022922

[B42] Licausi F. Ohme-Takagi M. Perata P. (2013). APETALA2/Ethylene Responsive Factor (AP2/ERF) transcription factors: mediators of stress responses and developmental programs. New Phytol. 199, 639–649. 10.1111/nph.12291 24010138

[B43] Lim C. J. Hwang J. E. Chen H. Hong J. K. Yang K. A. Choi M. S.. (2007). Over-expression of the Arabidopsis DRE/CRT-binding transcription factor DREB2C enhances thermotolerance. Biochem. Biophys. Res. Commun. 362, 431–436. 10.1016/j.bbrc.2007.08.007 17716623

[B44] Liu S. Liu Y. Yang X. Tong C. Edwards D. Parkin I. A.. (2014). The *Brassica oleracea* genome reveals the asymmetrical evolution of polyploid genomes. Nat. Commun. 5, 3930. 10.1038/ncomms4930 24852848 PMC4279128

[B45] Luo H. Chen S. Jiang J. Teng N. Chen Y. Chen F. (2012). The AP2-like gene NsAP2 from water lily is involved in floral organogenesis and plant height. J. Plant Physiol. 169, 992–998. 10.1016/j.jplph.2012.02.018 22591856

[B46] Mehrnia M. Balazadeh S. Zanor M. I. Mueller-Roeber B. (2013). EBE, an AP2/ERF transcription factor highly expressed in proliferating cells, affects shoot architecture in Arabidopsis. Plant Physiol. 162, 842–857. 10.1104/pp.113.214049 23616605 PMC3668074

[B47] Mondragon-Palomino M. Gaut B. S. (2005). Gene conversion and the evolution of three leucine-rich repeat gene families in *Arabidopsis thaliana*. Mol. Biol. Evol. 22, 2444–2456. 10.1093/molbev/msi241 16120808

[B48] Mondragon-Palomino M. Meyers B. C. Michelmore R. W. Gaut B. S. (2002). Patterns of positive selection in the complete NBS-LRR gene family of *Arabidopsis thaliana*. Genome Res. 12, 1305–1315. 10.1101/gr.159402 12213767 PMC186657

[B49] Nakano T. Suzuki K. Fujimura T. Shinshi H. (2006). Genome-wide analysis of the ERF gene family in Arabidopsis and rice. Plant Physiol. 140, 411–432. 10.1104/pp.105.073783 16407444 PMC1361313

[B50] Nakashima K. Shinwari Z. K. Sakuma Y. Seki M. Miura S. Shinozaki K.. (2000). Organization and expression of two Arabidopsis DREB2 genes encoding DRE-binding proteins involved in dehydration- and high-salinity-responsive gene expression. Plant Mol. Biol. 42, 657–665. 10.1023/A:1006321900483 10809011

[B51] Ozkan H. Levy A. A. Feldman M. (2001). Allopolyploidy-induced rapid genome evolution in the wheat (Aegilops-Triticum) group. Plant Cell 13, 1735–1747. 10.1105/tpc.13.8.1735 11487689 PMC139130

[B52] Ozkan H. Levy A. A. Feldman M. (2002). Rapid differentiation of homeologous chromosomes in newly-formed allopolyploid wheat. Isr. J. Plant Sci. 50, S65–S76. 10.1560/E282-PV55-G4XT-DRWJ

[B53] Parkin I. A. Koh C. Tang H. Robinson S. J. Kagale S. Clarke W. E.. (2014). Transcriptome and methylome profiling reveals relics of genome dominance in the mesopolyploid *Brassica oleracea*. Genome Biol. 15:R77. 10.1186/gb-2014-15-6-r77 24916971 PMC4097860

[B54] Paterson A. H. Wendel J. F. Gundlach H. Guo H. Jenkins J. Jin D.. (2012). Repeated polyploidization of *Gossypium* genomes and the evolution of spinnable cotton fibres. Nature 492, 423–427. 10.1038/nature11798 23257886

[B55] Peters S. A. Datema E. Szinay D. van Staveren M. J. Schijlen E. G. van Haarst J. C.. (2009). *Solanum lycopersicum* cv. Heinz 1706 chromosome 6: distribution and abundance of genes and retrotransposable elements. Plant J. 58, 857–869. 10.1111/j.1365-313X.2009.03822.x 19207213

[B56] Qin F. Kakimoto M. Sakuma Y. Maruyama K. Osakabe Y. Tran L. S.. (2007). Regulation and functional analysis of ZmDREB2A in response to drought and heat stresses in *Zea mays* L. Plant J. 50, 54–69. 10.1111/j.1365-313X.2007.03034.x 17346263

[B57] Ren D. Li Y. Zhao F. Sang X. Shi J. Wang N.. (2013). MULTI-FLORET SPIKELET1, which encodes an AP2/ERF protein, determines spikelet meristem fate and sterile lemma identity in rice. Plant Physiol. 162, 872–884. 10.1104/pp.113.216044 23629832 PMC3668076

[B58] Sakuma Y. Liu Q. Dubouzet J. G. Abe H. Shinozaki K. Yamaguchi-Shinozaki K. (2002). DNA-binding specificity of the ERF/AP2 domain of Arabidopsis DREBs, transcription factors involved in dehydration- and cold-inducible gene expression. Biochem. Biophys. Res. Commun. 290, 998–1009. 10.1006/bbrc.2001.6299 11798174

[B59] Sakuma Y. Maruyama K. Osakabe Y. Qin F. Seki M. Shinozaki K.. (2006). Functional analysis of an Arabidopsis transcription factor, DREB2A, involved in drought-responsive gene expression. Plant Cell 18, 1292–1309. 10.1105/tpc.105.035881 16617101 PMC1456870

[B60] Schmidt R. Mieulet D. Hubberten H. M. Obata T. Hoefgen R. Fernie A. R.. (2013). Salt-responsive ERF1 regulates reactive oxygen species-dependent signaling during the initial response to salt stress in rice. Plant Cell 25, 2115–2131. 10.1105/tpc.113.113068 23800963 PMC3723616

[B61] Schnable J. C. Springer N. M. Freeling M. (2011). Differentiation of the maize subgenomes by genome dominance and both ancient and ongoing gene loss. Proc. Natl. Acad. Sci. U.S.A. 108, 4069–4074. 10.1073/pnas.1101368108 21368132 PMC3053962

[B62] Shang J. Song P. Ma B. Qi X. Zeng Q. Xiang Z.. (2014). Identification of the mulberry genes involved in ethylene biosynthesis and signaling pathways and the expression of MaERF-B2-1 and MaERF-B2-2 in the response to flooding stress. Funct. Integr. Genomics 14, 767–777. 10.1007/s10142-014-0403-2 25231943 PMC4233114

[B63] Shoji T. Mishima M. Hashimoto T. (2013). Divergent DNA-binding specificities of a group of ETHYLENE RESPONSE FACTOR transcription factors involved in plant defense. Plant Physiol. 162, 977–990. 10.1104/pp.113.217455 23629834 PMC3668085

[B64] Soltesz A. Smedley M. Vashegyi I. Galiba G. Harwood W. Vagujfalvi A. (2013). Transgenic barley lines prove the involvement of TaCBF14 and TaCBF15 in the cold acclimation process and in frost tolerance. J. Exp. Bot. 64, 1849–1862. 10.1093/jxb/ert050 23567863 PMC3638819

[B65] Song X. Duan W. Huang Z. Liu G. Wu P. Liu T.. (2015). Comprehensive analysis of the flowering genes in Chinese cabbage and examination of evolutionary pattern of CO-like genes in plant kingdom. Sci. Rep. 5:14631. 10.1038/srep14631 26416765 PMC4586889

[B68] Song X. Li Y. Hou X. (2013). Genome-wide analysis of the AP2/ERF transcription factor superfamily in Chinese cabbage (*Brassica rapa* ssp. pekinensis). BMC Genomics 14:573. 10.1186/1471-2164-14-573 23972083 PMC3765354

[B66] Song X. Liu G. Duan W. Liu T. Huang Z. Ren J.. (2014a). Genome-wide identification, classification and expression analysis of the heat shock transcription factor family in Chinese cabbage. Mol. Genet. Genomics 289, 541–551. 10.1007/s00438-014-0833-5 24609322

[B67] Song X. Liu T. Duan W. Ma Q. Ren J. Wang Z.. (2014b). Genome-wide analysis of the GRAS gene family in Chinese cabbage (*Brassica rapa* ssp. pekinensis). Genomics 103, 135–146. 10.1016/j.ygeno.2013.12.004 24365788

[B69] Tamura K. Stecher G. Peterson D. Filipski A. Kumar S. (2013). MEGA6: molecular evolutionary genetics analysis version 6.0. Mol. Biol. Evol. 30, 2725–2729. 10.1093/molbev/mst197 24132122 PMC3840312

[B70] Thamilarasan S. K. Park J. I. Jung H. J. Nou I. S. (2014). Genome-wide analysis of the distribution of AP2/ERF transcription factors reveals duplication and CBFs genes elucidate their potential function in *Brassica oleracea*. BMC Genomics 15:422. 10.1186/1471-2164-15-422 24888752 PMC4229850

[B71] Tuskan G. A. Difazio S. Jansson S. Bohlmann J. Grigoriev I. Hellsten U.. (2006). The genome of black cottonwood, *Populus trichocarpa* (Torr. and Gray). Science 313, 1596–1604. 10.1126/science.1128691 16973872

[B72] Wang D. Zhang Y. Zhang Z. Zhu J. Yu J. (2010). KaKs_Calculator 2.0: a toolkit incorporating gamma-series methods and sliding window strategies. Genomics Proteomics Bioinformatics 8, 77–80. 10.1016/S1672-0229(10)60008-3 20451164 PMC5054116

[B73] Wang L. Wang C. Qin L. Liu W. Wang Y. (2015a). ThERF1 regulates its target genes via binding to a novel cis-acting element in response to salt stress. J. Integr. Plant Biol. 57, 838–847. 10.1111/jipb.12335 25641039

[B74] Wang X. Gowik U. Tang H. Bowers J. E. Westhoff P. Paterson A. H. (2009a). Comparative genomic analysis of C4 photosynthetic pathway evolution in grasses. Genome Biol. 10:R68. 10.1186/gb-2009-10-6-r68 19549309 PMC2718502

[B75] Wang X. Tang H. Bowers J. E. Paterson A. H. (2009b). Comparative inference of illegitimate recombination between rice and sorghum duplicated genes produced by polyploidization. Genome Res. 19, 1026–1032. 10.1101/gr.087288.108 19372385 PMC2694483

[B76] Wang X. Wang H. Wang J. Sun R. Wu J. Liu S.. (2011). The genome of the mesopolyploid crop species *Brassica rapa*. Nat. Genet. 43, 1035–1039. 10.1038/ng.919 21873998

[B77] Wang X. Wang J. Jin D. Guo H. Lee T. H. Liu T.. (2015b). Genome alignment spanning major poaceae lineages reveals heterogeneous evolutionary rates and alters inferred dates for key evolutionary events. Mol. Plant 8, 885–898. 10.1016/j.molp.2015.04.004 25896453

[B78] Wang X. Y. Paterson A. H. (2011). Gene conversion in angiosperm genomes with an emphasis on genes duplicated by polyploidization. Genes (Basel) 2, 1–20. 10.3390/genes2010001 24710136 PMC3924838

[B79] Wang Y. Tang H. Debarry J. D. Tan X. Li J. Wang X.. (2012). MCScanX: a toolkit for detection and evolutionary analysis of gene synteny and collinearity. Nucleic Acids Res. 40, e49. 10.1093/nar/gkr1293 22217600 PMC3326336

[B80] Woodhouse M. R. Cheng F. Pires J. C. Lisch D. Freeling M. Wang X. (2014). Origin, inheritance, and gene regulatory consequences of genome dominance in polyploids. Proc. Natl. Acad. Sci. U.S.A. 111, 5283–5288. 10.1073/pnas.1402475111 24706847 PMC3986174

[B81] Woodhouse M. R. Schnable J. C. Pedersen B. S. Lyons E. Lisch D. Subramaniam S.. (2010). Following tetraploidy in maize, a short deletion mechanism removed genes preferentially from one of the two homologs. PLoS Biol. 8:e1000409. 10.1371/journal.pbio.1000409 20613864 PMC2893956

[B82] Xu Q. Chen L. L. Ruan X. Chen D. Zhu A. Chen C.. (2013). The draft genome of sweet orange (*Citrus sinensis*). Nat. Genet. 45, 59–66. 10.1038/ng.2472 23179022

[B83] Xu Z. S. Chen M. Li L. C. Ma Y. Z. (2011). Functions and application of the AP2/ERF transcription factor family in crop improvement. J. Integr. Plant Biol. 53, 570–585. 10.1111/j.1744-7909.2011.01062.x 21676172

[B84] Yan X. Zhang L. Chen B. Xiong Z. Chen C. Wang L.. (2012). Functional identification and characterization of the *Brassica napus* transcription factor gene BnAP2, the ortholog of *Arabidopsis thaliana* APETALA2. PLoS ONE 7:e33890. 10.1371/journal.pone.0033890 22479468 PMC3314020

[B85] Yang Z. (1997). PAML: a program package for phylogenetic analysis by maximum likelihood. Comput. Appl. Biosci. 13, 555–556. 10.1093/bioinformatics/13.5.555 9367129

[B86] Yang Z. Nielsen R. Goldman N. Pedersen A. M. (2000). Codon-substitution models for heterogeneous selection pressure at amino acid sites. Genetics 155, 431–449. 10790415 10.1093/genetics/155.1.431 PMC1461088

[B87] Zeng L. Yin Y. You C. Pan Q. Xu D. Jin T.. (2016). Evolution and protein interactions of AP2 proteins in *Brassicaceae*: evidence linking development and environmental responses. J. Integr. Plant Biol. 58, 549–563. 10.1111/jipb.12439 26472270

[B88] Zhang G. Chen M. Chen X. Xu Z. Guan S. Li L. C.. (2008). Phylogeny, gene structures, and expression patterns of the ERF gene family in soybean (*Glycine max* L.). J. Exp. Bot. 59, 4095–4107. 10.1093/jxb/ern248 18832187 PMC2639015

[B89] Zhang H. Liu W. Wan L. Li F. Dai L. Li D.. (2010). Functional analyses of ethylene response factor JERF3 with the aim of improving tolerance to drought and osmotic stress in transgenic rice. Transgenic Res. 19, 809–818. 10.1007/s11248-009-9357-x 20087656

[B90] Zhang X. Liu X. Wu L. Yu G. Wang X. Ma H. (2015). The SsDREB transcription factor from the succulent halophyte *Suaeda salsa* enhances abiotic stress tolerance in transgenic tobacco. Int. J. Genomics 2015:875497. 10.1155/2015/875497 26504772 PMC4609462

[B91] Zhang Z. Huang R. (2010). Enhanced tolerance to freezing in tobacco and tomato overexpressing transcription factor TERF2/LeERF2 is modulated by ethylene biosynthesis. Plant Mol. Biol. 73, 241–249. 10.1007/s11103-010-9609-4 20135196

[B92] Zhu X. Qi L. Liu X. Cai S. Xu H. Huang R.. (2014). The wheat ethylene response factor transcription factor pathogen-induced ERF1 mediates host responses to both the necrotrophic pathogen Rhizoctonia cerealis and freezing stresses. Plant Physiol. 164, 1499–1514. 10.1104/pp.113.229575 24424323 PMC3938636

[B93] Zhuang J. Cai B. Peng R. H. Zhu B. Jin X. F. Xue Y.. (2008). Genome-wide analysis of the AP2/ERF gene family in *Populus trichocarpa*. Biochem. Biophys. Res. Commun. 371, 468–474. 10.1016/j.bbrc.2008.04.087 18442469

[B94] Zhuang J. Chen J. M. Yao Q. H. Xiong F. Sun C. C. Zhou X. R.. (2011a). Discovery and expression profile analysis of AP2/ERF family genes from *Triticum aestivum*. Mol. Biol. Rep. 38, 745–753. 10.1007/s11033-010-0162-7 20407836

[B95] Zhuang J. Sun C. C. Zhou X. R. Xiong A. S. Zhang J. (2011b). Isolation and characterization of an AP2/ERF-RAV transcription factor BnaRAV-1-HY15 in *Brassica napus* L. HuYou15. Mol. Biol. Rep. 38, 3921–3928. 10.1007/s11033-010-0508-1 21116861

