# Supplementary material for: Origination, Expansion, Evolutionary Trajectory, and Expression Bias of AP2/ERF Superfamily in Brassica napus
Source: Front Plant Sci. 2016 Aug 12;7:1186. doi: 10.3389/fpls.2016.01186 (PMC4982375; doi:10.3389/fpls.2016.01186)
Supplement: Supplementary file 2 [file DataSheet1.PDF]

Supplementary figures (S1-S2, S4-S11, S13)

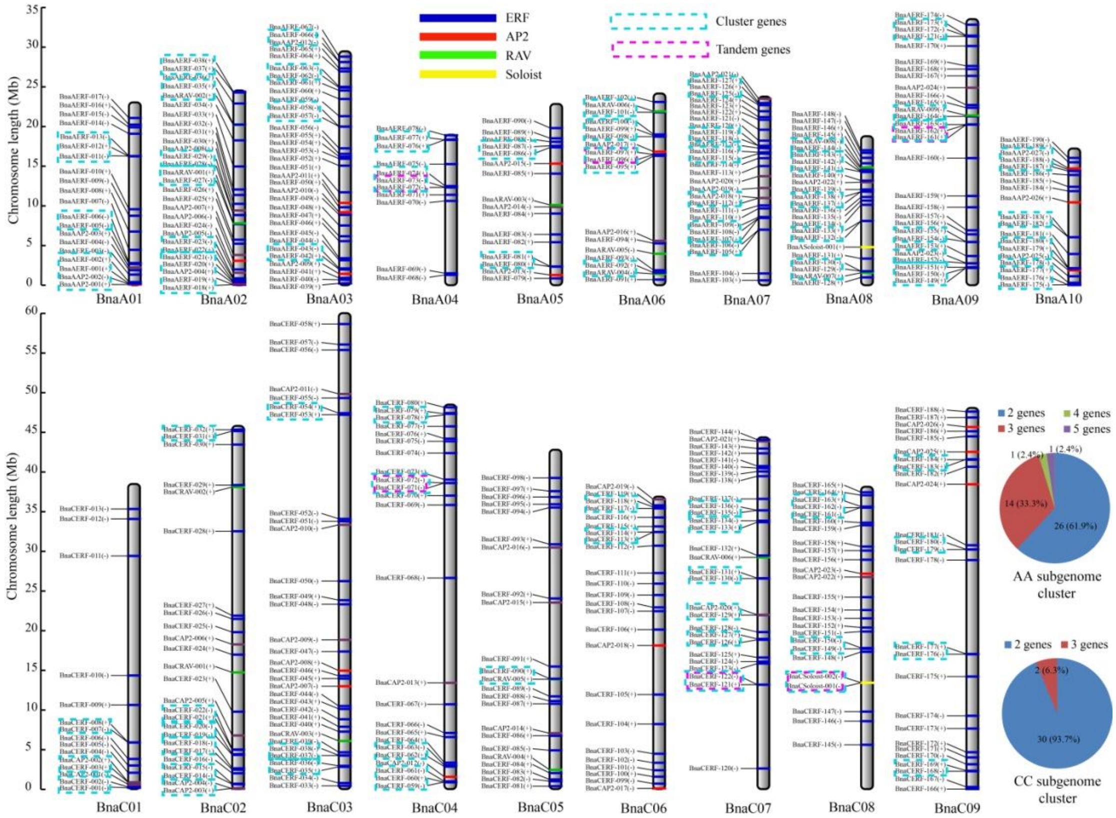

Figure S1. Distribution of AP2/ERF genes on 19 chromosomes in *B. napus*. The pink dashed frame indicated the tandem duplicated AP2/ERF genes. The light-blue dashed frame indicated the gene clusters defined as two or more AP2/ERF genes existed within 200Kb on chromosome.

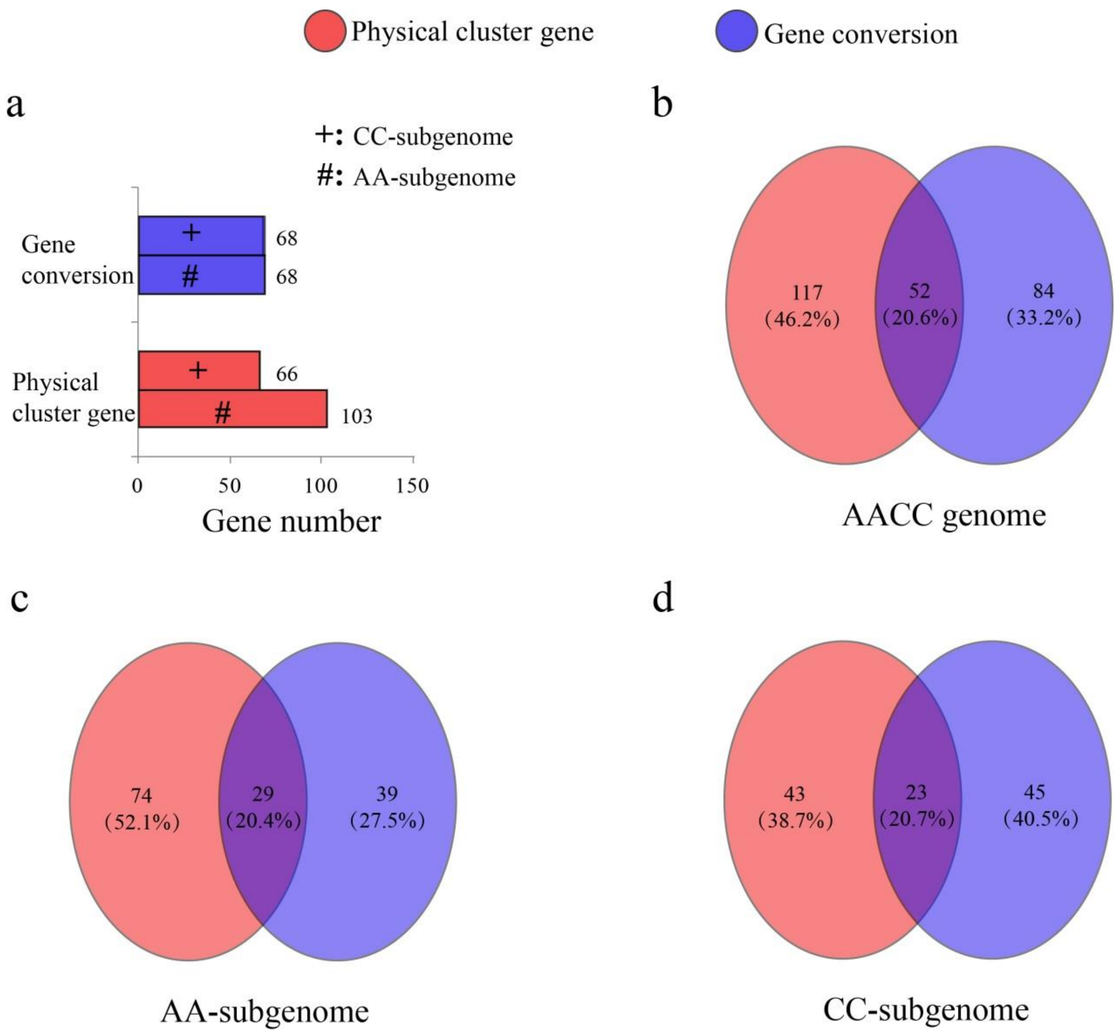

Figure S2. The gene conversion and physical cluster analysis of *B. napus* genome. **(a)** The number of these twp types gene sets in AA- and CC-subgenome of *B. napus*. **(b)** The venn diagram of these two types gene sets in *B. napus* genome. **(c)** The venn diagram of these two types gene sets in AA-subgenome. **(d)** The venn diagram of these two types gene sets in CC-subgenome.

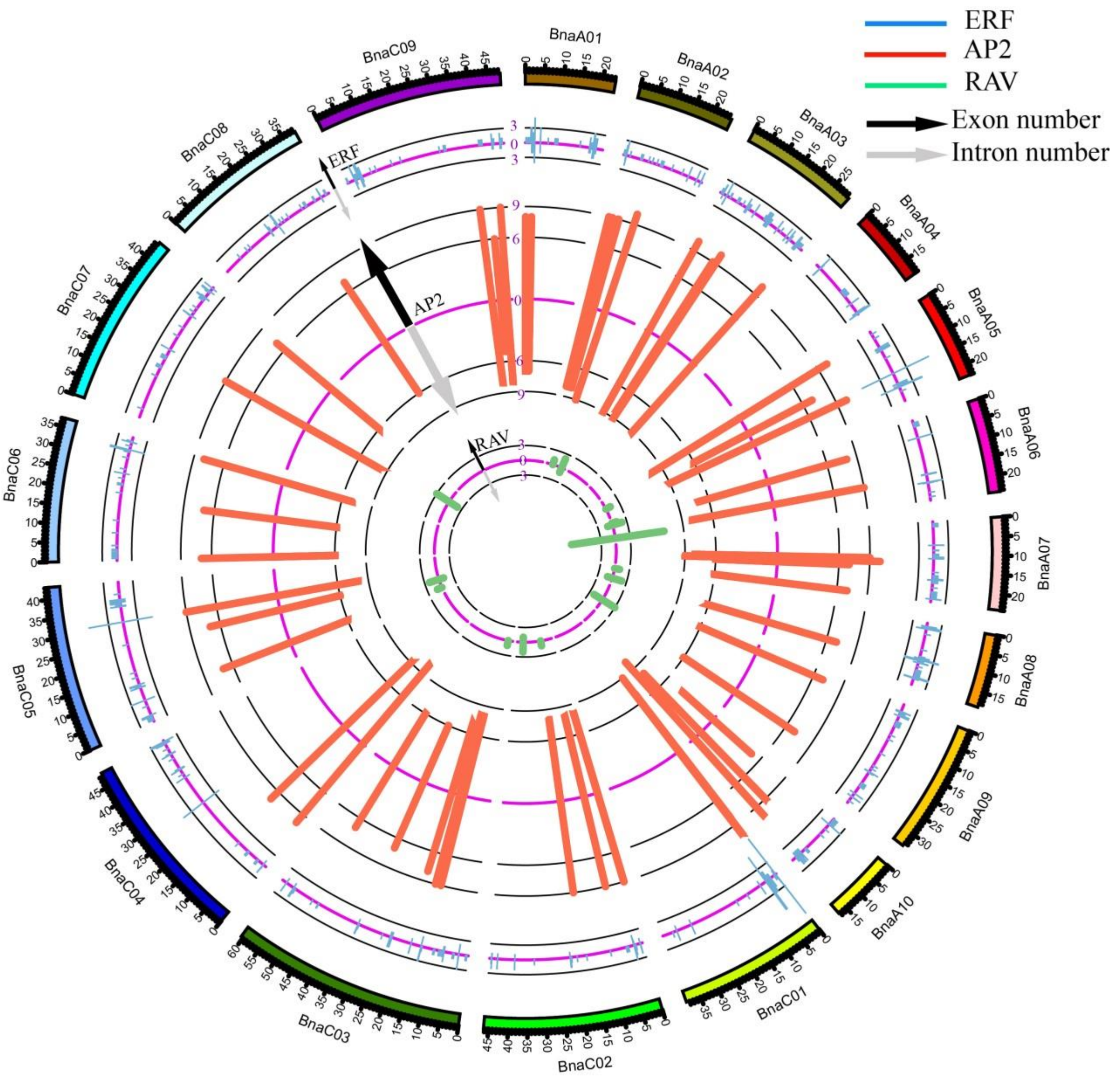

Figure S4. The circle graph showed the exon and intron number of ERF, AP2, RAV family genes from outer to inner. The outermost circle represent the 19 chromosomes of *B. napus*, including 10 chromosomes from AA genome (BnaA01~BnaA10), and 9 chromosomes from CC genome (BnaC01~BnaC09).

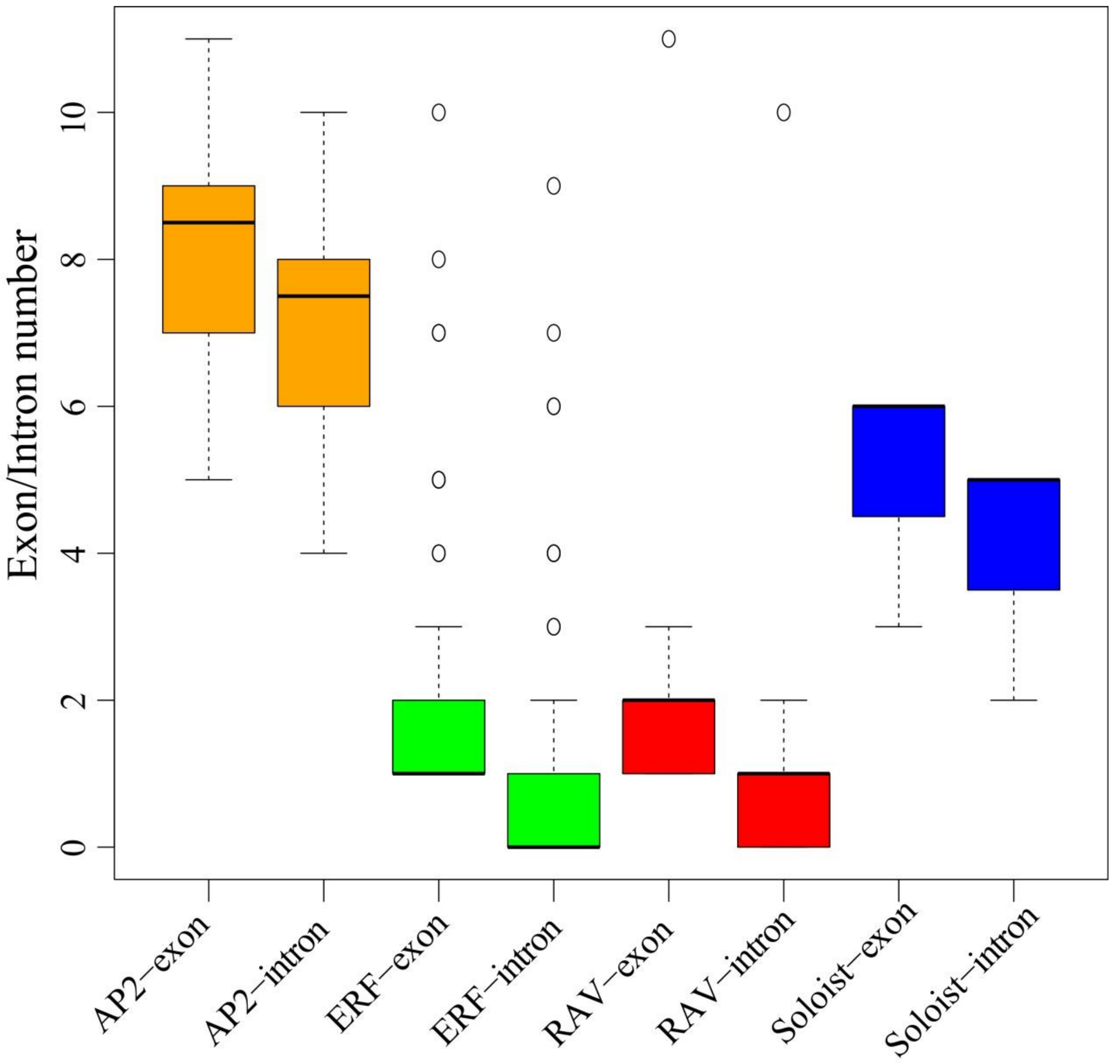

Figure S5. The boxplot of exon and intron number of ERF, AP2, RAV, and Soloist family genes.

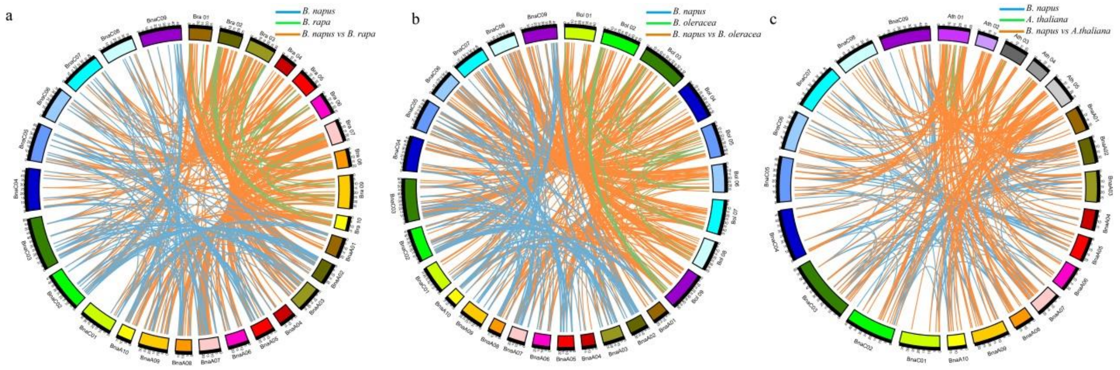

Figure S6. (a) The 19 *B. napus* chromosomes and 10 *B. rapa* chromosomes (Bra 01~Bra 10) maps were based on orthologous and paralogous pair positions. **(b)** The 19 *B. napus* chromosomes and 9 *B. oleracea* chromosomes (Bol 01~Bol 09) maps were based on orthologous and paralogous pair positions. **(c)** The 19 *B. napus* chromosomes and 5 *A. thaliana* chromosomes (Ath 01~ Ath 05) maps were based on orthologous and paralogous pairs positions.

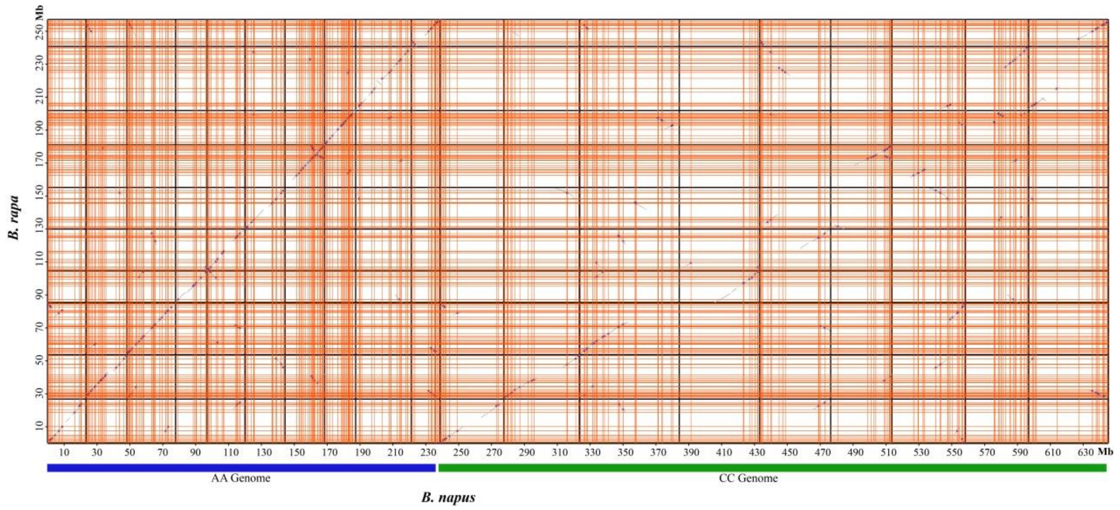

Figure S7. The collinearity blocks identified in the whole genome and the AP2/ERF genes between *B. napus* and *B. rapa*. The blue dots represent the AP2/ERF genes located in the collinearity blocks.

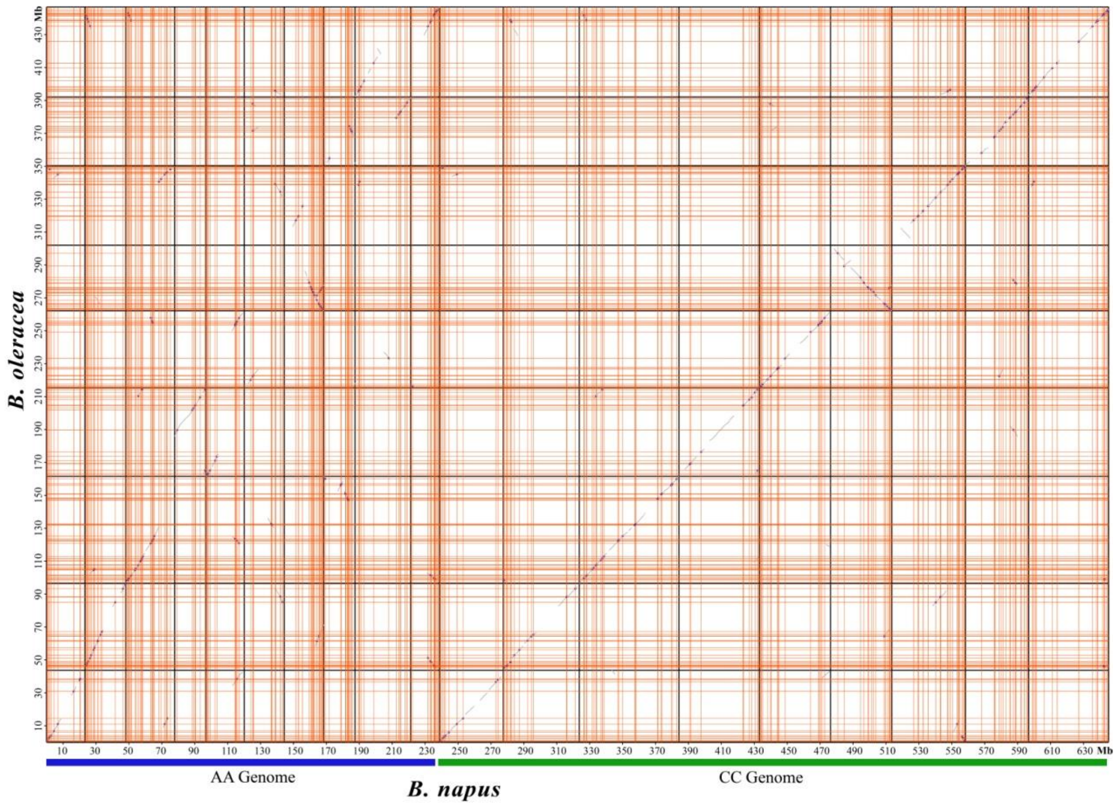

Figure S8. The collinearity blocks identified in the whole genome and the AP2/ERF genes between *B. napus* and *B. oleracea*. The blue dots represent the AP2/ERF genes located in the collinearity blocks.

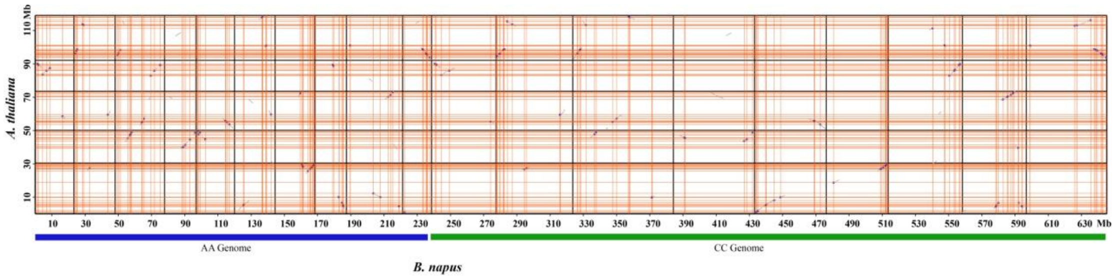

Figure S9. The collinearity blocks identified in the whole genome and the AP2/ERF genes between *B. napus* and *A. thaliana*. The blue dots represent the AP2/ERF genes located in the collinearity blocks.

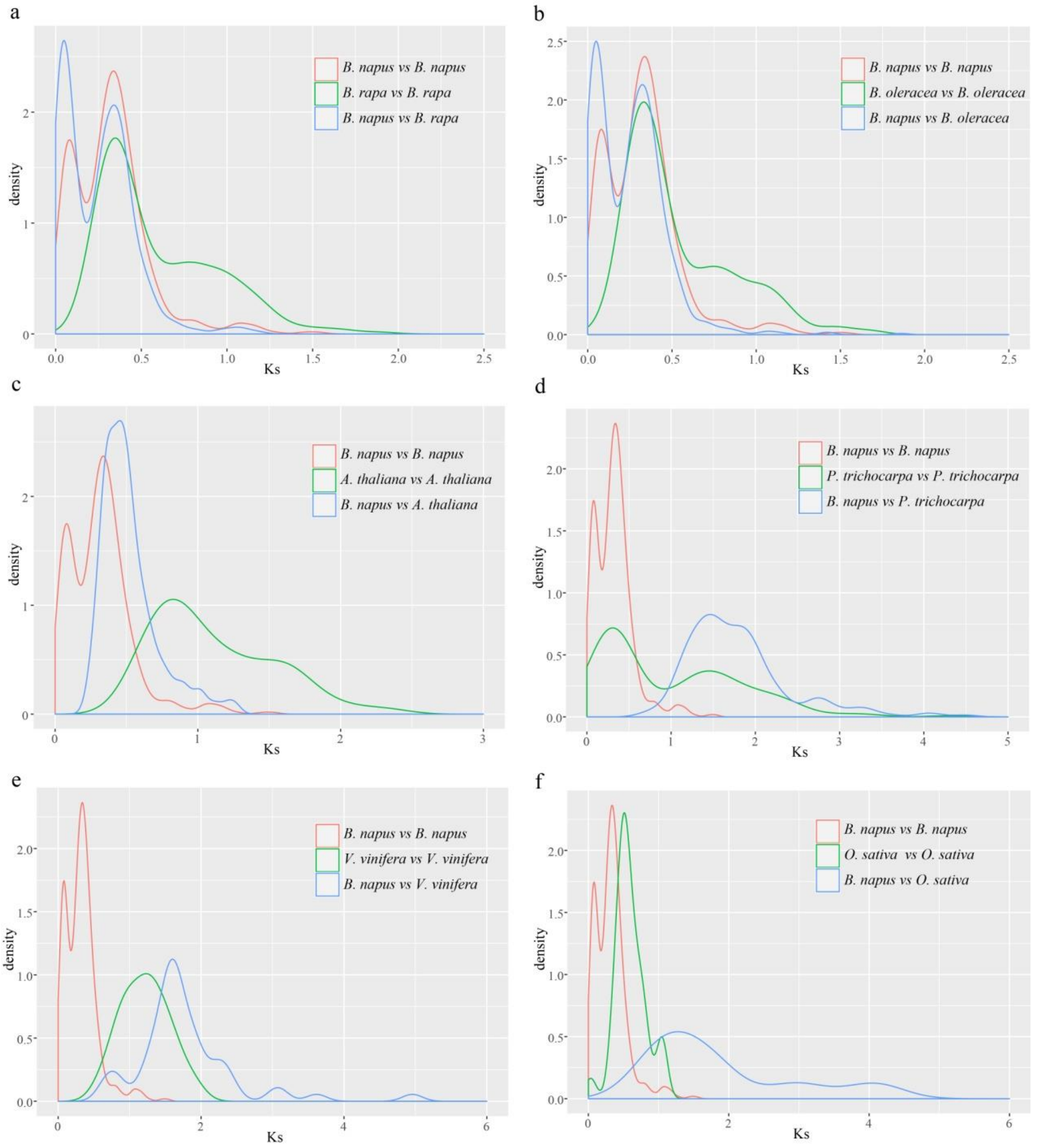

Figure S10. The Ks analyses for the *B. napus* and the other related species. (a) The density of the Ks values for syntenic AP2/ERF gene pairs of *B.napus*, *B. rapa* and between of them. **(b)** The density of the Ks values for syntenic AP2/ERF gene pairs of *B. napus*, *B. oleracea* and between of them. **(c)** The density of the Ks values for syntenic AP2/ERF gene pairs of *B. napus*, *A. thaliana* and between of them. **(d)** The density of the Ks values for syntenic AP2/ERF gene pairs of *B. napus*, *P. trichocarpa* and between of them. **(e)** The density of the Ks values for syntenic AP2/ERF gene pairs of *B. napus*, *V. vinifera* and between of them. **(f)** The density of the Ks values for syntenic AP2/ERF gene pairs of *B. napus*, *O. sativa* and between of them.

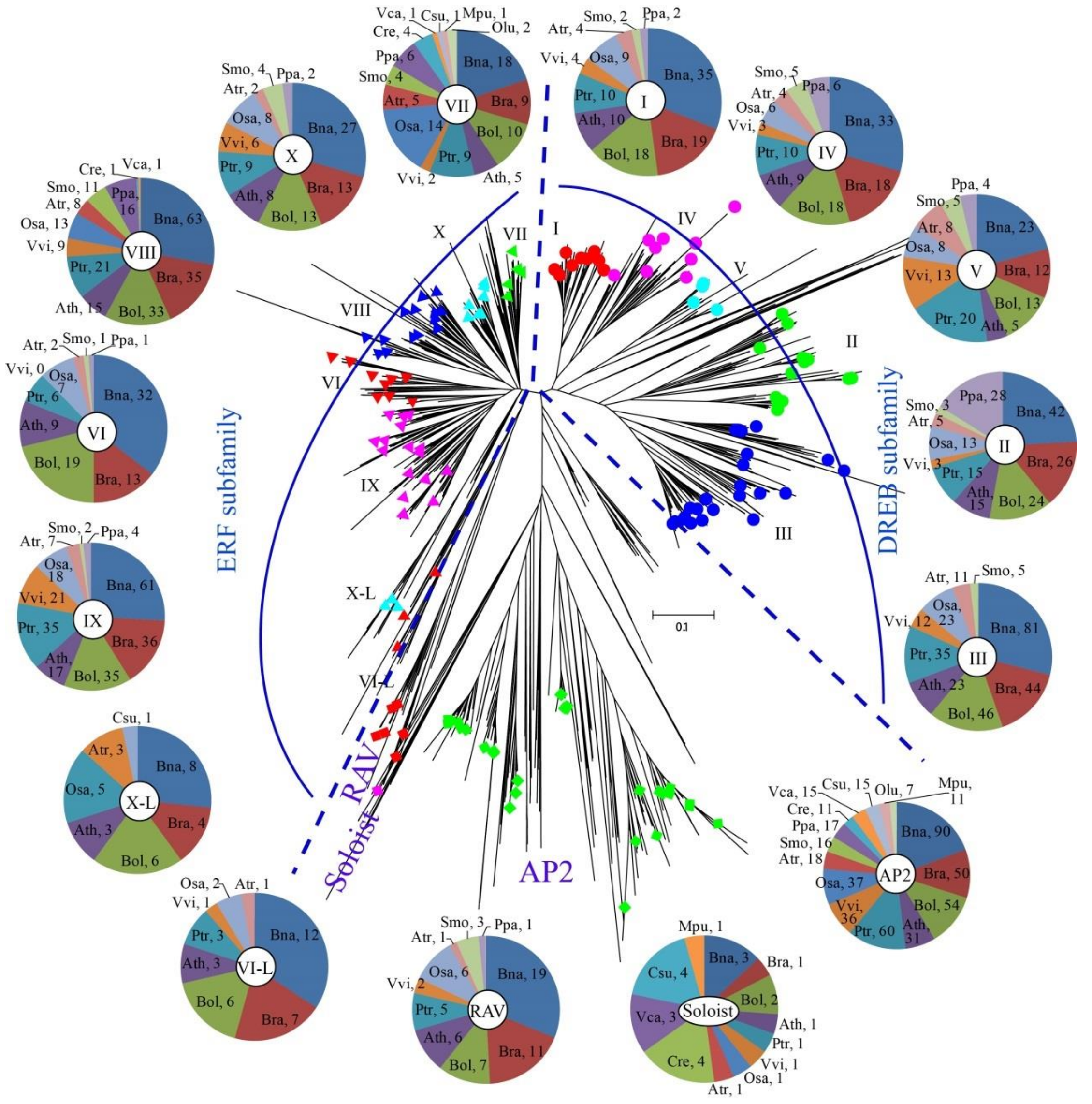

Figure S11. Phylogenetic tree constructed using the neighbor-joining method by MEGA6, using the AP2 domain sequences of AP2/ERF superfamily genes in *B. napus* and other 14 species. The pie graph showed the AP2 domain number for each species in corresponding group.

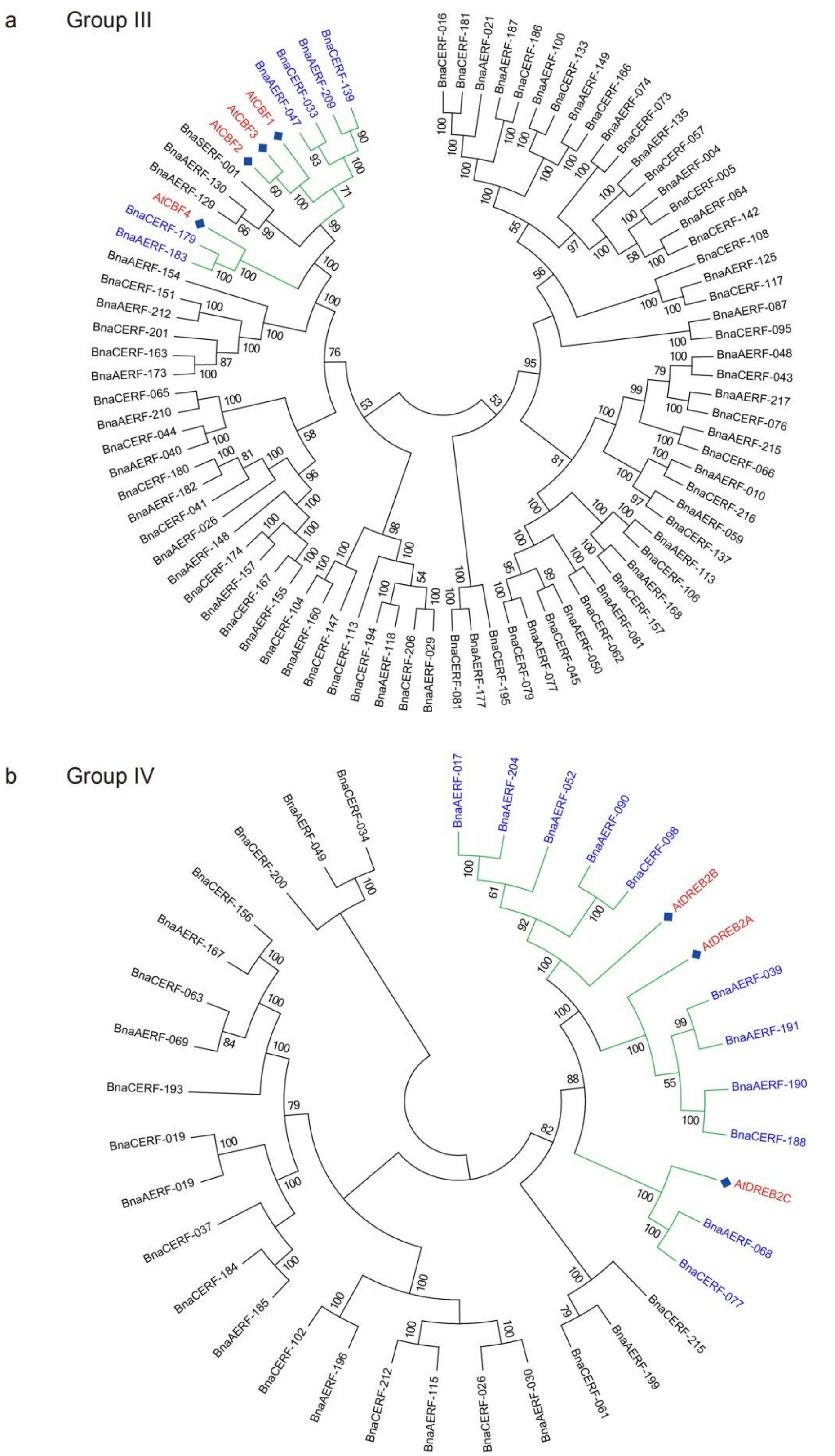

Figure S13. The phylogenetic trees of well characterized *Arabidopsis* genes and some *AP2/ERF* genes in *B. napus*. (a) The *AtCBF1* to *AtCBF4* genes and Group III *AP2/ERF* genes in *B. napus*. (b) The *AtDREB2A* to *AtDREB2C* genes and Group IV *AP2/ERF* genes in *B. napus*.
