# Supplementary material for: Origination, Expansion, Evolutionary Trajectory, and Expression Bias of AP2/ERF Superfamily in Brassica napus
Source: Front Plant Sci. 2016 Aug 12;7:1186. doi: 10.3389/fpls.2016.01186 (PMC4982375; doi:10.3389/fpls.2016.01186)
Supplement: Supplementary file 3 [file DataSheet2.PDF]

Figure S3. The Phylogenetic tree and gene structure analyses for each AP2/ERF group in B. napus.

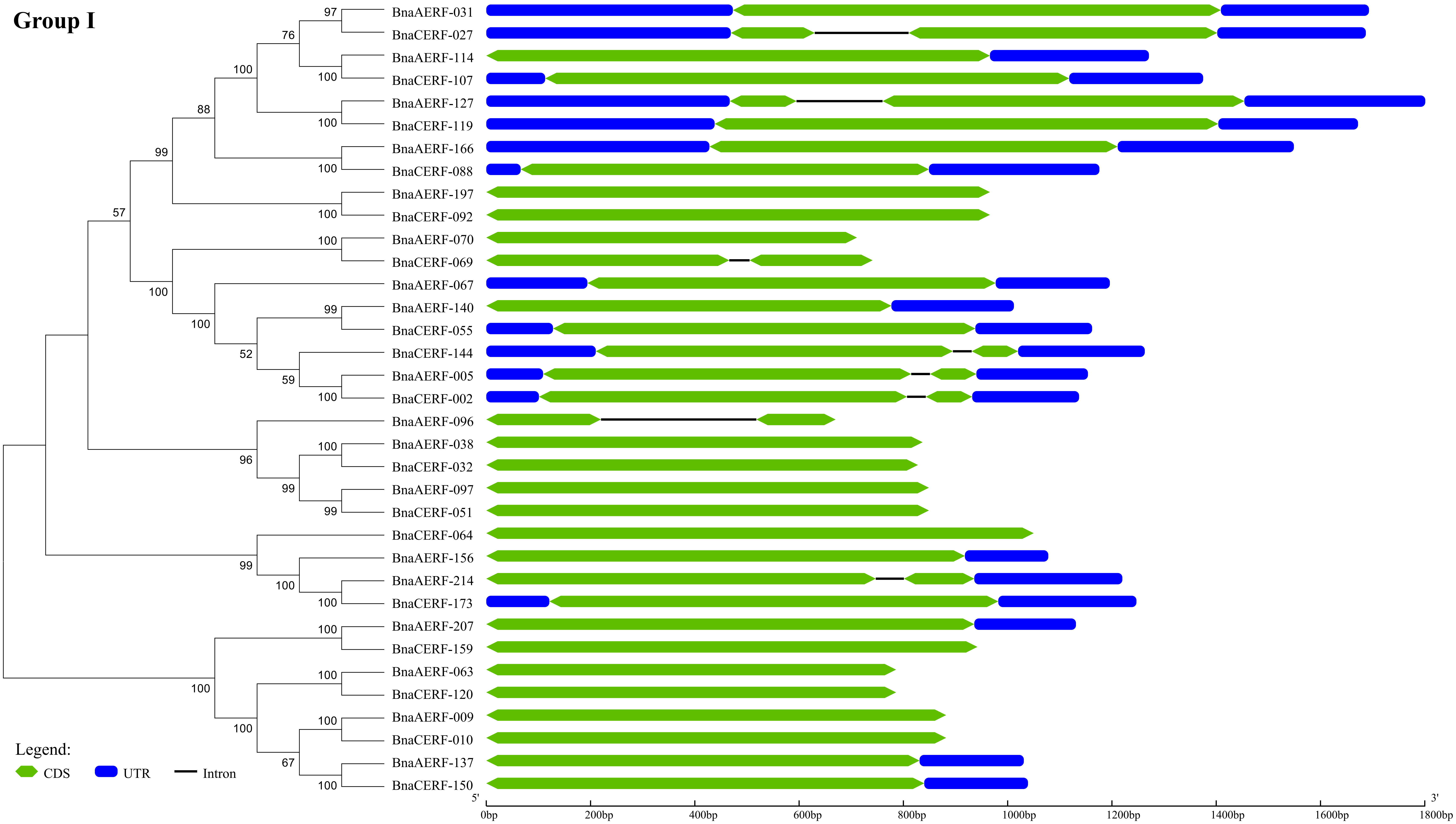

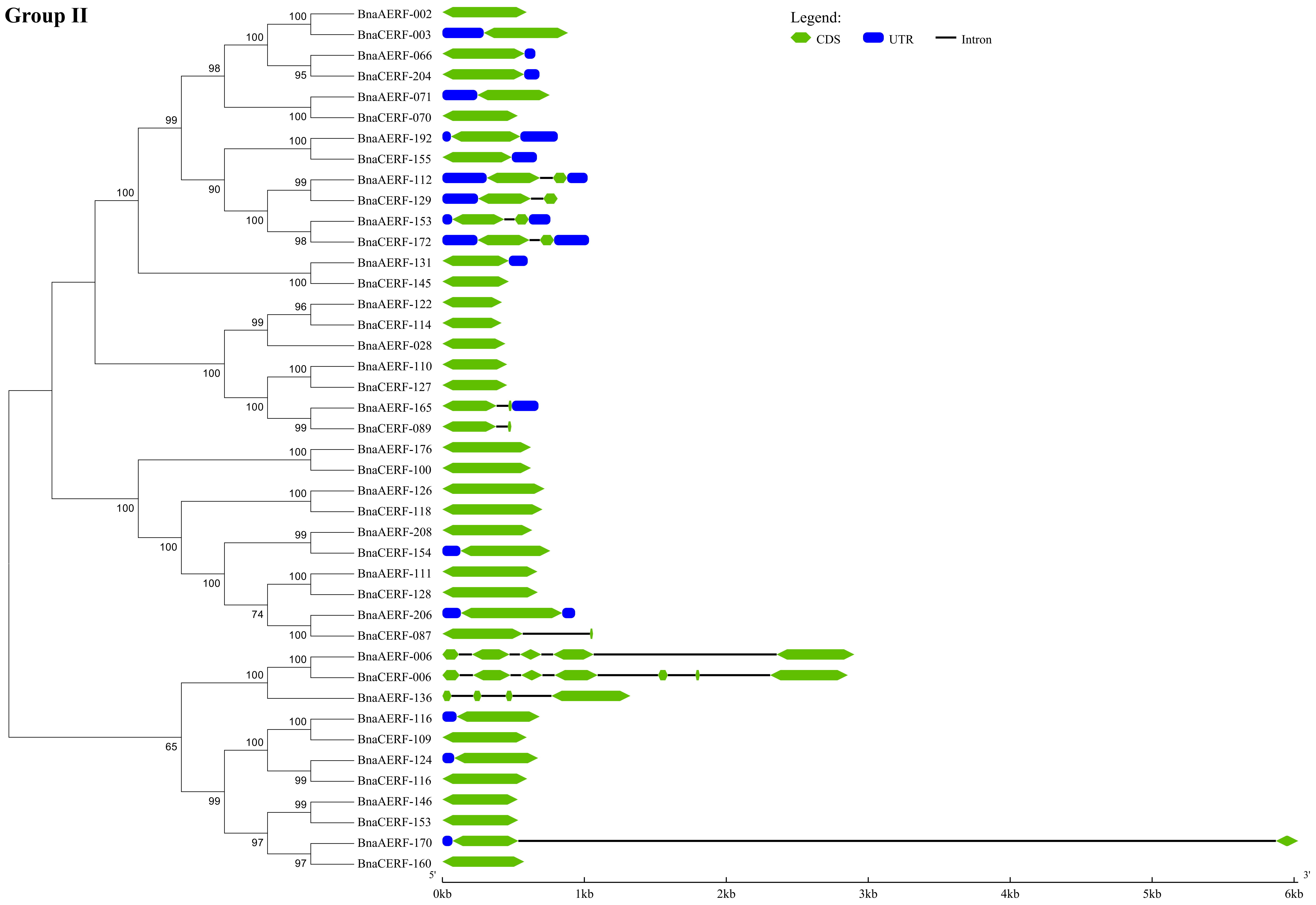
Group II
Legend:
CDS
UTR
Intron
BnaAERF-002
BnaCERF-003
BnaAERF-066
BnaCERF-204
BnaAERF-071
BnaCERF-070
BnaAERF-192
BnaCERF-155
BnaAERF-112
BnaCERF-129
BnaAERF-153
BnaCERF-172
BnaAERF-131
BnaCERF-145
BnaAERF-122
BnaCERF-114
BnaAERF-028
BnaAERF-110
BnaCERF-127
BnaAERF-165
BnaCERF-089
BnaAERF-176
BnaCERF-100
BnaAERF-126
BnaCERF-118
BnaAERF-208
BnaCERF-154
BnaAERF-111
BnaCERF-128
BnaAERF-206
BnaCERF-087
BnaAERF-006
BnaCERF-006
BnaAERF-136
BnaAERF-116
BnaCERF-109
BnaAERF-124
BnaCERF-116
BnaAERF-146
BnaCERF-153
BnaAERF-170
BnaCERF-160
5'
3'
0kb
1kb
2kb
3kb
4kb
5kb
6kb

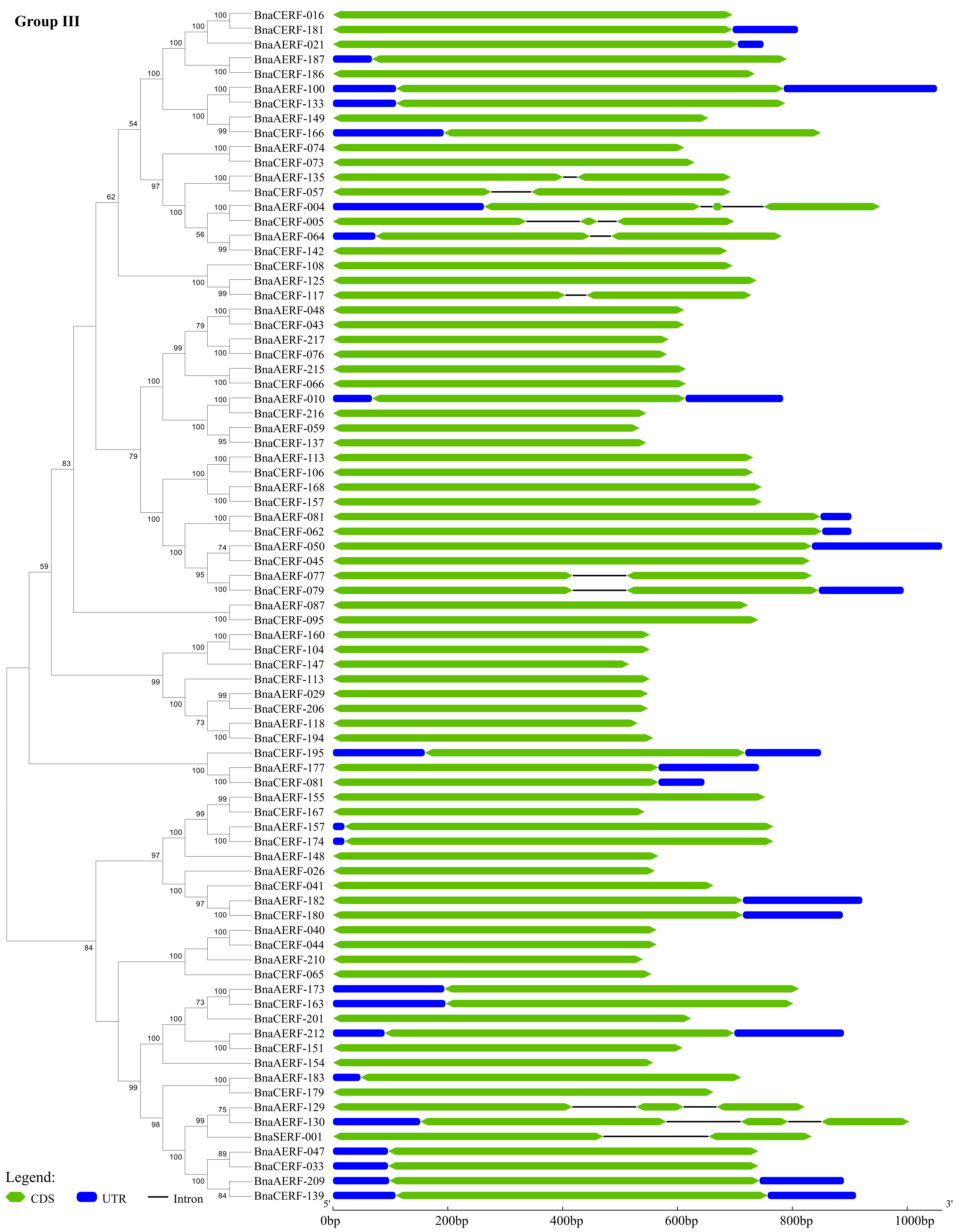

Group III
BnaCERF-016
BnaCERF-181
BnaAERF-021
BnaAERF-187
BnaCERF-186
BnaAERF-100
BnaCERF-133
BnaAERF-149
BnaCERF-166
BnaAERF-074
BnaCERF-073
BnaAERF-135
BnaCERF-057
BnaAERF-004
BnaCERF-005
BnaAERF-064
BnaCERF-142
BnaCERF-108
BnaAERF-125
BnaCERF-117
BnaAERF-048
BnaCERF-043
BnaAERF-217
BnaCERF-076
BnaAERF-215
BnaCERF-066
BnaAERF-010
BnaCERF-216
BnaAERF-059
BnaCERF-137
BnaAERF-113
BnaCERF-106
BnaAERF-168
BnaCERF-157
BnaAERF-081
BnaCERF-062
BnaAERF-050
BnaCERF-045
BnaAERF-077
BnaCERF-079
BnaAERF-087
BnaCERF-095
BnaAERF-160
BnaCERF-104
BnaCERF-147
BnaCERF-113
BnaAERF-029
BnaCERF-206
BnaAERF-118
BnaCERF-194
BnaCERF-195
BnaAERF-177
BnaCERF-081
BnaAERF-155
BnaCERF-167
BnaAERF-157
BnaCERF-174
BnaAERF-148
BnaAERF-026
BnaCERF-041
BnaAERF-182
BnaCERF-180
BnaAERF-040
BnaCERF-044
BnaAERF-210
BnaCERF-065
BnaAERF-173
BnaCERF-163
BnaCERF-201
BnaAERF-212
BnaCERF-151
BnaAERF-154
BnaAERF-183
BnaCERF-179
BnaAERF-129
BnaAERF-130
BnaSERF-001
BnaAERF-047
BnaCERF-033
BnaAERF-209
BnaCERF-139
Legend:
CDS
UTR
Intron
5'
3'
0bp
200bp
400bp
600bp
800bp
1000bp

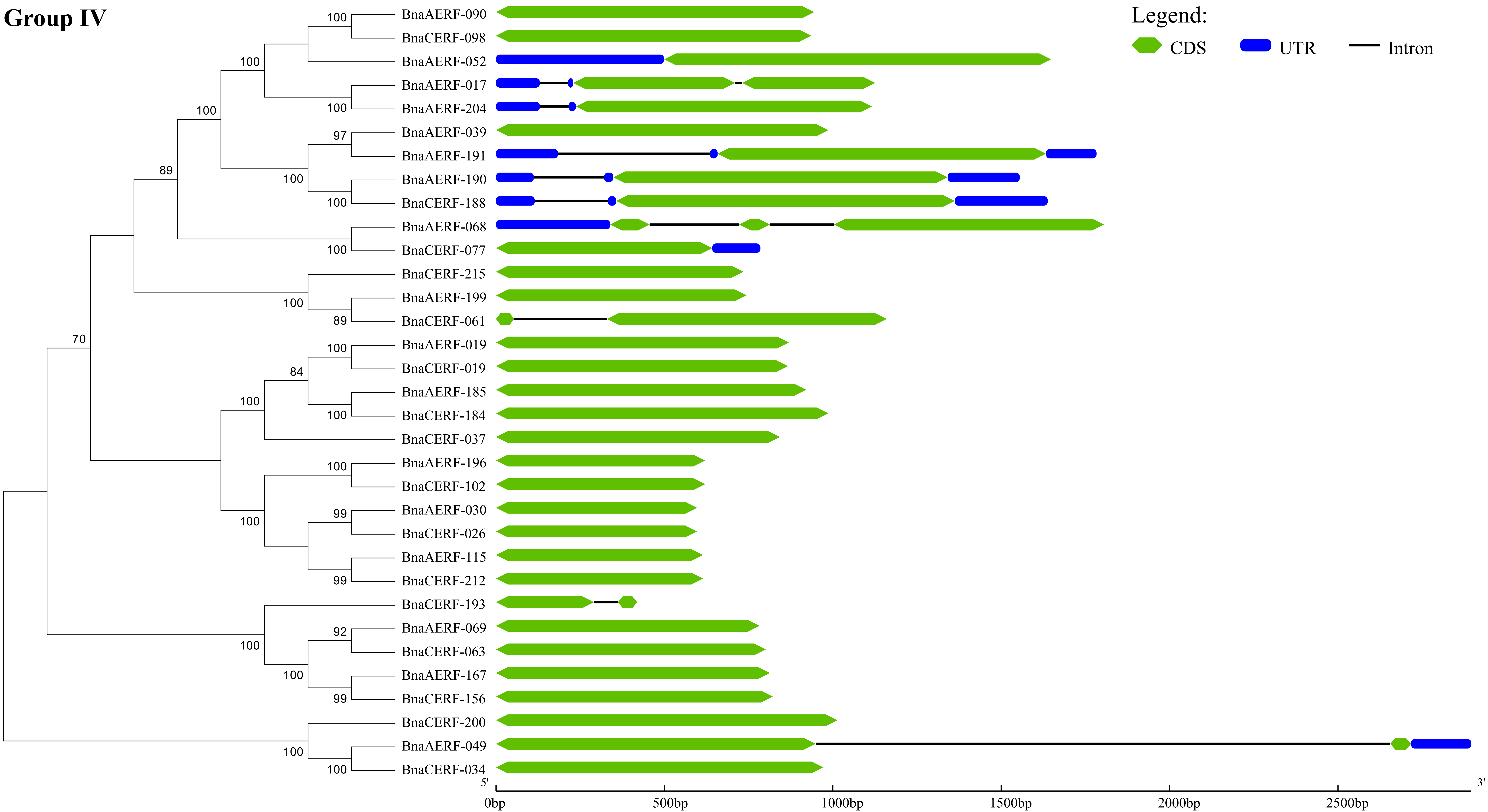

Group IV
Legend:
CDS
UTR
Intron
BnaAERF-090
BnaCERF-098
BnaAERF-052
BnaAERF-017
BnaAERF-204
BnaAERF-039
BnaAERF-191
BnaAERF-190
BnaCERF-188
BnaAERF-068
BnaCERF-077
BnaCERF-215
BnaAERF-199
BnaCERF-061
BnaAERF-019
BnaCERF-019
BnaAERF-185
BnaCERF-184
BnaCERF-037
BnaAERF-196
BnaCERF-102
BnaAERF-030
BnaCERF-026
BnaAERF-115
BnaCERF-212
BnaCERF-193
BnaAERF-069
BnaCERF-063
BnaAERF-167
BnaCERF-156
BnaCERF-200
BnaAERF-049
BnaCERF-034
5'
3'
0bp
500bp
1000bp
1500bp
2000bp
2500bp

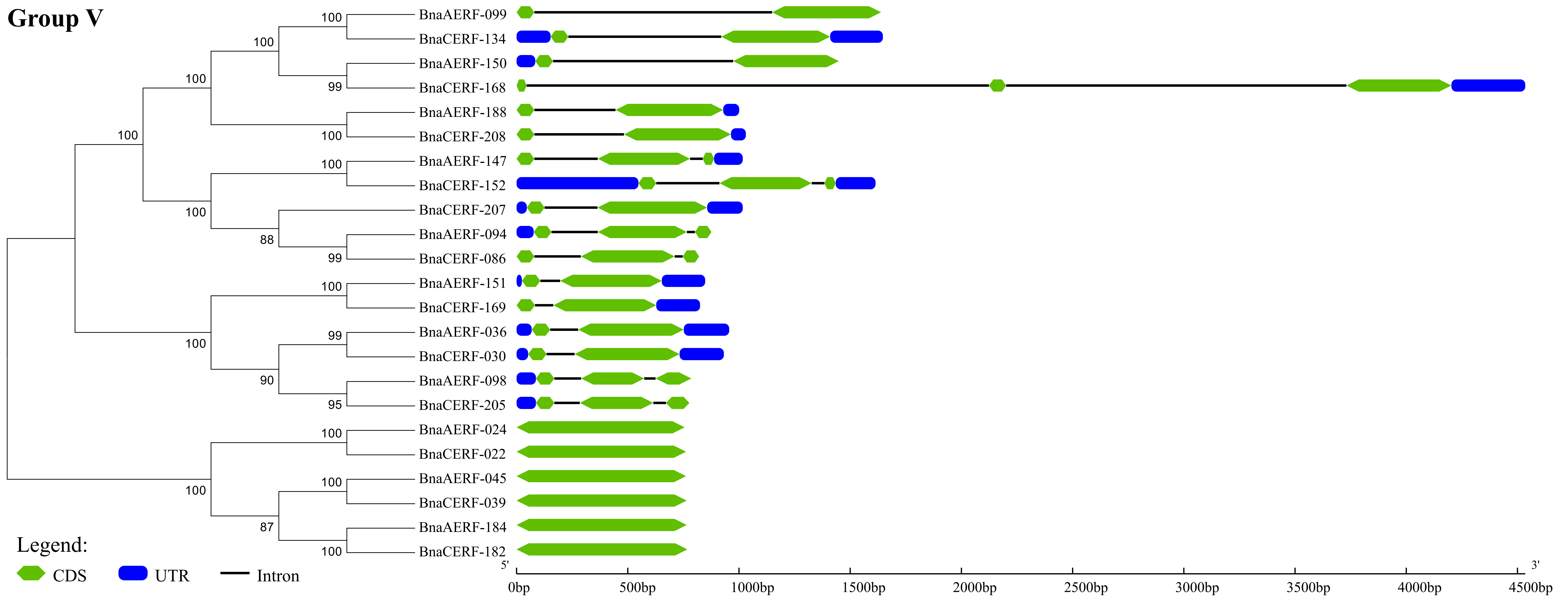

Group V
BnaAERF-099
BnaCERF-134
BnaAERF-150
BnaCERF-168
BnaAERF-188
BnaCERF-208
BnaAERF-147
BnaCERF-152
BnaCERF-207
BnaAERF-094
BnaCERF-086
BnaAERF-151
BnaCERF-169
BnaAERF-036
BnaCERF-030
BnaAERF-098
BnaCERF-205
BnaAERF-024
BnaCERF-022
BnaAERF-045
BnaCERF-039
BnaAERF-184
BnaCERF-182
Legend:
CDS
UTR
Intron
5'
3'
0bp
500bp
1000bp
1500bp
2000bp
2500bp
3000bp
3500bp
4000bp
4500bp

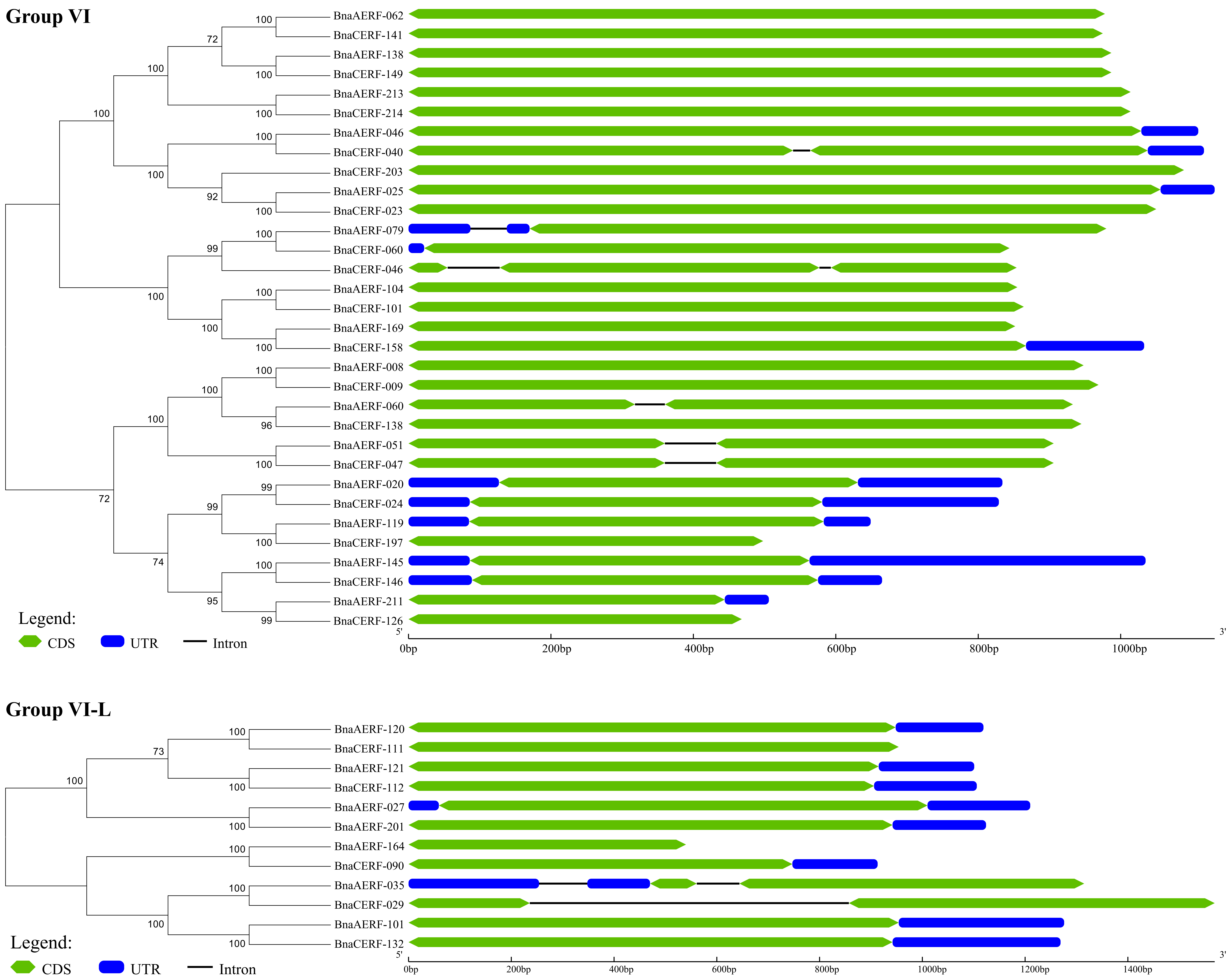
Group VI
BnaAERF-062
BnaCERF-141
BnaAERF-138
BnaCERF-149
BnaAERF-213
BnaCERF-214
BnaAERF-046
BnaCERF-040
BnaCERF-203
BnaAERF-025
BnaAERF-023
BnaAERF-079
BnaCERF-060
BnaCERF-046
BnaAERF-104
BnaCERF-101
BnaAERF-169
BnaCERF-158
BnaAERF-008
BnaCERF-009
BnaAERF-060
BnaCERF-138
BnaAERF-051
BnaCERF-047
BnaAERF-020
BnaCERF-024
BnaAERF-119
BnaCERF-197
BnaAERF-145
BnaCERF-146
BnaAERF-211
BnaCERF-126
Legend:
CDS
UTR
Intron
5'
3'
0bp
200bp
400bp
600bp
800bp
1000bp
Group VI-L
BnaAERF-120
BnaCERF-111
BnaAERF-121
BnaCERF-112
BnaAERF-027
BnaAERF-201
BnaAERF-164
BnaCERF-090
BnaAERF-035
BnaCERF-029
BnaAERF-101
BnaCERF-132
Legend:
CDS
UTR
Intron
5'
3'
0bp
200bp
400bp
600bp
800bp
1000bp
1200bp
1400bp

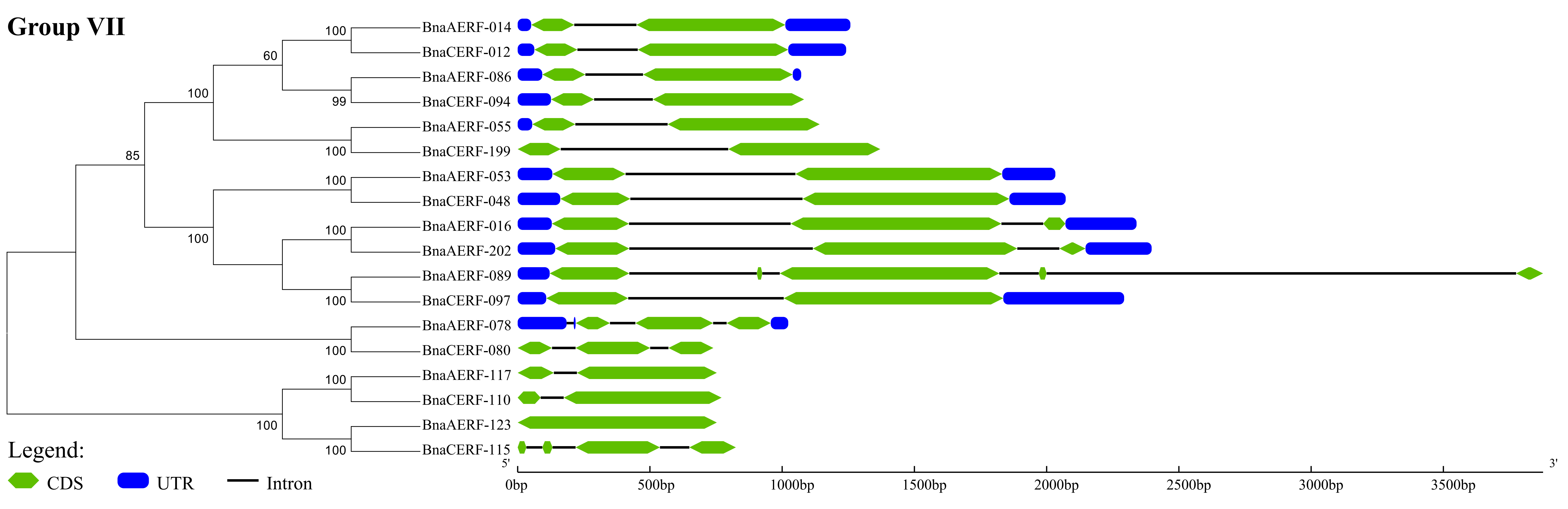

Group VII
BnaAERF-014
BnaCERF-012
BnaAERF-086
BnaCERF-094
BnaAERF-055
BnaCERF-199
BnaAERF-053
BnaCERF-048
BnaAERF-016
BnaAERF-202
BnaAERF-089
BnaCERF-097
BnaAERF-078
BnaCERF-080
BnaAERF-117
BnaCERF-110
BnaAERF-123
BnaCERF-115
100
60
100
99
100
85
100
100
100
100
100
100
100
100
100
Legend:
CDS
UTR
Intron
5'
3'
0bp
500bp
1000bp
1500bp
2000bp
2500bp
3000bp
3500bp

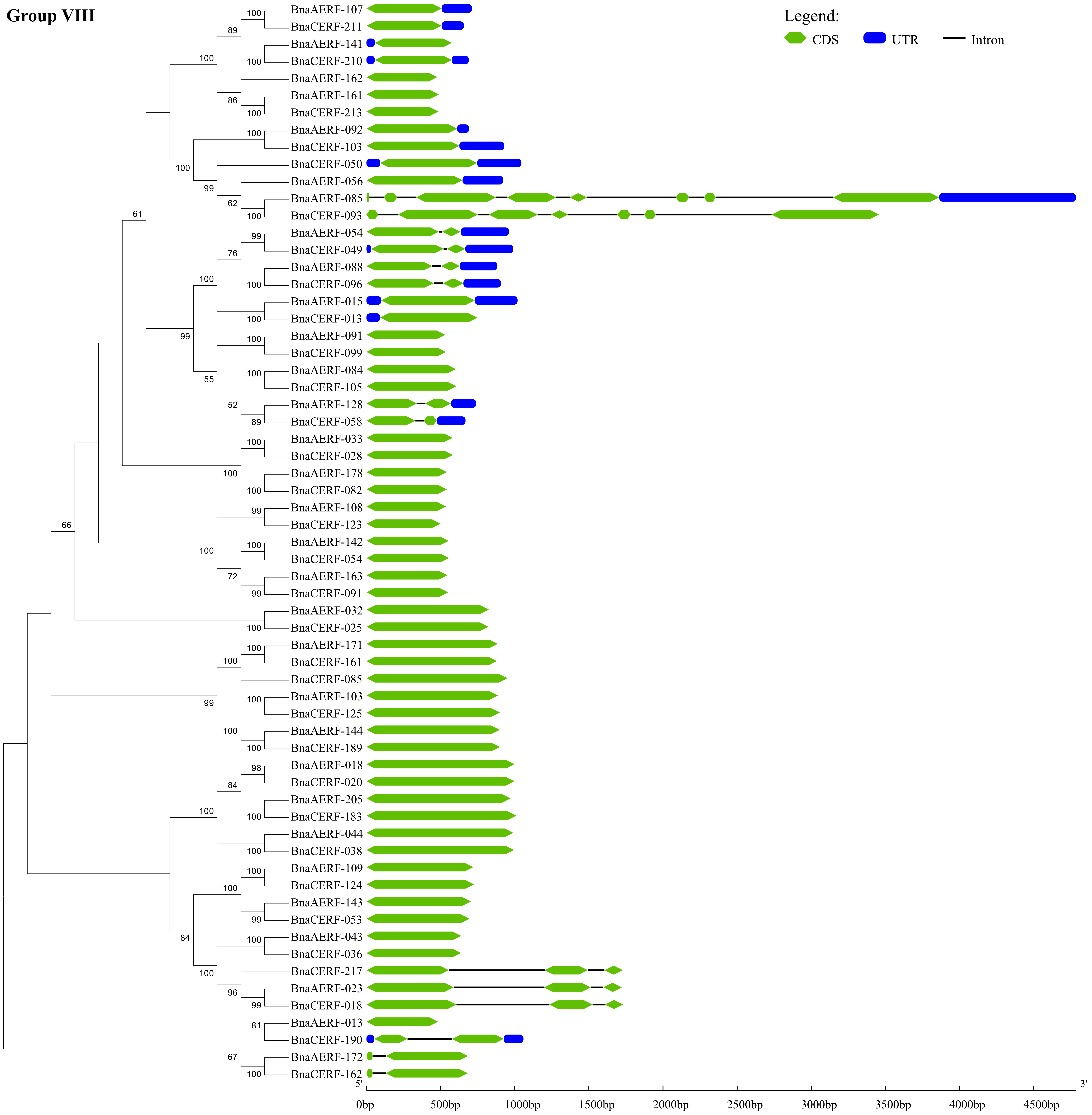

Group VIII
Legend:
CDS
UTR
Intron
BnaAERF-107
BnaCERF-211
BnaAERF-141
BnaCERF-210
BnaAERF-162
BnaAERF-161
BnaCERF-213
BnaAERF-092
BnaCERF-103
BnaCERF-050
BnaCERF-056
BnaAERF-085
BnaCERF-093
BnaAERF-054
BnaCERF-049
BnaAERF-088
BnaCERF-096
BnaAERF-015
BnaCERF-013
BnaAERF-091
BnaCERF-099
BnaAERF-084
BnaCERF-105
BnaAERF-128
BnaCERF-058
BnaAERF-033
BnaCERF-028
BnaAERF-178
BnaCERF-082
BnaAERF-108
BnaCERF-123
BnaAERF-142
BnaCERF-054
BnaAERF-163
BnaCERF-091
BnaAERF-032
BnaCERF-025
BnaAERF-171
BnaCERF-161
BnaCERF-085
BnaAERF-103
BnaCERF-125
BnaAERF-144
BnaCERF-189
BnaAERF-018
BnaCERF-020
BnaAERF-205
BnaCERF-183
BnaAERF-044
BnaCERF-038
BnaAERF-109
BnaCERF-124
BnaAERF-143
BnaCERF-053
BnaAERF-043
BnaCERF-036
BnaCERF-217
BnaCERF-023
BnaCERF-018
BnaAERF-013
BnaCERF-190
BnaAERF-172
BnaCERF-162
5'
3'
0bp
500bp
1000bp
1500bp
2000bp
2500bp
3000bp
3500bp
4000bp
4500bp

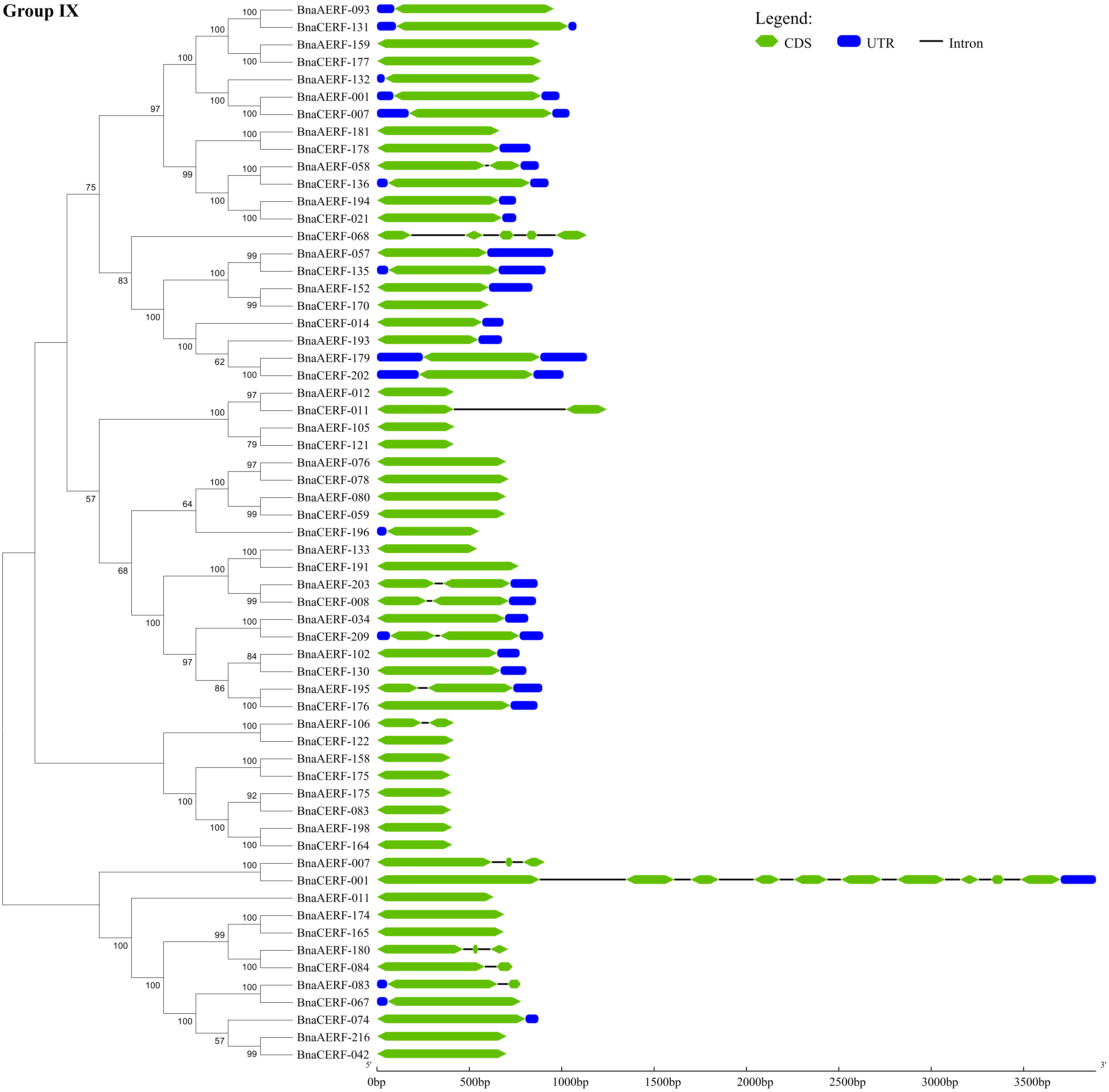
Group IX
Legend:
CDS
UTR
Intron
BnaAERF-093
BnaCERF-131
BnaAERF-159
BnaCERF-177
BnaAERF-132
BnaAERF-001
BnaCERF-007
BnaAERF-181
BnaCERF-178
BnaAERF-058
BnaCERF-136
BnaAERF-194
BnaCERF-021
BnaCERF-068
BnaAERF-057
BnaCERF-135
BnaAERF-152
BnaCERF-170
BnaCERF-014
BnaAERF-193
BnaAERF-179
BnaCERF-202
BnaAERF-012
BnaCERF-011
BnaAERF-105
BnaCERF-121
BnaAERF-076
BnaCERF-078
BnaAERF-080
BnaCERF-059
BnaCERF-196
BnaAERF-133
BnaCERF-191
BnaAERF-203
BnaCERF-008
BnaAERF-034
BnaCERF-209
BnaAERF-102
BnaCERF-130
BnaAERF-195
BnaCERF-176
BnaAERF-106
BnaCERF-122
BnaAERF-158
BnaCERF-175
BnaAERF-175
BnaCERF-083
BnaAERF-198
BnaCERF-164
BnaAERF-007
BnaCERF-001
BnaAERF-011
BnaAERF-174
BnaCERF-165
BnaAERF-180
BnaCERF-084
BnaAERF-083
BnaCERF-067
BnaCERF-074
BnaAERF-216
BnaCERF-042
5'
3'
0bp
500bp
1000bp
1500bp
2000bp
2500bp
3000bp
3500bp

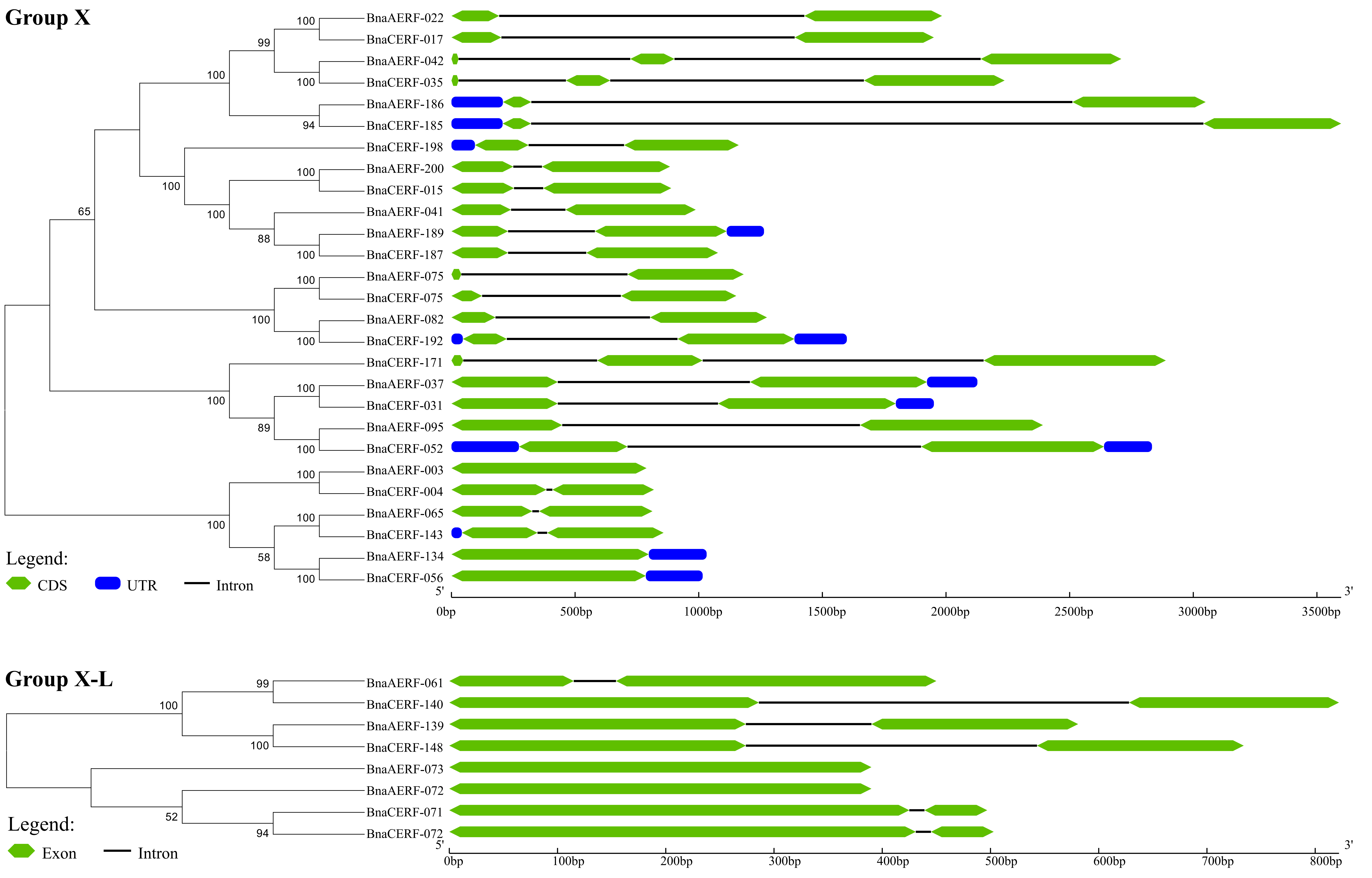
Group X
BnaAERF-022
BnaCERF-017
BnaAERF-042
BnaCERF-035
BnaAERF-186
BnaCERF-185
BnaCERF-198
BnaAERF-200
BnaCERF-015
BnaAERF-041
BnaAERF-189
BnaCERF-187
BnaAERF-075
BnaCERF-075
BnaAERF-082
BnaCERF-192
BnaCERF-171
BnaAERF-037
BnaCERF-031
BnaAERF-095
BnaCERF-052
BnaAERF-003
BnaCERF-004
BnaAERF-065
BnaCERF-143
BnaAERF-134
BnaCERF-056
Legend:
CDS
UTR
Intron
5'
3'
0bp
500bp
1000bp
1500bp
2000bp
2500bp
3000bp
3500bp
Group X-L
BnaAERF-061
BnaCERF-140
BnaAERF-139
BnaCERF-148
BnaAERF-073
BnaAERF-072
BnaCERF-071
BnaCERF-072
Legend:
Exon
Intron
0bp
100bp
200bp
300bp
400bp
500bp
600bp
700bp
800bp

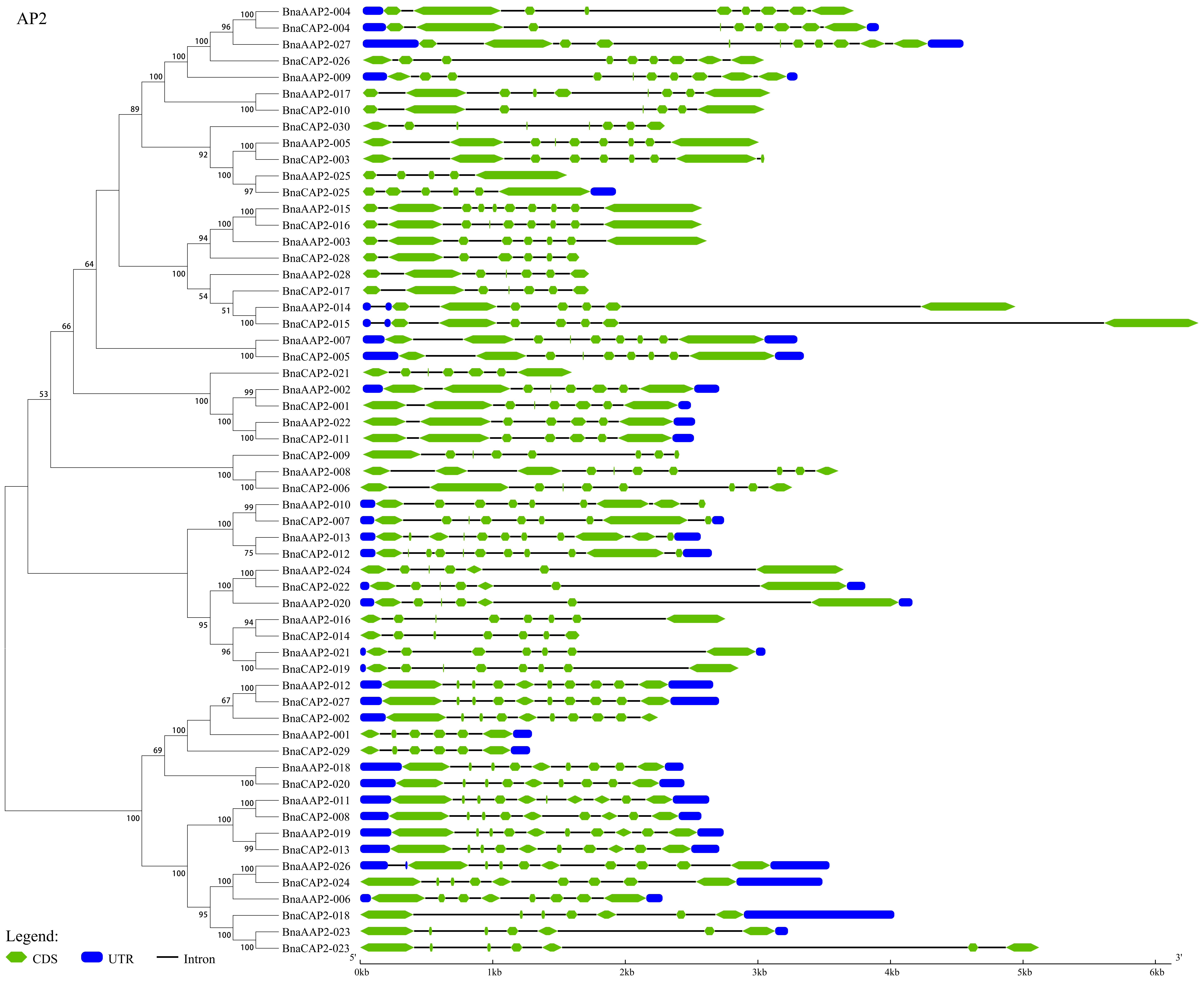

AP2
BnaAAP2-004
BnaCAP2-004
BnaAAP2-027
BnaCAP2-026
BnaAAP2-009
BnaAAP2-017
BnaCAP2-010
BnaCAP2-030
BnaAAP2-005
BnaCAP2-003
BnaAAP2-025
BnaCAP2-025
BnaAAP2-015
BnaCAP2-016
BnaAAP2-003
BnaCAP2-028
BnaAAP2-028
BnaCAP2-017
BnaAAP2-014
BnaCAP2-015
BnaAAP2-007
BnaCAP2-005
BnaCAP2-021
BnaAAP2-002
BnaCAP2-001
BnaAAP2-022
BnaCAP2-011
BnaCAP2-009
BnaAAP2-008
BnaCAP2-006
BnaAAP2-010
BnaCAP2-007
BnaAAP2-013
BnaCAP2-012
BnaAAP2-024
BnaCAP2-022
BnaAAP2-020
BnaAAP2-016
BnaCAP2-014
BnaAAP2-021
BnaCAP2-019
BnaAAP2-012
BnaCAP2-027
BnaCAP2-002
BnaAAP2-001
BnaCAP2-029
BnaAAP2-018
BnaCAP2-020
BnaAAP2-011
BnaCAP2-008
BnaAAP2-019
BnaCAP2-013
BnaAAP2-026
BnaCAP2-024
BnaAAP2-006
BnaCAP2-018
BnaAAP2-023
BnaCAP2-023
Legend:
CDS
UTR
Intron
5'
3'
0kb
1kb
2kb
3kb
4kb
5kb
6kb

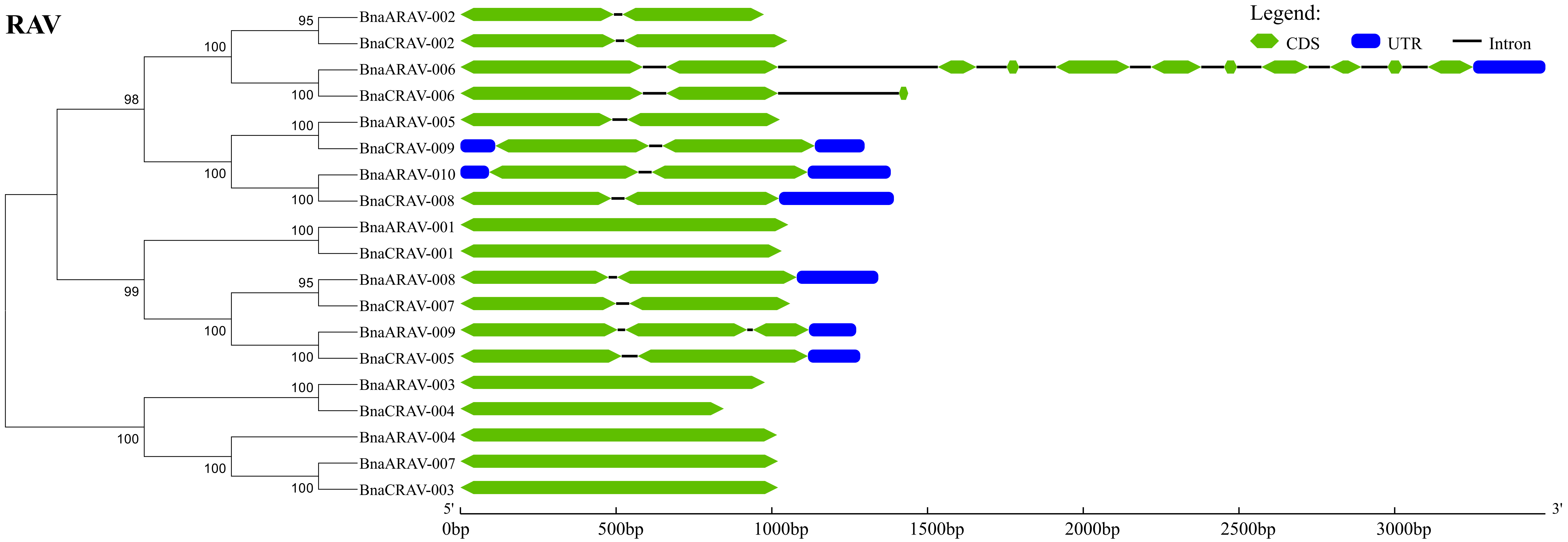

RAV
Legend:
CDS
UTR
Intron
BnaARAV-002
BnaCRAV-002
BnaARAV-006
BnaCRAV-006
BnaARAV-005
BnaCRAV-009
BnaARAV-010
BnaCRAV-008
BnaARAV-001
BnaCRAV-001
BnaARAV-008
BnaCRAV-007
BnaARAV-009
BnaCRAV-005
BnaARAV-003
BnaCRAV-004
BnaARAV-004
BnaARAV-007
BnaCRAV-003
95
100
98
100
100
100
100
100
100
99
95
100
100
100
100
100
100
5'
3'
0bp
500bp
1000bp
1500bp
2000bp
2500bp
3000bp

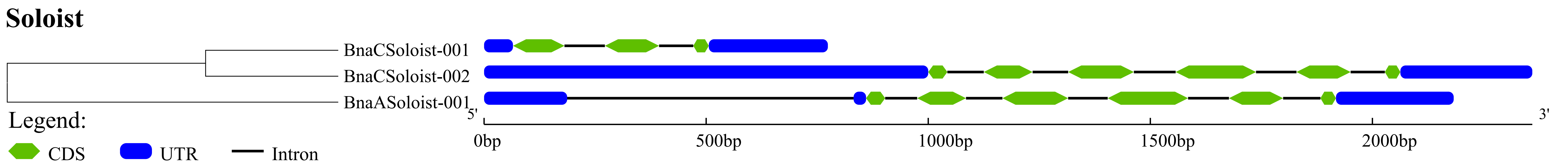

Soloist
BnaCSoloist-001
BnaCSoloist-002
BnaASoloist-001
5'
3'
0bp
500bp
1000bp
1500bp
2000bp
Legend:
CDS
UTR
Intron
