# Supplementary material for: Origination, Expansion, Evolutionary Trajectory, and Expression Bias of AP2/ERF Superfamily in Brassica napus
Source: Front Plant Sci. 2016 Aug 12;7:1186. doi: 10.3389/fpls.2016.01186 (PMC4982375; doi:10.3389/fpls.2016.01186)
Supplement: Supplementary file 4 [file DataSheet3.PDF]

Figure S12. The Heat map representation and hierarchical clustering of genes in root and leaf for each AP2/ERF group. The expression level of these genes in B. napus obtained from the transcriptome data, which contained three replicates for leaf and root. The expression values were calculated by RPKM (Reads Per Kilobase per Million mapped reads), and then conducted log2 transformed.

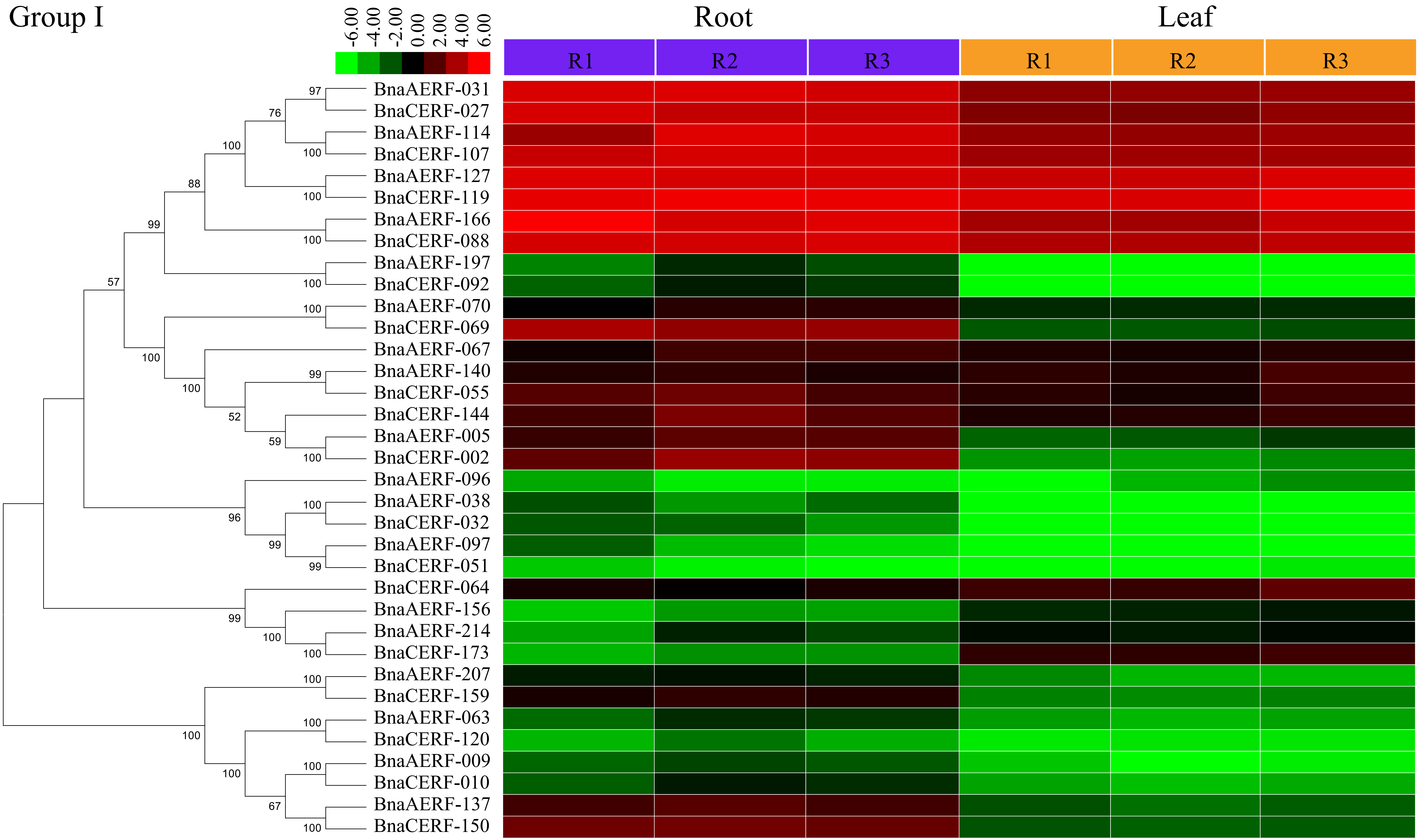

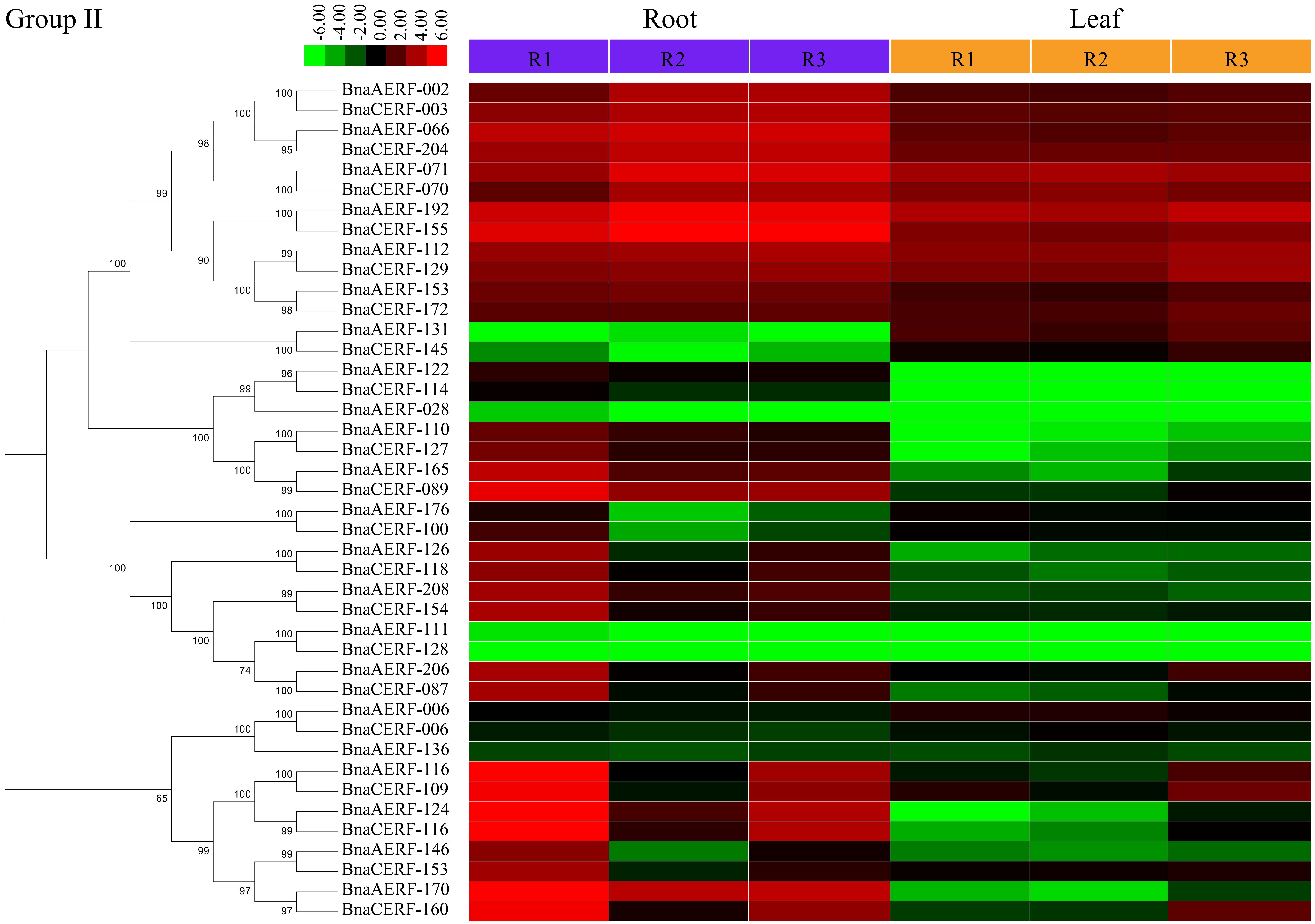

Group II
-6.00
-4.00
-2.00
0.00
2.00
4.00
6.00
Root
Leaf
R1
R2
R3
R1
R2
R3
BnaAERF-002
BnaCERF-003
BnaAERF-066
BnaCERF-204
BnaAERF-071
BnaCERF-070
BnaAERF-192
BnaCERF-155
BnaAERF-112
BnaCERF-129
BnaAERF-153
BnaCERF-172
BnaAERF-131
BnaCERF-145
BnaAERF-122
BnaCERF-114
BnaAERF-028
BnaAERF-110
BnaCERF-127
BnaAERF-165
BnaCERF-089
BnaAERF-176
BnaCERF-100
BnaAERF-126
BnaCERF-118
BnaAERF-208
BnaCERF-154
BnaAERF-111
BnaCERF-128
BnaAERF-206
BnaCERF-087
BnaAERF-006
BnaCERF-006
BnaAERF-136
BnaAERF-116
BnaCERF-109
BnaAERF-124
BnaCERF-116
BnaAERF-146
BnaCERF-153
BnaAERF-170
BnaCERF-160

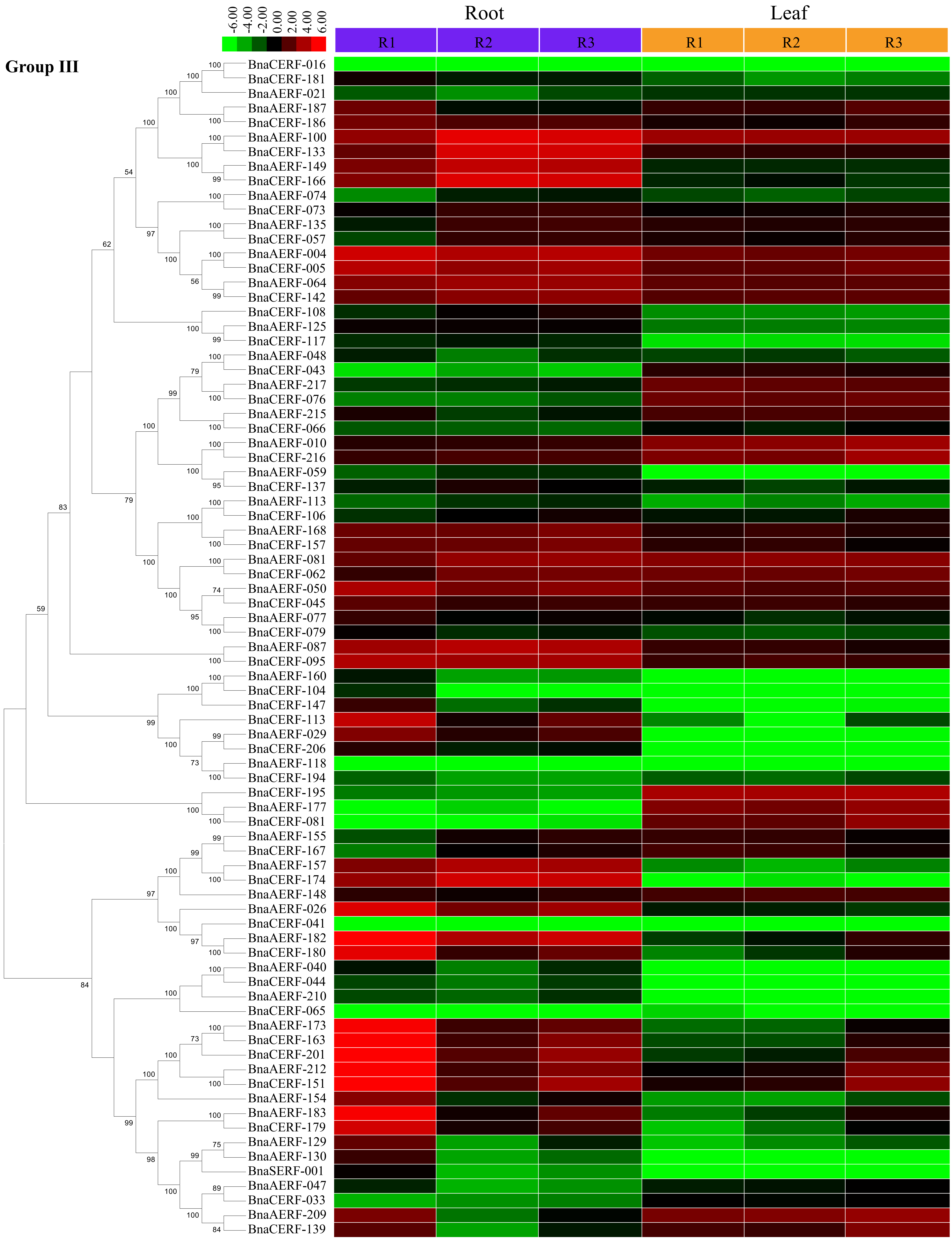

Group III
-6.00
-4.00
-2.00
0.00
2.00
4.00
6.00
Root
Leaf
R1
R2
R3
R1
R2
R3
BnaCERF-016
BnaCERF-181
BnaAERF-021
BnaAERF-187
BnaCERF-186
BnaAERF-100
BnaCERF-133
BnaAERF-149
BnaCERF-166
BnaAERF-074
BnaCERF-073
BnaAERF-135
BnaCERF-057
BnaAERF-004
BnaCERF-005
BnaAERF-064
BnaCERF-142
BnaCERF-108
BnaAERF-125
BnaCERF-117
BnaAERF-048
BnaCERF-043
BnaAERF-217
BnaCERF-076
BnaAERF-215
BnaCERF-066
BnaAERF-010
BnaCERF-216
BnaAERF-059
BnaCERF-137
BnaAERF-113
BnaCERF-106
BnaAERF-168
BnaCERF-157
BnaAERF-081
BnaCERF-062
BnaAERF-050
BnaCERF-045
BnaAERF-077
BnaCERF-079
BnaAERF-087
BnaCERF-095
BnaAERF-160
BnaCERF-104
BnaCERF-147
BnaCERF-113
BnaAERF-029
BnaCERF-206
BnaAERF-118
BnaCERF-194
BnaCERF-195
BnaAERF-177
BnaCERF-081
BnaAERF-155
BnaCERF-167
BnaAERF-157
BnaCERF-174
BnaAERF-148
BnaAERF-026
BnaCERF-041
BnaAERF-182
BnaCERF-180
BnaAERF-040
BnaCERF-044
BnaAERF-210
BnaCERF-065
BnaAERF-173
BnaCERF-163
BnaCERF-201
BnaAERF-212
BnaCERF-151
BnaAERF-154
BnaAERF-183
BnaCERF-179
BnaAERF-129
BnaAERF-130
BnaSERF-001
BnaAERF-047
BnaCERF-033
BnaAERF-209
BnaCERF-139

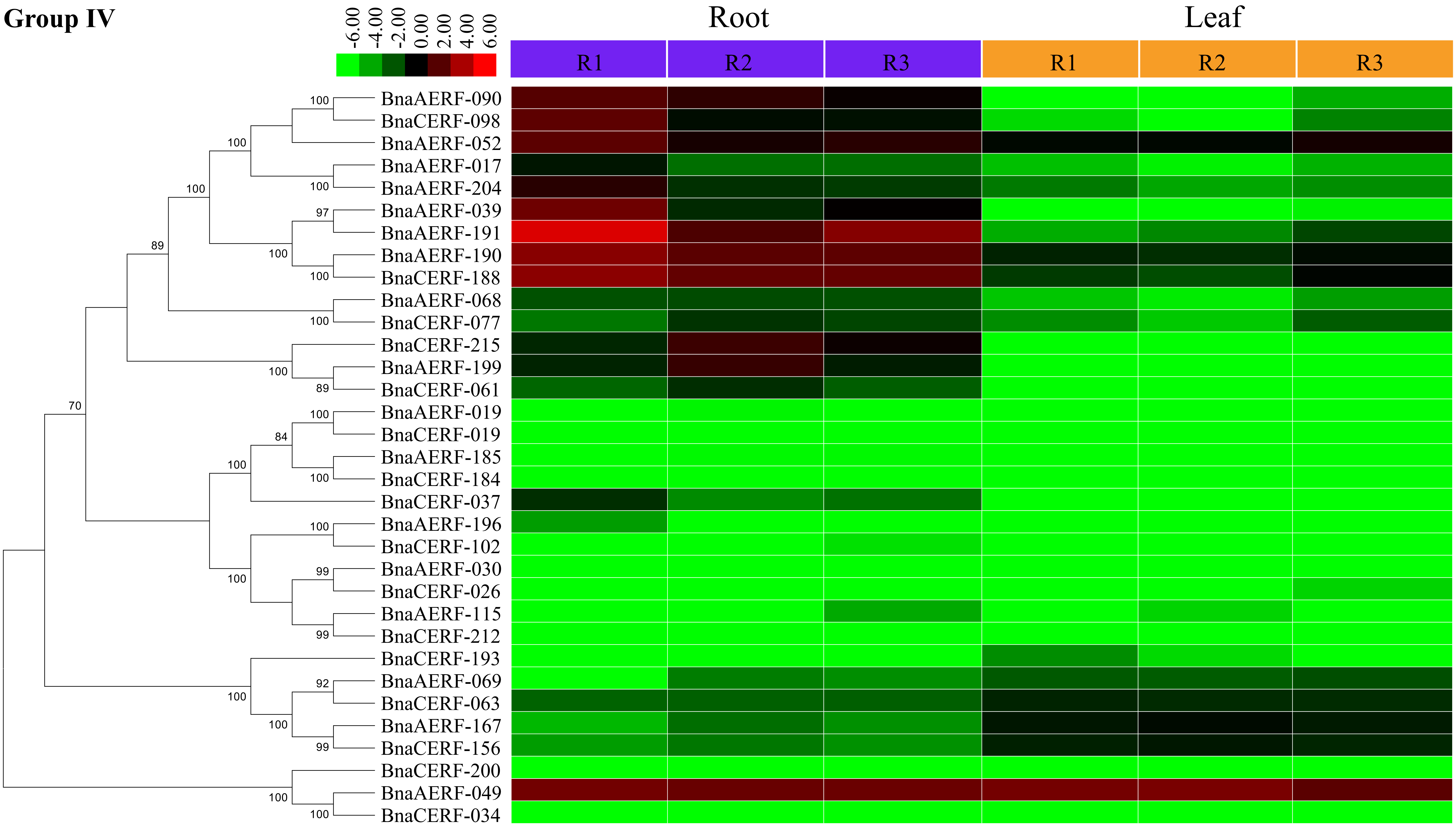
Group IV
-6.00 -4.00 -2.00 0.00 2.00 4.00 6.00
Root
Leaf
R1
R2
R3
R1
R2
R3
BnaAERF-090
BnaCERF-098
BnaAERF-052
BnaAERF-017
BnaAERF-204
BnaAERF-039
BnaAERF-191
BnaAERF-190
BnaCERF-188
BnaAERF-068
BnaCERF-077
BnaCERF-215
BnaAERF-199
BnaCERF-061
BnaAERF-019
BnaCERF-019
BnaAERF-185
BnaCERF-184
BnaCERF-037
BnaAERF-196
BnaCERF-102
BnaAERF-030
BnaCERF-026
BnaAERF-115
BnaCERF-212
BnaCERF-193
BnaAERF-069
BnaCERF-063
BnaAERF-167
BnaCERF-156
BnaCERF-200
BnaAERF-049
BnaCERF-034

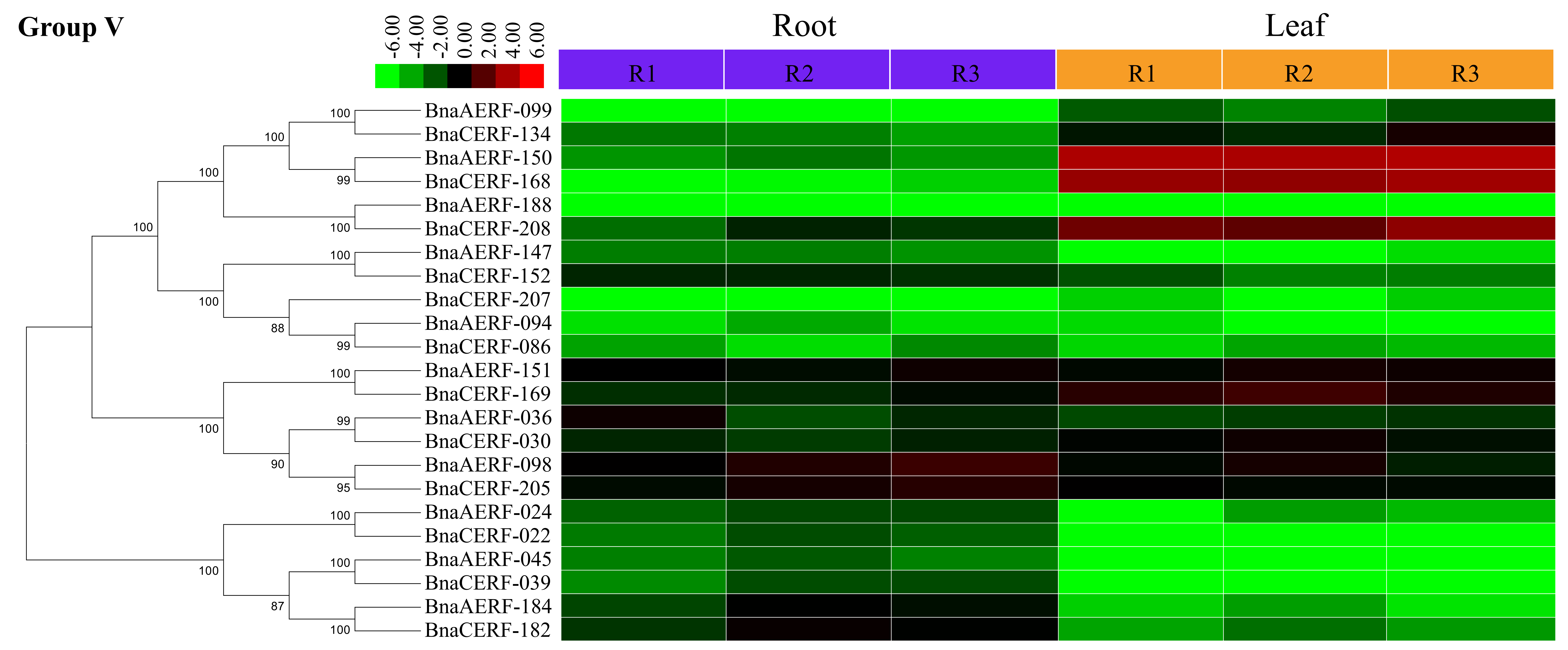
Group V
-6.00
-4.00
-2.00
0.00
2.00
4.00
6.00
Root
Leaf
R1
R2
R3
R1
R2
R3
BnaAERF-099
BnaCERF-134
BnaAERF-150
BnaCERF-168
BnaAERF-188
BnaCERF-208
BnaAERF-147
BnaCERF-152
BnaCERF-207
BnaAERF-094
BnaCERF-086
BnaAERF-151
BnaCERF-169
BnaAERF-036
BnaCERF-030
BnaAERF-098
BnaCERF-205
BnaAERF-024
BnaCERF-022
BnaAERF-045
BnaCERF-039
BnaAERF-184
BnaCERF-182

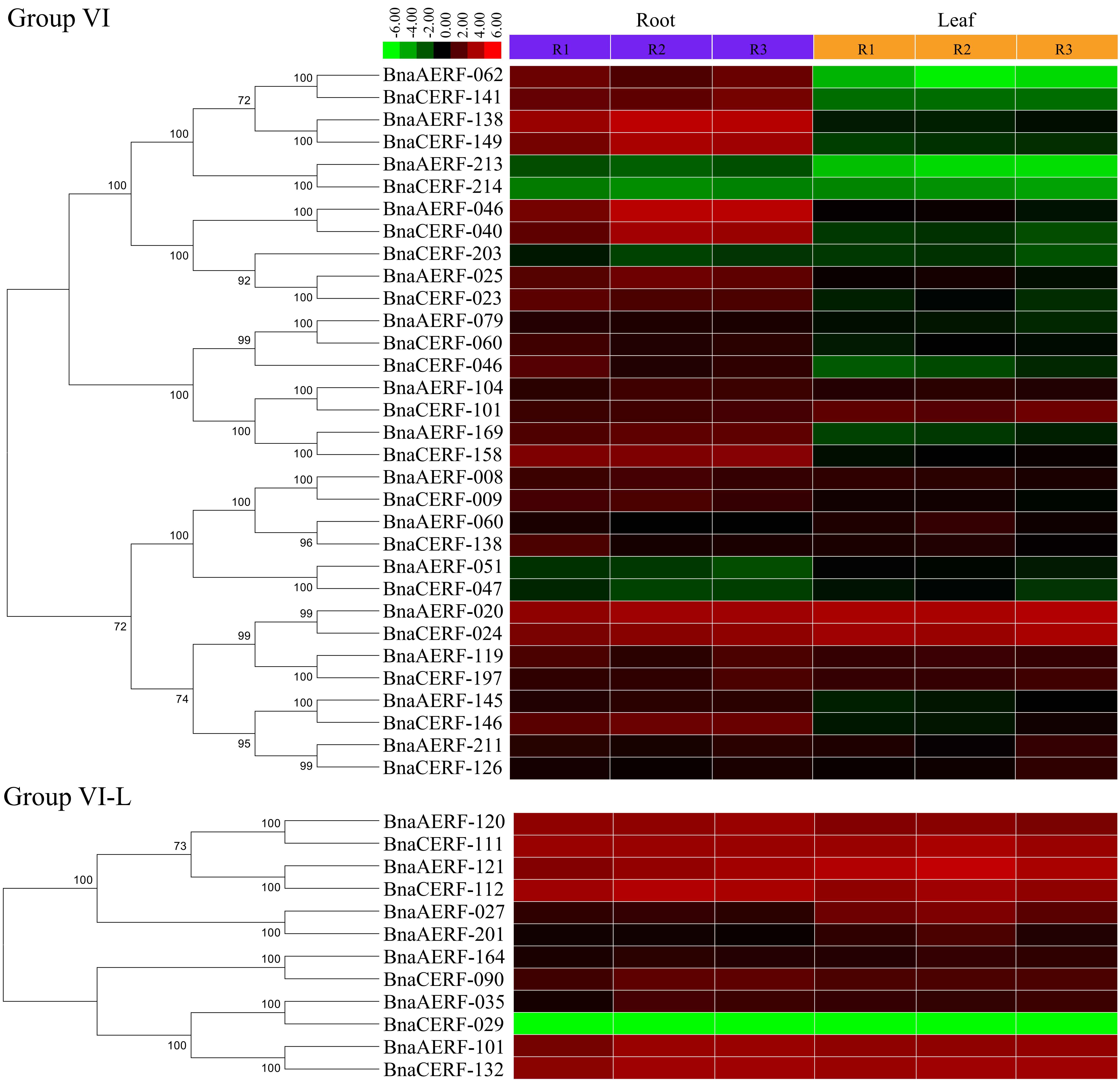
Group VI
-6.00
-4.00
-2.00
0.00
2.00
4.00
6.00
Root
Leaf
R1
R2
R3
R1
R2
R3
BnaAERF-062
BnaCERF-141
BnaAERF-138
BnaCERF-149
BnaAERF-213
BnaCERF-214
BnaAERF-046
BnaCERF-040
BnaCERF-203
BnaAERF-025
BnaCERF-023
BnaAERF-079
BnaCERF-060
BnaCERF-046
BnaAERF-104
BnaCERF-101
BnaAERF-169
BnaCERF-158
BnaAERF-008
BnaCERF-009
BnaAERF-060
BnaCERF-138
BnaAERF-051
BnaCERF-047
BnaAERF-020
BnaCERF-024
BnaAERF-119
BnaCERF-197
BnaAERF-145
BnaCERF-146
BnaAERF-211
BnaCERF-126
Group VI-L
BnaAERF-120
BnaCERF-111
BnaAERF-121
BnaCERF-112
BnaAERF-027
BnaAERF-201
BnaAERF-164
BnaCERF-090
BnaAERF-035
BnaCERF-029
BnaAERF-101
BnaCERF-132

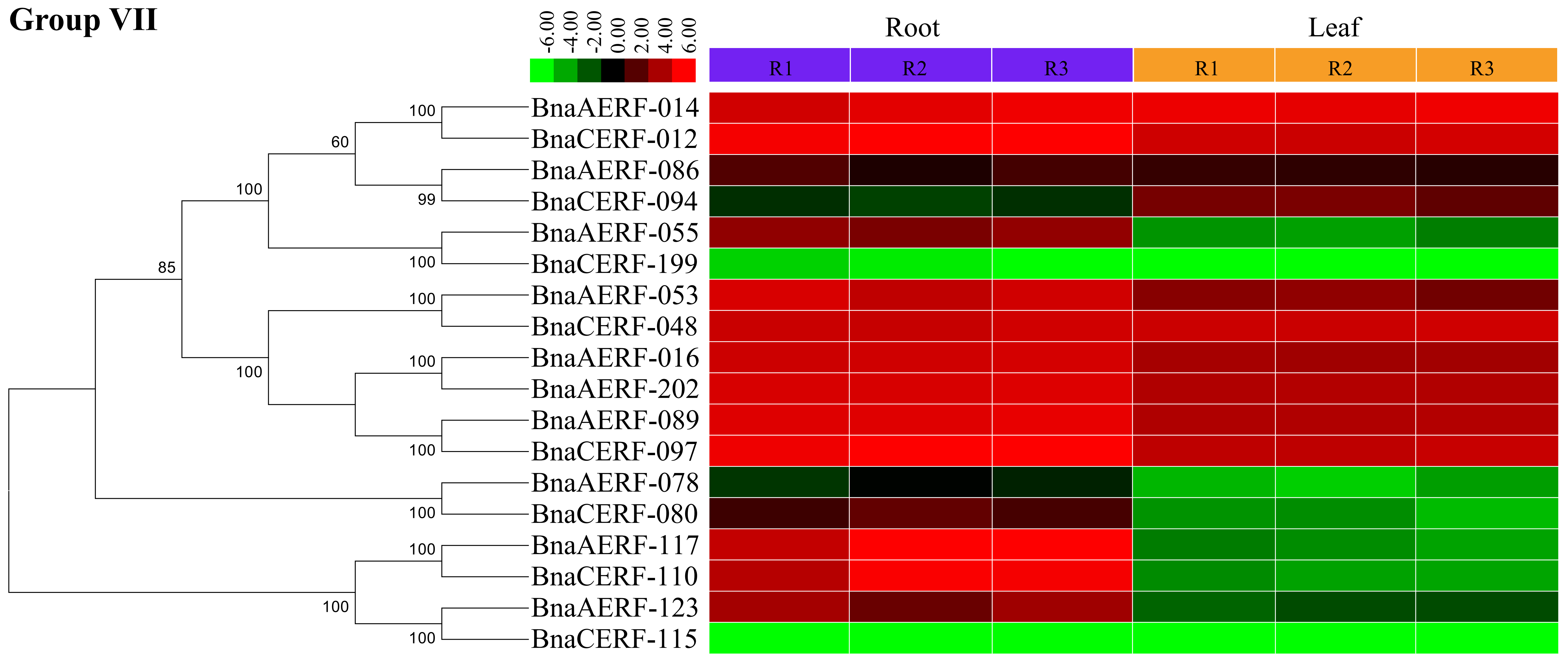
Group VII
-6.00
-4.00
-2.00
0.00
2.00
4.00
6.00
Root
Leaf
R1
R2
R3
R1
R2
R3
BnaAERF-014
BnaCERF-012
BnaAERF-086
BnaCERF-094
BnaAERF-055
BnaCERF-199
BnaAERF-053
BnaCERF-048
BnaAERF-016
BnaAERF-202
BnaAERF-089
BnaCERF-097
BnaAERF-078
BnaCERF-080
BnaAERF-117
BnaCERF-110
BnaAERF-123
BnaCERF-115
100
60
99
100
100
85
100
100
100
100
100
100
100
100
100

Group VIII

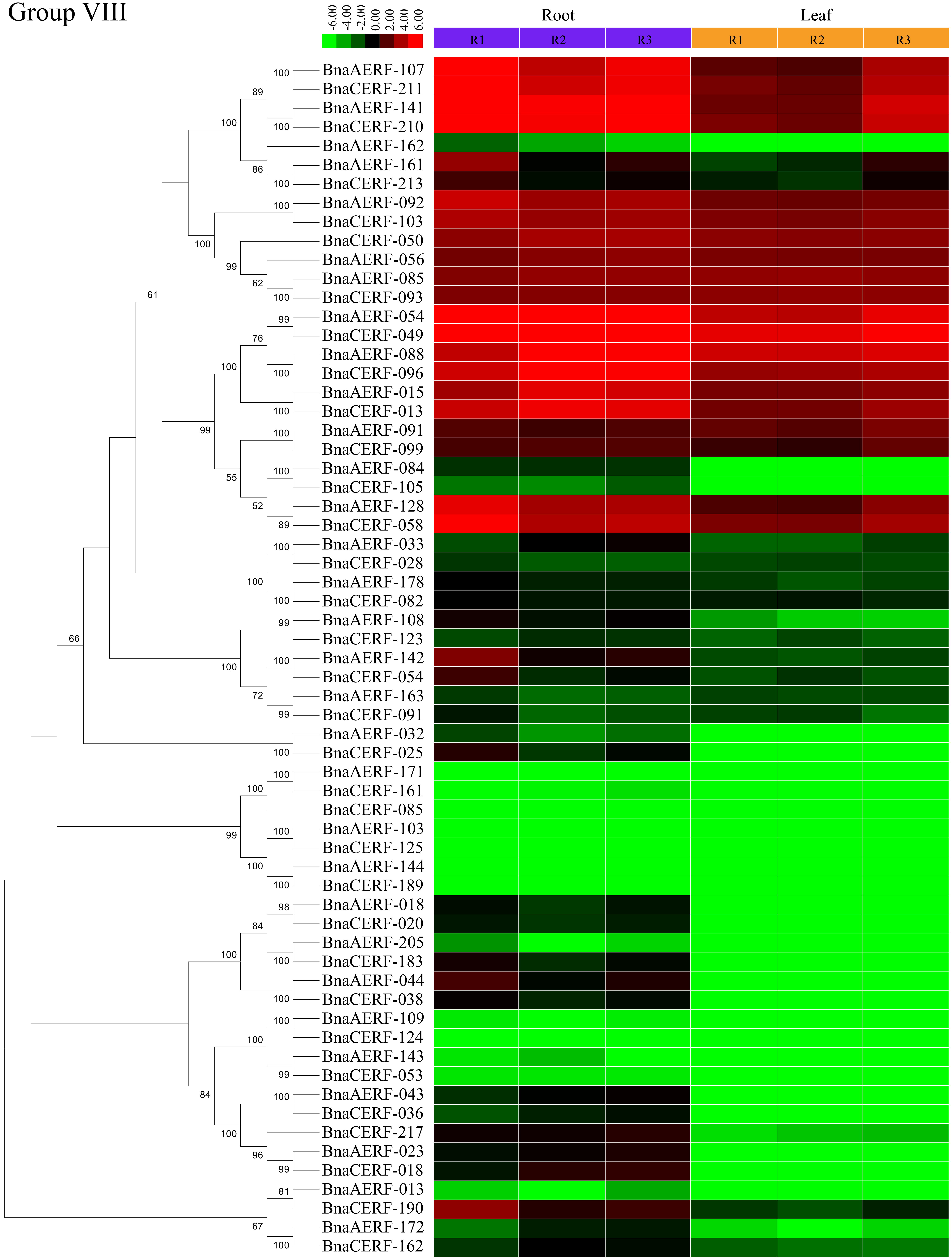

-6.00 -4.00 -2.00 0.00 2.00 4.00 6.00
Root
Leaf
R1
R2
R3
R1
R2
R3
BnaAERF-107
BnaCERF-211
BnaAERF-141
BnaCERF-210
BnaAERF-162
BnaAERF-161
BnaCERF-213
BnaAERF-092
BnaCERF-103
BnaCERF-050
BnaAERF-056
BnaAERF-085
BnaCERF-093
BnaAERF-054
BnaCERF-049
BnaAERF-088
BnaCERF-096
BnaAERF-015
BnaCERF-013
BnaAERF-091
BnaCERF-099
BnaAERF-084
BnaCERF-105
BnaAERF-128
BnaCERF-058
BnaAERF-033
BnaCERF-028
BnaAERF-178
BnaCERF-082
BnaAERF-108
BnaCERF-123
BnaAERF-142
BnaCERF-054
BnaAERF-163
BnaCERF-091
BnaAERF-032
BnaCERF-025
BnaAERF-171
BnaCERF-161
BnaCERF-085
BnaAERF-103
BnaCERF-125
BnaAERF-144
BnaCERF-189
BnaAERF-018
BnaCERF-020
BnaAERF-205
BnaCERF-183
BnaAERF-044
BnaCERF-038
BnaAERF-109
BnaCERF-124
BnaAERF-143
BnaCERF-053
BnaAERF-043
BnaCERF-036
BnaCERF-217
BnaAERF-023
BnaCERF-018
BnaAERF-013
BnaCERF-190
BnaAERF-172
BnaCERF-162

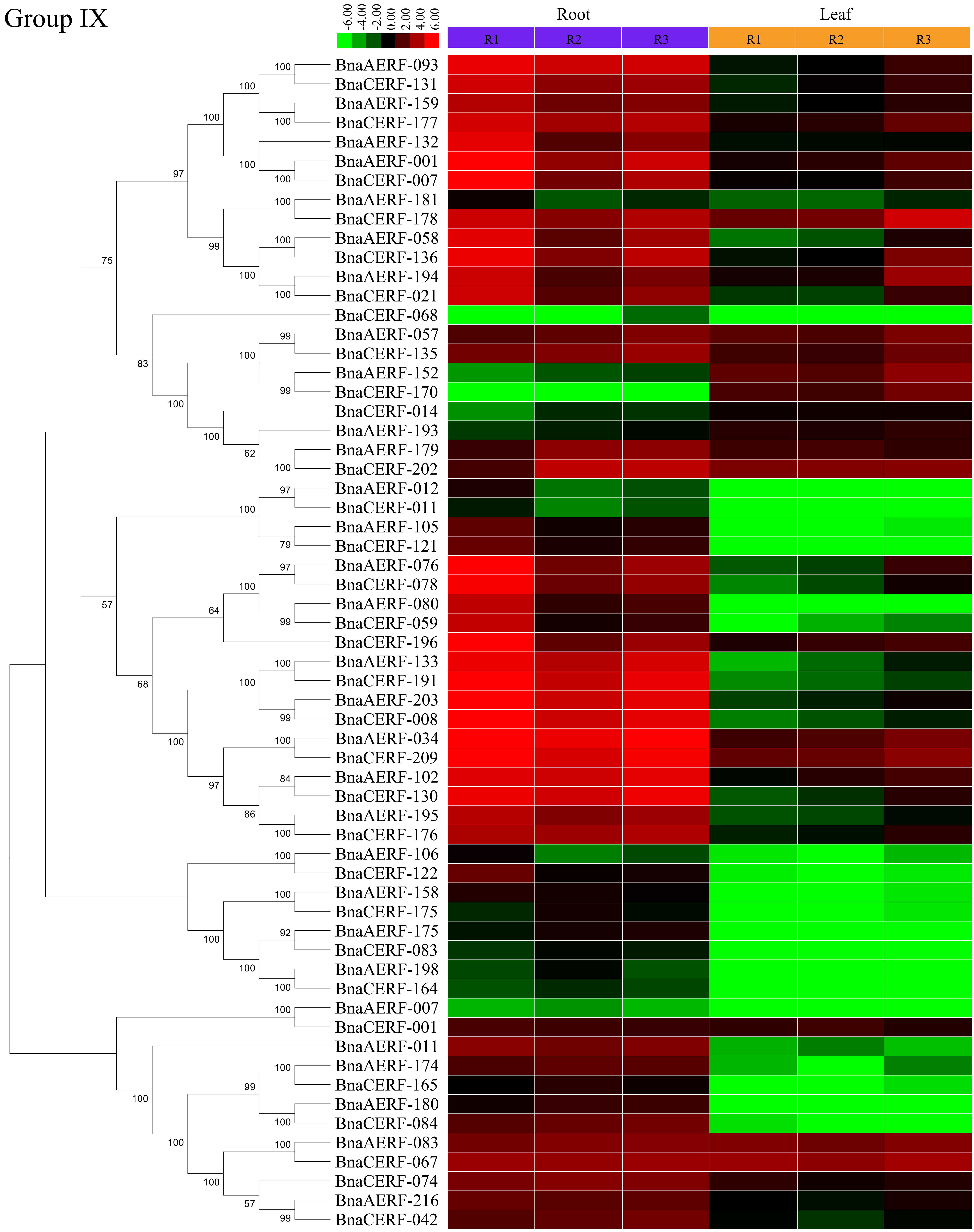

Group IX
-6.00 -4.00 -2.00 0.00 2.00 4.00 6.00
Root
Leaf
R1 R2 R3 R1 R2 R3
BnaAERF-093
BnaCERF-131
BnaAERF-159
BnaCERF-177
BnaAERF-132
BnaAERF-001
BnaCERF-007
BnaAERF-181
BnaCERF-178
BnaAERF-058
BnaCERF-136
BnaAERF-194
BnaCERF-021
BnaCERF-068
BnaAERF-057
BnaCERF-135
BnaAERF-152
BnaCERF-170
BnaCERF-014
BnaAERF-193
BnaAERF-179
BnaCERF-202
BnaAERF-012
BnaCERF-011
BnaAERF-105
BnaCERF-121
BnaAERF-076
BnaCERF-078
BnaAERF-080
BnaCERF-059
BnaCERF-196
BnaAERF-133
BnaCERF-191
BnaAERF-203
BnaCERF-008
BnaAERF-034
BnaCERF-209
BnaAERF-102
BnaCERF-130
BnaAERF-195
BnaCERF-176
BnaAERF-106
BnaCERF-122
BnaAERF-158
BnaCERF-175
BnaAERF-175
BnaCERF-083
BnaAERF-198
BnaCERF-164
BnaAERF-007
BnaCERF-001
BnaAERF-011
BnaAERF-174
BnaCERF-165
BnaAERF-180
BnaCERF-084
BnaAERF-083
BnaCERF-067
BnaCERF-074
BnaAERF-216
BnaCERF-042

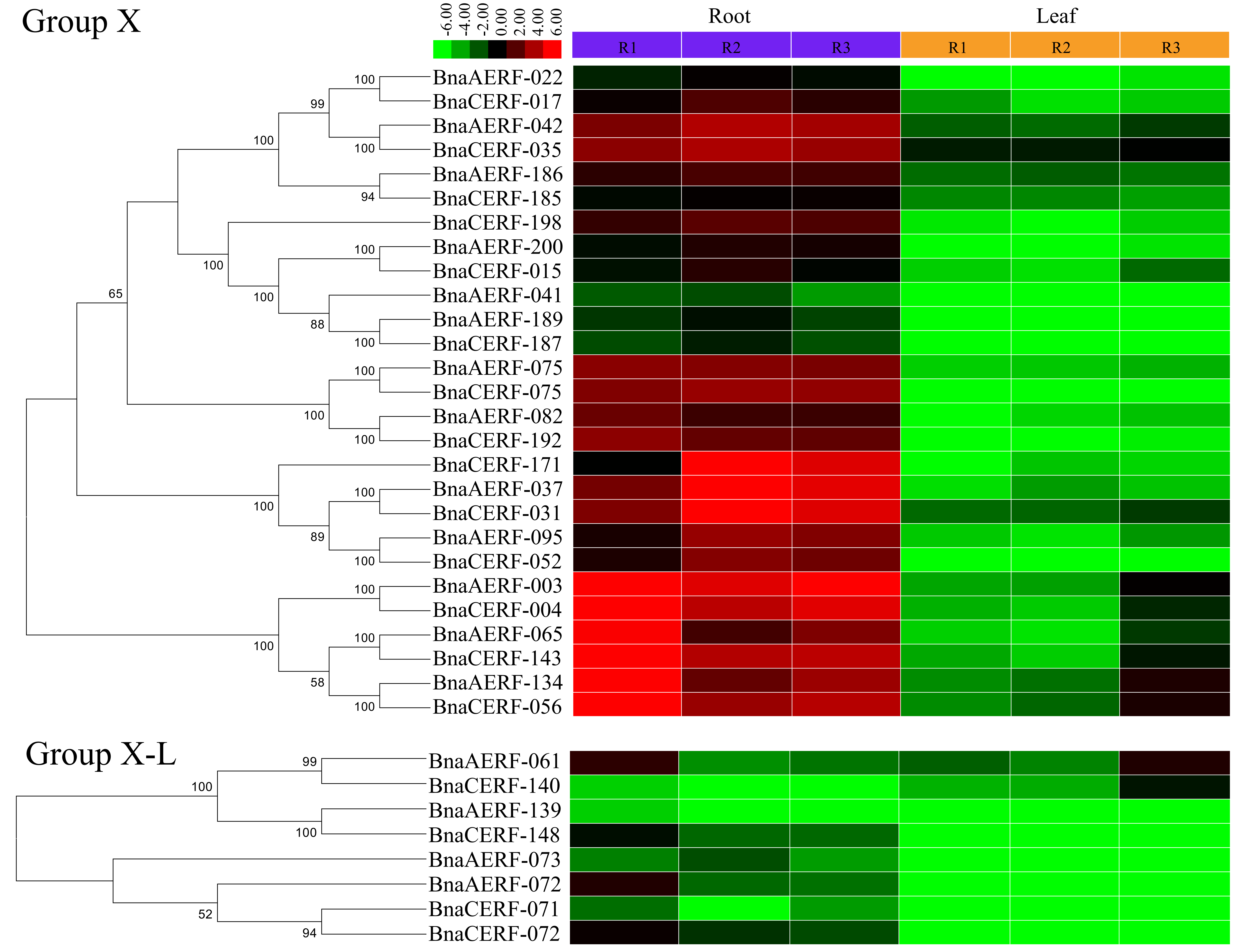
Group X
-6.00
-4.00
-2.00
0.00
2.00
4.00
6.00
Root
Leaf
R1
R2
R3
R1
R2
R3
BnaAERF-022
BnaCERF-017
BnaAERF-042
BnaCERF-035
BnaAERF-186
BnaCERF-185
BnaCERF-198
BnaAERF-200
BnaCERF-015
BnaAERF-041
BnaAERF-189
BnaCERF-187
BnaAERF-075
BnaCERF-075
BnaAERF-082
BnaCERF-192
BnaCERF-171
BnaAERF-037
BnaCERF-031
BnaAERF-095
BnaCERF-052
BnaAERF-003
BnaCERF-004
BnaAERF-065
BnaCERF-143
BnaAERF-134
BnaCERF-056
Group X-L
BnaAERF-061
BnaCERF-140
BnaAERF-139
BnaCERF-148
BnaAERF-073
BnaAERF-072
BnaCERF-071
BnaCERF-072

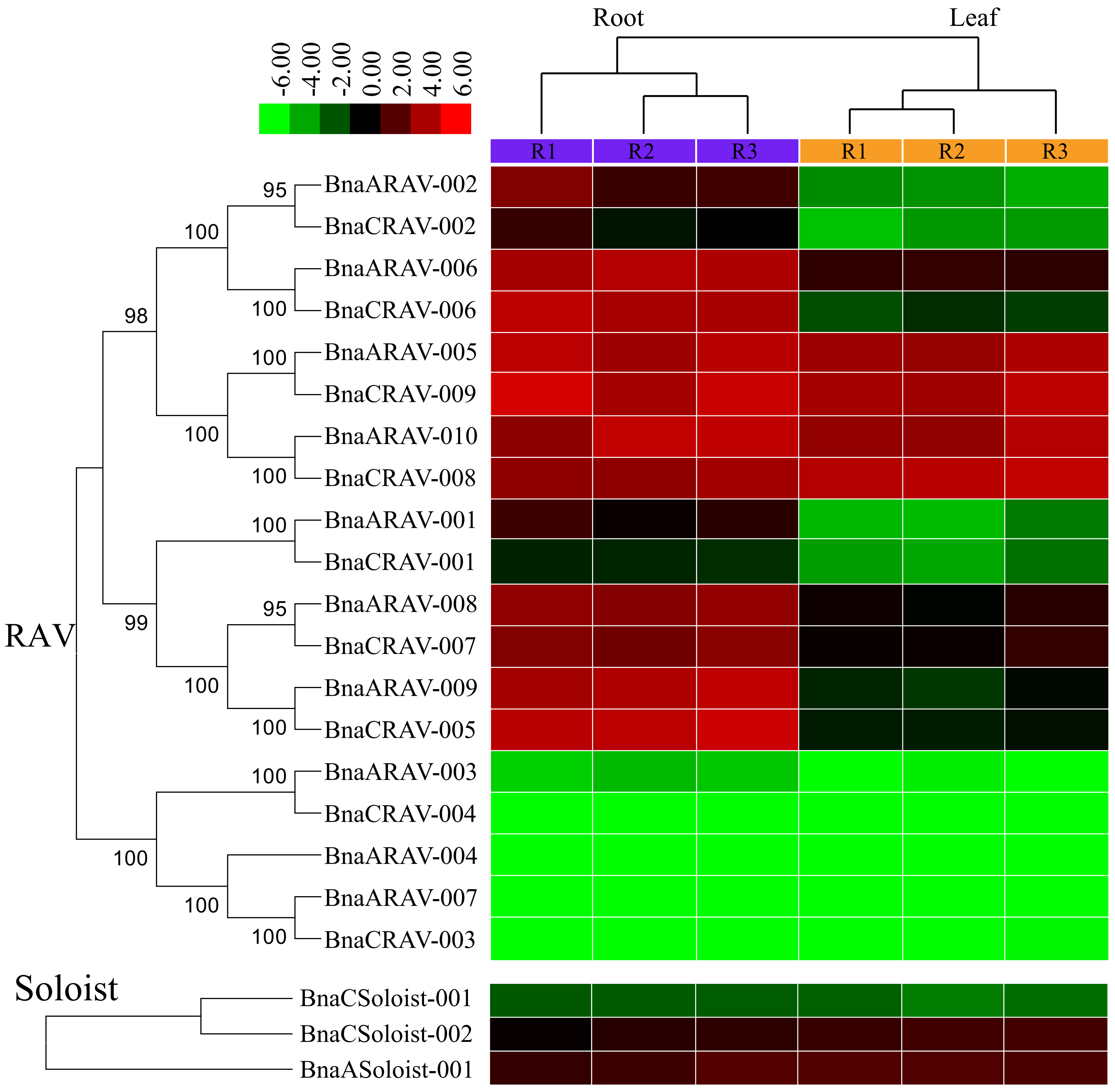

Root
Leaf
-6.00
-4.00
-2.00
0.00
2.00
4.00
6.00
R1
R2
R3
R1
R2
R3
BnaARAV-002
BnaCRAV-002
BnaARAV-006
BnaCRAV-006
BnaARAV-005
BnaCRAV-009
BnaARAV-010
BnaCRAV-008
BnaARAV-001
BnaCRAV-001
BnaARAV-008
BnaCRAV-007
BnaARAV-009
BnaCRAV-005
BnaARAV-003
BnaCRAV-004
BnaARAV-004
BnaARAV-007
BnaCRAV-003
95
100
98
100
100
100
100
100
99
95
100
100
100
100
100
100
RAV
Soloist
BnaCSoloist-001
BnaCSoloist-002
BnaASoloist-001
